# Early childhood suspected developmental delay in 63 low- and middle-income countries: Large within- and between-country inequalities documented using national health surveys

**DOI:** 10.7189/jogh.10.010427

**Published:** 2020-06

**Authors:** Jesus DC Gil, Fernanda Ewerling, Leonardo Z Ferreira, Aluisio JD Barros

**Affiliations:** 1 International Center for Equity in Health, Federal University of Pelotas, Pelotas, Brazil

## Abstract

^2^Postgraduate Program in Epidemiology, Federal University of Pelotas, Pelotas, Brazil

**Background:**

The Sustainable Development Goals call for inclusive, equitable and quality learning opportunities for all. This is especially important for children, to ensure they all develop to their full potential. We studied the prevalence and inequalities of suspected delay in child development in 63 low- and middle-income countries.

**Methods:**

We used the early child development module from national health surveys, which covers four developmental domains (physical, social-emotional, learning, literacy-numeracy) and provides a combined indicator (early child development index, ECDI) of whether children are on track. We calculated the age-adjusted prevalence of suspected delay at the country level and stratifying by wealth, urban/rural residence, sex of the child and maternal education. We also calculated measures of absolute and relative inequality.

**Results:**

We studied 330 613 children from 63 countries. Prevalence of suspected delay for the ECDI ranged from 3% in Barbados to 67% in Chad. For all countries together, 25% of the children were suspected of developmental delay. At regional level, prevalence of delay ranged from 10% in Europe and Central Asia to 42% in West and Central Africa. The literacy-numeracy domain was by far the most challenging, with the highest proportions of delay. We observed very large inequalities, and most markedly for the literacy-numeracy domain.

**Conclusions:**

To date, our study presents the most comprehensive analysis of child development using an instrument especially developed for national health surveys. With a quarter of the children globally suspected of developmental delay, we face an immense challenge. The multifactorial aspect of early child development and the large gaps we found only add to the challenge of not leaving these children behind.

Early childhood development (ECD) is a process of maturation involving the development of motor, cognitive, language and socio-emotional skills during the first years of life [1]. A delay exists when a child does not reach developmental milestones at the expected age in any dimensions of functioning [2]. Several factors increase the risk of developmental delay, among them poverty, poor parental practices, lack of child stimulation and poor nutrition, which can, in the long run, affect human capital and productivity in adulthood [3,4].

In 2016, a study updated the estimate of the number of children at risk of poor development. It concluded that approximately 43% of children under 5 years of age living in low- and middle-income countries (LMIC), nearly 250 million children, were at risk of not achieving their full potential [5]. That meant a modest reduction from a previous study published in 2007 [6]. However, because of the lack of specific data on child development in most of these countries, both studies used prevalence of stunting and extreme poverty as proxies to estimate the number of children at risk [5,6].

In 2009, the Early Childhood Development Index (ECDI) was introduced in the Multiple Indicator Cluster Surveys (MICS), and more recently in Demographic Health Surveys (DHS). The ECDI is based on a 10-item instrument covering four domains of development: physical, social-emotional, learning and literacy-numeracy. Using the ECDI, a study estimated that 33% of children from 35 LMIC were not reaching their full cognitive or social-emotional developmental potential [7]. Other studies have also used the ECDI, evidencing the importance of availability of children’s books [8], economic status [9], parents education and interaction with the child [9-11] to improve their chances to develop properly. Besides that, it has been shown that less than half the fathers engage in stimulation activities in LMIC, [10] with the poorest children having the lowest engagement of both mothers and fathers in these activities [9].

Child development is part of the transformative agenda to 2030, making it an international priority. Sustainable Development Goal 4 states that all children should have the opportunity to reach their full developmental potential [12]. The increasing number of surveys that include the ECDI, in both MICS and DHS surveys will allow for the monitoring of progress made in the area. This is made more relevant since we have evidence-based strategies and interventions to improve child development, such as cognitive stimulation and use of books, among others [7,9,11-14].

The construction of the ECDI, however, has not been free of criticism. Some items have been considered too difficult, some too easy, some difficult to interpret [7]. In an attempt to improve its validity, some publications tried alternative scoring approaches, either by dropping items or using a different classification algorithm [7,9,11].

Despite its shortcomings, the availability of the ECDI for LMIC allows a comparable analysis not only of national proportions of children with a suspected delay in ECD, but also the assessment of how population subgroups differ. Such inequalities are a priority for monitoring within the Sustainable Development Goals framework, given the motto “leave no one behind”. The literature is still lacking a more comprehensive picture of levels and inequalities in poor child development. Thus, this work aimed at assessing the proportions of children with suspected developmental delay and evaluating inequalities in terms of wealth, area of residence, sex of the child and maternal education in all LMIC with available data.

## METHODS

We used data from Multiple Indicator Cluster Surveys (MICS) and Demographic and Health Surveys (DHS). These household surveys present robust, internationally comparable data for more than 100 key health and wellness indicators for women and children. Since the fourth round of MICS surveys (starting in 2009), surveys started to include a module on ECD. Since 2011, some DHS surveys also included this module. So far, 63 LMIC have data on ECD (see Table S1 in the **Online Supplementary Document** for details).

The ECDI is an indicator based on a 10-item instrument responded by the mothers or caregivers of children aged 36-59 months [15]. The questions are divided into four domains:

- Physical: (i) child can pick up small objects with two fingers, like a stick or a rock from the ground; (ii) child is not sometimes too sick to play.
- Social-emotional: (i) child gets along well with other children; (ii) child does not kick, bite or hit other children; (iii) child does not get distracted easily.
- Learning: (i) child can follow simple directions on how to do something correctly; (ii) when given something to do, the child is able to do it independently.
- Literacy-numeracy: (i) child can read at least four simple, popular words; (ii) can identify/name at least ten letters of the alphabet; (iii) knows the name and recognizes the symbols of all numbers 1-10.

For each of the domains, a child is considered on track if she passes on two, one, two and one items, respectively. The ECDI considers a child developmentally on track if she is on track in at least three of the four domains. Given our interest in children that are being left behind, the outcome of our study was child not on track, that is, suspected of a developmental delay.

Because the items are the same for the age range 36-59 months, the performance in the test will improve with age. Thus, we estimated the age-adjusted prevalence of suspected delay for each ECD domain and for the ECDI using logistic regressions, adjusting for the child’s age. We then estimated the adjusted prevalence of suspected delay for a fixed age of 48 months (the midpoint of the study age range). The adjusted estimates were calculated at national level and for a set of stratifiers: urban or rural area of residence, maternal education in three groups (none, primary, secondary or higher), sex of the child and wealth quintiles.

The wealth classification for households was based on a wealth index that considered asset ownership, household characteristics and access to facilities such as electricity or piped water. This index is estimated through principal components analysis and is adjusted for urban or rural residence. Households are then categorized into quintiles of the resulting score, Q1 including the 20% poorest households up to Q5 with the 20% richest households [16].

Wealth related inequality was measured through two indicators, the slope index of inequality (SII) for absolute inequality and the concentration index (CIX) for relative inequality. The SII represents the difference, in percentage points, between the estimated prevalence of developmental delay for the top and bottom of the wealth distribution. The SII was estimated through logistic regression [17]. Similarly to the Gini coefficient, the CIX can be represented in the form of a Lorenz curve that shows the sample ranked by wealth on the x-axis, and the cumulative distribution of outcome on the y-axis. The CIX is calculated as twice the area between the concentration curve and the diagonal line [17]. Both the SII and the CIX are expressed on a scale of -100 to +100, where zero represents the equitable distribution of the attribute, a positive value means the outcome is concentrated towards the rich and a negative value means the outcome is concentrated towards the poor [17].

Pooled results at regional level and including all studied countries are unweighted averages of the country-specific estimates. By doing so, each country had the same weight, and we obtained the average proportions across countries, globally or within a given region.

The analyses were performed with Stata (StataCorp. 2017. Stata Statistical Software: Release 15.1. College Station, TX,USA: StataCorp LLC). All the analyses considered the surveys’ sample design. DHS and MICS are public sources of information and ethical approval was already obtained by the institutions responsible for its implementation in each country.

## RESULTS

We studied 330 613 children from 63 countries, with survey years ranging from 2010 to 2016. The countries studied, survey year and types and sample sizes are listed in Table S1 in the **Online Supplementary Document**. Saint Lucia and Barbados were excluded from the stratified analyses due to insufficient sample size. Barbados, Qatar and Cuba do not have information on wealth and thus the results could not be stratified by wealth quintiles.

**Table 1** presents the prevalence of suspected delay for each developmental domain and the ECDI for the countries studied, grouped by world region. We observed huge inequalities between countries. Suspected delay for the ECDI was below 5% in Barbados, Bosnia and Herzegovina and Montenegro. On the other extreme, it was over 50% for Guinea, CAR, Sierra Leone, Burundi and Chad – all in sub-Saharan Africa. A map of these estimates can be found in Figure S1 in the **Online Supplementary Document**.

**Table 1:** Proportions of children with suspected developmental delay in each studied country for the combined indicator early child development index (ECDI) and each of the domains; countries grouped by world region*

		Domain									
	**Physical**		**95% CI**	**Social-emotional**	**95% CI**	**Learning**	**95% CI**	**Literacy-numeracy**	**95% CI**	**ECDI**	**95% CI**
**Country**	**Children with suspected developmental delay (%)**										
**West and Central Africa:**											
**Benin**	4.9		(3.7; 6.0)	28.5	(26.5; 30.5)	17.3	(15.6; 18.9)	92.4	(91.2; 93.7)	37.8	(35.8; 39.8)
**Cameroon**	2.4		(1.5; 3.3)	31.6	(29.5; 33.8)	13.6	(11.7; 15.5)	83.9	(81.5; 86.2)	36.7	(34.3; 39.0)
**CAR**	3.9		(3.0; 4.8)	42.4	(39.4; 45.3)	22.7	(20.5; 24.9)	92.7	(91.1; 94.2)	51.7	(49.0; 54.4)
**Chad**	16.5		(14.8; 18.1)	40.4	(37.8; 43.0)	45.4	(42.5; 48.3)	94.6	(93.5; 95.6)	67.3	(64.7; 70.0)
**Congo Brazzaville**	2.8		(2.0; 3.5)	36.1	(33.3; 38.9)	14.9	(12.8; 16.9)	86.3	(84.3; 88.3)	39.1	(36.0; 42.1)
**Congo DR**	7.0		(5.6; 8.5)	21.2	(18.0; 24.5)	19.2	(16.3; 22.0)	89.2	(87.1; 91.3)	34.0	(30.2; 37.8)
**Côte d’lvoire**	3.2		(2.2; 4.1)	31.0	(28.5; 33.5)	11.0	(8.7; 13.2)	92.5	(90.7; 94.3)	36.2	(33.4; 39.1)
**Gambia**	2.1		(1.4; 2.7)	31.3	(28.7; 33.9)	4.7	(3.6; 5.8)	87.7	(85.4; 89.9)	30.4	(27.8; 32.9)
**Ghana**	2.6		(1.8; 3.4)	26.6	(24.0; 29.1)	10.6	(8.6; 12.5)	71.6	(68.6; 74.6)	24.9	(22.1; 27.6)
**Guinea**	7.5		(6.1; 9.0)	38.5	(36.1; 40.8)	19.0	(16.7; 21.4)	94.2	(92.8; 95.5)	50.5	(47.8; 53.3)
**Guinea Bissau**	10.5		(8.1; 12.8)	26.5	(23.8; 29.3)	11.5	(9.8; 13.1)	92.4	(90.6; 94.3)	37.7	(34.5; 41.0)
**Mali**	4.6		(3.8; 5.4)	27.1	(25.4; 28.9)	13.1	(11.6; 14.5)	91.4	(90.2; 92.7)	37.1	(35.0; 39.1)
**Mauritania**	7.5		(6.2; 8.7)	33.9	(31.4; 36.4)	18.6	(16.4; 20.8)	72.5	(70.2; 74.9)	38.4	(36.1; 40.8)
**Nigeria**	9.5		(8.7; 10.4)	28.6	(27.2; 30.0)	21.2	(19.7; 22.6)	70.3	(68.4; 72.2)	37.9	(36.1; 39.6)
**São Tome e Príncipe**	4.7		(2.7; 6.8)	37.5	(33.6; 41.4)	19.7	(16.4; 23.1)	84.3	(80.9; 87.8)	45.0	(40.6; 49.4)
**Sierra Leone**	10.7		(9.2; 12.2)	40.8	(37.9; 43.7)	22.2	(20.2; 24.3)	90.5	(89.1; 92.0)	54.0	(51.2; 56.8)
**Togo**	6.6		(5.1; 8.2)	24.3	(22.0; 26.7)	28.6	(25.7; 31.4)	92.3	(90.6; 93.9)	45.2	(42.1; 48.2)
**Eastern and South Africa:**											
**Burundi**	7.4		(6.5; 8.4)	40.6	(38.3; 42.9)	35.9	(33.7; 38.1)	91.3	(90.2; 92.4)	59.2	(57.2; 61.3)
**Eswatini**	6.7		(4.9; 8.4)	34.8	(31.1; 38.4)	4.9	(3.1; 6.8)	82.7	(79.8; 85.5)	33.6	(30.2; 37.1)
**Malawi**	9.5		(8.4; 10.7)	28.2	(26.6; 29.8)	18.8	(17.3; 20.3)	82.8	(81.3; 84.2)	39.1	(37.4; 40.9)
**Rwanda**	4.9		(3.9; 5.9)	17.9	(15.5; 20.3)	13.2	(11.5; 14.8)	92.9	(91.6; 94.1)	28.4	(25.9; 30.8)
**Uganda**	9.4		(8.1; 10.7)	32.3	(30.5; 34.2)	13.3	(11.8; 14.8)	71.0	(68.9; 73.1)	34.8	(32.7; 36.8)
**Zimbabwe**	5.4		(4.4; 6.4)	32.4	(30.5; 34.3)	10.2	(8.8; 11.5)	91.4	(90.4; 92.5)	36.8	(35.0; 38.7)
**Middle East and North Africa:**											
**Algeria**	4.1		(3.3; 4.9)	29.5	(27.6; 31.4)	9.8	(8.6; 10.9)	71.2	(69.0; 73.4)	29.2	(27.4; 31.0)
**Iraq**	5.1		(4.4; 5.7)	22.3	(21.0; 23.5)	10.4	(9.4; 11.3)	82.0	(80.5; 83.6)	27.7	(26.3; 29.1)
**Jordan**	1.1		(0.5; 1.6)	29.0	(25.6; 32.3)	9.2	(7.6; 10.8)	83.6	(80.9; 86.2)	30.4	(27.2; 33.6)
**Qatar**	6.1		(3.7; 8.5)	23.7	(19.4; 28.0)	11.1	(7.6; 14.6)	33.3	(27.2; 39.4)	13.4	(9.4; 14.4)
**State of Palestine**	1.9		(0.9; 2.8)	28.2	(26.4; 30.0)	7.1	(6.0; 8.3)	81.2	(79.4; 82.9)	26.5	(24.8; 28.1)
**Tunisia**	3.1		(1.9; 4.3)	24.3	(21.3; 27.4)	6.3	(4.6; 8.1)	67.5	(63.6; 71.5)	22.1	(19.0; 25.1)
**Europe and Central Asia:**											
**Belarus**	0.6		(0.1; 1.1)	10.3	(8.2; 12.3)	0.0	(0.0; 0.0)	52.2	(48.3; 56.1)	5.4	(3.9; 7.0)
**Bosnia and Herzegovina**	0.3		(0.0; 0.6)	4.6	(2.4; 6.7)	0.9	(0.3; 1.5)	74.5	(70.2; 78.9)	3.3	(1.5; 5.1)
**Kazakhstan**	0.5		(0.0; 1.1)	17.0	(14.4; 19.5)	1.8	(0.9; 2.7)	72.8	(69.9; 75.7)	13.1	(10.5; 15.6)
**Kosovo**	1.1		(0.0; 2.7)	15.5	(12.5; 18.5)	3.0	(1.2; 4.9)	82.6	(78.7; 86.5)	15.8	(13.0; 18.7)
**Kyrgyzstan**	2.3		(1.1; 3.4)	16.1	(14.0; 18.2)	6.2	(4.1; 8.2)	86.1	(83.7; 88.5)	19.7	(17.1; 22.3)
**Macedonia**	0.0		(0.0; 0.1)	8.8	(6.0; 11.5)	1.4	(0.4; 2.5)	57.1	(51.9; 62.4)	6.9	(4.4; 9.5)
**Moldova**	0.4		(0.0; 1.1)	20.4	(16.9; 23.9)	0.3	(0.0; 1.0)	70.7	(66.9; 74.6)	15.0	(11.9; 18.2)
**Montenegro**	0.0		(0.0; 0.0)	5.1	(2.7; 7.4)	1.0	(0.0; 2.4)	78.8	(73.9; 83.7)	4.4	(2.3; 6.5)
**Turkmenistan**	0.3		(0.0; 0.9)	5.4	(3.9; 6.8)	2.6	(1.0; 4.3)	82.7	(79.5; 85.9)	7.7	(6.0; 9.4)
**Ukraine**	0.5		(0.0; 1.1)	16.0	(13.3; 18.7)	1.3	(0.6; 2.0)	54.3	(49.7; 58.9)	9.3	(7.3; 11.3)
**South Asia:**											
**Bangladesh**	7.3		(6.3; 8.2)	31.3	(30.0; 32.7)	12.0	(11.0; 13.1)	79.6	(78.2; 80.9)	35.4	(33.9; 36.8)
**Bhutan**	1.8		(1.1; 2.5)	29.6	(27.1; 32.2)	6.1	(4.7; 7.4)	75.0	(72.2; 77.8)	27.4	(24.7; 30.0)
**Nepal**	3.5		(2.5; 4.6)	31.4	(28.7; 34.0)	17.8	(14.6; 21.1)	71.7	(68.0; 75.4)	35.1	(31.4; 38.8)
**East Asia and Pacific:**											
**Cambodia**	3.5		(2.5; 4.4)	25.6	(23.4; 27.8)	10.2	(8.7; 11.7)	72.9	(70.1; 75.7)	26.6	(24.3; 29.0)
**Lao**	1.8		(1.3; 2.3)	14.7	(13.3; 16.2)	6.1	(4.8; 7.3)	80.1	(78.1; 82.2)	18.4	(16.8; 20.1)
**Mongolia**	0.6		(0.0; 1.1)	23.8	(21.8; 25.8)	1.7	(1.0; 2.4)	91.5	(90.1; 92.9)	23.2	(21.2; 25.1)
**Thailand**	1.5		(0.1; 2.8)	20.0	(17.4; 22.6)	0.4	(0.0; 0.9)	28.0	(25.1; 31.0)	6.7	(5.3; 8.2)
**Vietnam**	2.4		(1.1; 3.8)	7.6	(5.5; 9.8)	4.8	(3.1; 6.4)	71.9	(68.5; 75.4)	10.0	(7.8; 12.2)
**Latin America and the Caribbean:**											
**Argentina**	1.5		(0.8; 2.3)	18.2	(16.2; 20.3)	2.1	(1.2; 3.0)	58.4	(55.5; 61.3)	13.2	(11.4; 15.0)
**Barbados**	0.0		-	19.6	(14.1; 25.0)	0.2	(0.0; 0.6)	8.7	(4.5; 13.0)	3.2	(0.4; 5.9)
**Belize**	1.8		(0.2; 3.5)	22.6	(19.3; 25.8)	4.8	(2.4; 7.2)	46.2	(41.2; 51.1)	15.6	(12.7; 18.6)
**Costa Rica**	1.1		(0.2; 1.9)	20.4	(16.0; 24.9)	0.4	(0.0; 0.8)	74.3	(68.2; 80.5)	18.2	(13.7; 22.6)
**Cuba**	0.2		(0.0; 0.9)	10.8	(8.0; 13.5)	1.8	(0.0; 4.7)	79.2	(72.6; 85.7)	9.9	(7.0; 12.9)
**Dominican Republic**	2.0		(1.3; 2.7)	14.9	(13.6; 16.2)	2.0	(1.4; 2.7)	79.4	(77.8; 81.0)	14.5	(13.2; 15.8)
**El Salvador**	2.1		(1.3; 2.8)	19.3	(17.4; 21.1)	2.4	(1.3; 3.4)	81.6	(79.3; 83.9)	18.2	(16.4; 19.9)
**Guyana**	1.7		(0.7; 2.6)	25.0	(22.1; 27.9)	3.5	(2.0; 5.0)	33.5	(30.1; 36.9)	11.4	(9.1; 13.7)
**Jamaica**	1.3		(0.2; 2.5)	21.3	(17.0; 25.5)	2.7	(1.3; 4.2)	33.5	(27.9; 39.1)	10.0	(7.3; 12.7)
**Mexico**	0.6		(0.0; 1.3)	21.1	(18.2; 23.9)	1.3	(0.7; 1.9)	78.2	(74.7; 81.7)	17.3	(14.7; 19.9)
**Panama**	0.7		(0.2; 1.1)	18.6	(15.1; 22.0)	3.7	(2.3; 5.1)	81.2	(77.6; 84.7)	18.9	(15.6; 22.3)
**Paraguay**	1.2		(0.3; 2.1)	17.9	(15.5; 20.3)	3.1	(1.6; 4.5)	77.2	(74.2; 80.2)	17.2	(14.7; 19.7)
**St Lucia**	1.1		(0.0; 3.2)	12.8	(6.4; 19.2)	1.2	(0.0; 3.0)	23.4	(13.4; 33.3)	6.9	(1.7; 12.1)
**Suriname**	2.1		(1.3; 3.0)	32.6	(29.1; 36.0)	2.8	(1.7; 3.9)	79.1	(75.8; 82.5)	28.7	(25.4; 32.0)
**Trinidad and Tobago**	1.6		(0.4; 2.8)	20.1	(15.8; 24.4)	3.0	(1.1; 4.8)	17.2	(12.9; 21.5)	6.0	(3.2; 8.8)
**Uruguay**	2.1		(0.0; 4.3)	19.8	(12.0; 27.6)	1.8	(0.0; 3.8)	50.1	(40.8; 59.4)	12.7	(6.0; 19.4)

CI – confidence interval, ECDI – early child development index

*Source: MICS and DHS surveys, 2010-2016.

The literacy-numeracy domain presented the highest levels of delay. Barbados, Trinidad and Tobago, St Lucia, Thailand, Qatar, Jamaica and Guyana presented the lowest prevalence, below 40%. On the other extreme, 29 countries had more than 80% of the children suspected of delay. Prevalence of suspected delay in the social-emotional domain ranged from 4.6% in Bosnia-Herzegovina to 42.4% in CAR. Again, the countries with lowest levels of suspected delay were from Europe and Central Asia, East Asia and Pacific and Latin America and Caribbean, while the highest levels were seen in sub-Saharan Africa. The learning domain had levels of suspected delay below 10% for most countries, and only 6 countries had a prevalence over 20% - Nigeria, Sierra Leone, CAR, Togo, Burundi and Chad. The physical domain presented the lowest proportions of children with suspected delay, ranging from zero in Barbados, Macedonia and Montenegro, to 16.5% in Chad.

**Figure 1:**
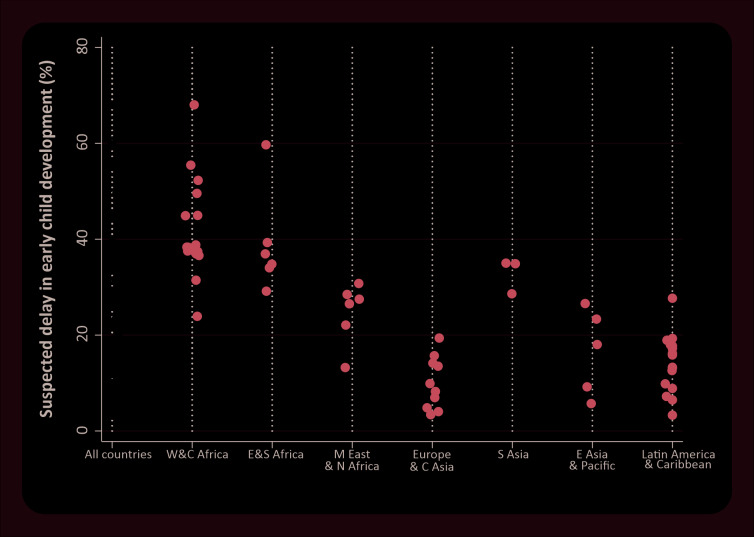
Prevalence of suspected developmental delay (ECDI) in each studied country, all countries together and grouped by world region. Source: MICS and DHS surveys, 2010-2016.

Even within regions, we observed large between-country inequalities. In West and Central Africa, the region with the highest average prevalence of suspected delay in the ECDI, country estimates ranged from 24.9% in Ghana to 67.3% in Chad. Inequalities across all countries, and inequalities by world region can be better appreciated in **Figure 1**. West and Central Africa was not only the region with highest levels of delay, but also where between-country inequality was greatest. The other regions also presented large gaps between countries, except for South Asia with only three countries represented.

**Table 2** shows the average prevalence of suspected developmental delay by world regions, according to the UNICEF classification. The estimates are presented by domain and for ECDI. It is clear from the table that the level of difficulty of each domain is different. Literacy-numeracy was the most challenging, with the highest levels of suspected delay. The social-emotional domain was second, followed by learning and physical.

**Table 2:** Average proportions of children with suspected developmental delay in each world region for the combined indicator early child development index (ECDI) and each of the domains*

		Domain				
		**Physical**	**Social-emotional**	**Learning**	**Literacy-numeracy**	**ECDI**
**World region**	**N of countries**	**Children with suspected developmental delay (%)**				
West and Central Africa	17	6.3	32.1	18.4	87.0	41.4
Eastern and Southern Africa	6	7.2	31.0	16.1	85.4	38.7
Middle East and North Africa	6	3.6	26.2	9.0	69.8	24.9
Europe and Central Asia	10	0.6	11.9	1.9	71.2	10.1
South Asia	3	4.2	30.8	12.0	75.4	32.6
East Asia and Pacific	5	2.0	18.3	4.6	68.9	17.0
Latin America and the Caribbean	16	1.3	19.7	2.3	56.3	13.9
**Income group:**						
Low income	18	7.0	31.7	18.6	87.2	41.2
Lower middle income	19	3.2	25.4	8.7	77.9	26.6
Upper middle income	22	1.2	17.5	2.8	63.4	13.9
High income	4	2.2	19.8	3.6	36.6	9.7
**All countries**	63	3.5	24.0	9.2	72.9	25.3

ECDI – early child development index

*Source: MICS and DHS surveys, 2010-2016.

The regions with the largest numbers of countries were West and Central Africa and Latin America and the Caribbean, with 17 and 16 countries respectively. West and Central Africa presented the highest prevalence of suspected delay for ECDI (41.4%), and for the social-emotional (32.1%), learning (18.4%) and literacy-numeracy (87.0%) domains. The physical domain presented much lower levels of suspected delay compared to the others, and the highest prevalence observed was in Eastern and Southern Africa (7.2%).

The countries in Europe and Central Asia had the lowest prevalence of suspected delay for ECDI (10.1%) and for the physical (0.6%), learning (1.9%) and social-emotional (11.9%) domains. For the literacy-numeracy domain the lowest prevalence was in Latin America and the Caribbean (56.3%) (**Table 2**).

Prevalence of suspected delay generally decreased with increasing income group. Low-income countries had the highest prevalence for the 4 domains and for the ECDI (41.2%). The most striking difference was observed for literacy-numeracy, where the average prevalence of suspected delay ranged from 36.6% in high income countries to 87.2% in low income countries (**Table 2**).

The estimates of suspected delay for the four domains and ECDI stratified by wealth quintiles, plus the SII and the CIX (absolute and relative within-country inequality, respectively) are presented in Tables S2-S6 in the **Online Supplementary Document**. Both the SII and the CIX present negative values in most countries, meaning that the prevalence of suspected delay is concentrated towards the poor. For the ECDI, SII ranged from -46.8 percentage points in Nigeria to 12.1 percentage points in Guinea Bissau, while the CIX ranged from -29.8 in Uruguay to 1.3 in Guinea Bissau. We also assessed the prevalence of suspected delay by urban/rural area of residence, sex of the child and maternal education. These results are presented in Tables S7-11 in the **Online Supplementary Document****,** for details.

Nigeria (SII = -46.8), Uganda (SII = -13.1), Palestine (SII = 17.3), Moldova (SII = -13.5), Nepal (SII = -14.2) and Costa Rica (SII = -20.2) present, each, the highest level of wealth inequalities in ECDI in their respective regions (Table S6 in the **Online Supplementary Document** for details). On the low inequality spectrum, Europe and Central Asia is the region where countries present the smallest inequalities. This is also the region with the lowest levels of suspected delay (**Table 2**).

**Figure 2:**
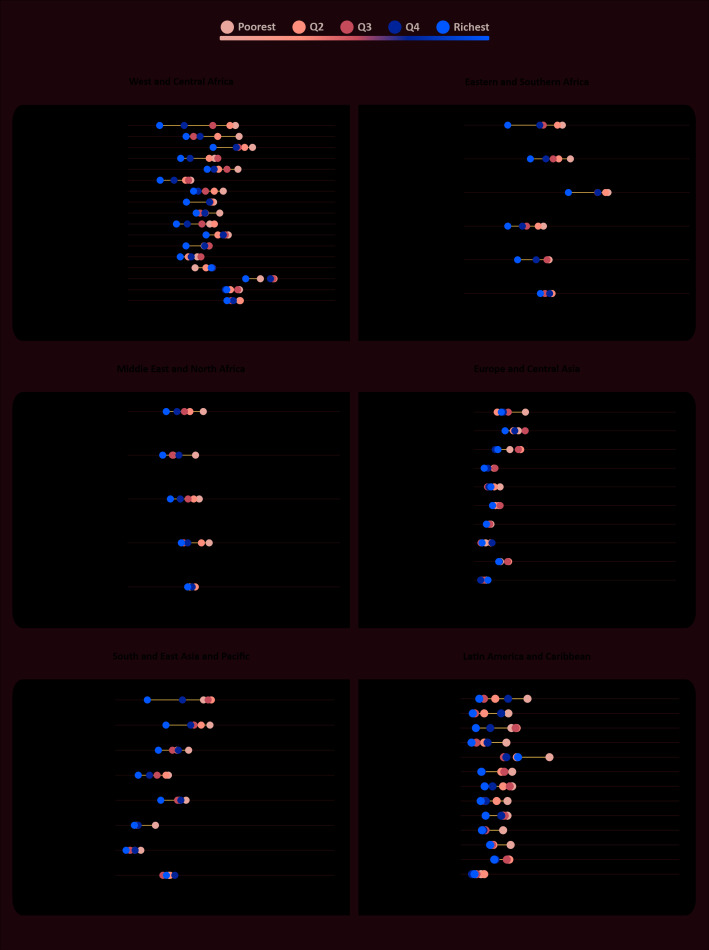
Prevalence of suspected developmental delay (ECDI) by wealth quintiles, countries grouped by world region. Source: MICS and DHS surveys, 2010-2016.

**Figure 3:**
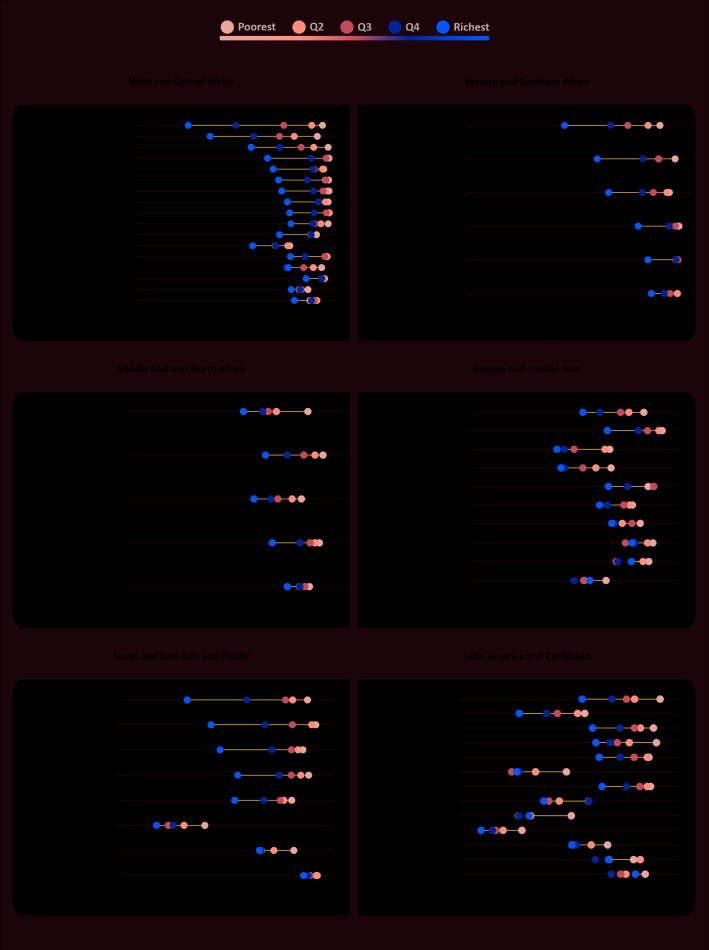
Prevalence of suspected developmental delay (literacy-numeracy domain) by wealth quintiles, countries grouped by world region. Source: MICS and DHS surveys, 2010-2016.

**Figure 2** and **Figure 3** show the prevalence of suspected delay for ECDI and the literacy-numeracy domain, respectively, by wealth quintiles. Examination of the performance of the children from the richest families in each country gives us a clearer picture of what could be expected in a best-case scenario. Only five countries presented levels of suspected delay below 30% for literacy-numeracy among the richest group – Trinidad and Tobago, Thailand, Guyana, Belize and Nigeria, in increasing order of prevalence. In terms of inequality, **Figure 2** and **Figure 3**show some huge gaps between richest and poorest. For the literacy-numeracy domain, the richest quintile presents prevalence that is markedly lower than the other groups. Nigeria, Nepal, Ghana and Lao have all more than a 50 percent point difference between the extremes of wealth for literacy-numeracy. In several countries the distance between the Q5 and Q4 is more than 25 percentage points. Prevalence by wealth quintile for the other domains are presented in Figures S2-S4 in the **Online Supplementary Document**, showing much smaller inequalities for these domains.

Regarding the other stratifiers, girls presented systematically lower prevalence of suspected delay in the ECDI, with an advantage of up to 12 percentage points. Children in urban areas also presented an advantage over those from rural areas, except in 10 of 62 countries. In the most extreme cases, children from urban areas had an advantage of approximately 20 percentage point (Nepal and Congo Brazzaville). Children from mothers of higher levels of education also presented lower levels of suspected delay compared to the lower education groups (Table S11 in the **Online Supplementary Document**).

## DISCUSSION

Our study presents, to date, the most comprehensive assessment of ECD at global level using household surveys. We studied 63 low- and middle-income countries spread from East Asia, through Europe and Africa to Latin America and the Caribbean. We found huge variation in suspected developmental delay across countries, from nearly 70% in Chad to around 3% in Barbados and Bosnia and Herzegovina. This is undoubtedly one of the widest gaps in any indicator studied.

Along with the staggering between country inequalities, we also documented important within-country gaps, with absolute inequality measured by the SII of up to 47 percentage points between the extremes of the wealth spectrum. Most often, wealth inequalities were not that large, with the median SII around 13 percentage points. The overall levels of suspected delay, however, are high, and for the 63 LMICs with data, we estimated that around 23% of the children 36-59 months of age were not developmentally on track. Even though some children with suspected delay may have developmental or physical disabilities, it is important to mention that this is not necessarily true. Suspected developmental delay is not synonymous with developmental disability.

A previous study estimating risk of poor development concluded that 43% of the children in LMICs were at risk [5]. However, because of the lack of specific data on child development in these settings at the time, this estimate was based on proxies – stunting and extreme poverty. Another study, now using the ECD module available in surveys, studied 35 LMICs, and found a lower percentage, 37% of the children performing poorly in either the cognitive or the social-emotional domain [7]. This study did not use the ECDI composite indicator as proposed by UNICEF, given their reservations about some of the items, so their estimate is also not directly comparable to ours. Their estimate of suspected delay in the social-emotional domain was, however, very close to ours, at 26%. Using the ECDI composite indicator as proposed originally by UNICEF, [15] we found an even lower overall estimate for children with a suspected delay – 22.5%. This still represents a huge number of children who are not fully developing their potential.

The differences between these estimates relate to the definition of poor development, or suspected delay, as we prefer. The study by Lu et al [5] used proxies for development while the study of McCoy et al [7] used the MICS ECD module, but created an alternative definition for poor development. Their decision was based on expert evaluation of the items included in the ECDI. According to McCoy et al., [7] the literacy-numeracy domain indicators (recognizing words, letters and numbers) are more related to training and opportunities of early schooling than ability with words and numbers itself. They also argue that the items are too difficult for the age range in study. Our results confirm that this is clearly the most challenging domain for the children given the highest levels of suspected delay in this domain, even in high income countries where still 27% of the children were not on track. The huge wealth gaps we found in this domain also reinforces the idea that the items are strongly related to training opportunities.

The physical domain is also criticized by the same authors, as being too easy for the age (pick up a small object) or meaningless (being too sick to play). The lack of discriminatory power for this domain is made clear by the very low percentages of children that are not on track – 3.4% overall, ranging from 1.2% to 7.0% across income groups. On the extreme opposite to the literacy-numeracy domain, the physical domain presents very small gaps between rich and poor, what is another indicator of its lack of power to discriminate children with a poor development.

The learning (or cognitive, as referred to by McCoy et al. [7]) domain does not perform much better. The overall percentage of suspected delay was 8.4%, varying from 2.8% to 18.6% across income groups. Except in the poorest countries, the gaps between rich and poor were very small. The social-emotional domain seems to be the most balanced given the intermediate levels of suspected delay and a more consistent pattern of wealth inequalities. This domain, like the literacy-numeracy domain, includes three items what can help improving its consistency.

Despite any shortcoming, the 10-item ECDI proposed by MICS was validated and tested in Jordan, Philippines and Kenya and it has been used to monitor global progress mainly for target 4.2 of the SDGs [15]. Aware of all of these limitations regarding the items available, we still decided to use the indicator (ECDI) as proposed by MICS. The main reason to use UNICEF’s ECDI was that it combines the four domains, and in the end, neither the ones that are too easy nor the too difficult will be determinant for the final result. Also, we thought it was important, before delving in a search for an alternative way to combine the items, that we presented the comprehensive panorama of ECD using the MICS indicator that will undoubtedly be used in many local and more limited assessments.

Further limitations in assessing ECD must be acknowledged. There are many tests proposed for assessing ECD, both for diagnose and screening. Ideally, they are carried out in a controlled environment, involve dozens (or hundreds) of items that are selected for the child’s age, such as the Bayley Scales of Infant and Toddler Development [18] or the Battelle Developmental Inventory [19,20]. In surveys, a limited number of items is used, irrespective of the exact child’s age. The test environment is the child’s home, quite variable, and the items will be subject to the mother’s interpretation, given the cultural context. In this sense, the results must be interpreted bearing in mind the context, and the difficulties in assessing ECD in such conditions. The results, despite the limitations listed, are highly valuable for setting the agenda at the global level and at country level.

To date, the ECDI is the best measure of child development available for LMIC, covering a large number of countries that otherwise would not have any information to guide policies and strategies. However, given all the limitations already exposed, a revision of the index and the questions included in the surveys is needed, so that a stronger measure of child development – or possibly a new one – that resolves all the controversies debated in the literature can be proposed. We urge for a cross-cultural tool that is more widely accepted by the academic community, but still feasible to be used in the context of the national health surveys, that provide most of the reliable data from LMIC.

Examining inequality patterns, it is clear that Eastern and Southern Africa is the region where we see the richest 20% way ahead of the rest in terms of the ECDI. Burundi and Uganda are extreme examples of top inequality. It is interesting that the opposite situation is not so common, with only a few countries presenting a pattern of bottom inequality – Suriname, Tunisia, and Vietnam. In West and Central Africa, where we found the highest levels of suspected delay, the inequality pattern is most often linear, with wealth quintiles more or less equally spaced.

The relationship between child development and poverty and scarcity of health and educational resources has been recognized for some time now [7,9,21]. Our results are consistent with this literature, with poorer countries and poorer regions presenting not only the highest levels of suspected developmental delay, but also the largest inequalities between rich and poor. We also show that rural areas are systematically worse in terms of ECD, where usually we see the lowest levels of parental education and access to school, recognized as key factors for improving ECD [9,11].

Our study provides a broad and comprehensive assessment of ECD in LMICs and highlights the importance of investing in the future of disadvantaged children, who are at higher risk of not developing to their full potential. These children are everywhere, as our results show. Of course, they are more numerous in poorer countries, and concentrated among the poorest populations within countries, in rural areas and among those with mothers with lower education levels. Considering the important impact, it may have in the future of the children, investments in national-level programs using childcare, schools and other community channels may be a solution to reach all children and improve their chances to properly develop. The picture we present calls for immediate and effective action. This is not an easy task since many studies point to several contextual and individual factors that need to be tackled to achieve progress. These factors range from poverty, and lack of basic resources [22,23] to malnutrition, the home environment and cognitive stimulation [24-27] and most likely isolated interventions may be effective but will always have a limited populational impact. Integrated interventions have, theoretically, more potential, but present bigger challenges for implementation [26]. The only unacceptable alternative is no action.

## CONCLUSIONS

To date, this study presents the most comprehensive analysis of child development using an instrument especially developed for national health surveys. With a quarter of the children globally suspected of developmental delay, we face an immense challenge. The multifactorial aspect of early child development and the large gaps we found only add to the challenge of not leaving these children behind.

## Additional material

Online Supplementary Document
