# Supplementary material for: Early childhood suspected developmental delay in 63 low- and middle-income countries: Large within- and between-country inequalities documented using national health surveys
Source: J Glob Health. 2020 Jun 11;10(1):010427. doi: 10.7189/jogh.10.010427 (PMC7295453; doi:10.7189/jogh.10.010427)
Supplement: Online Supplementary Document [file jogh-10-010427-s001.pdf]

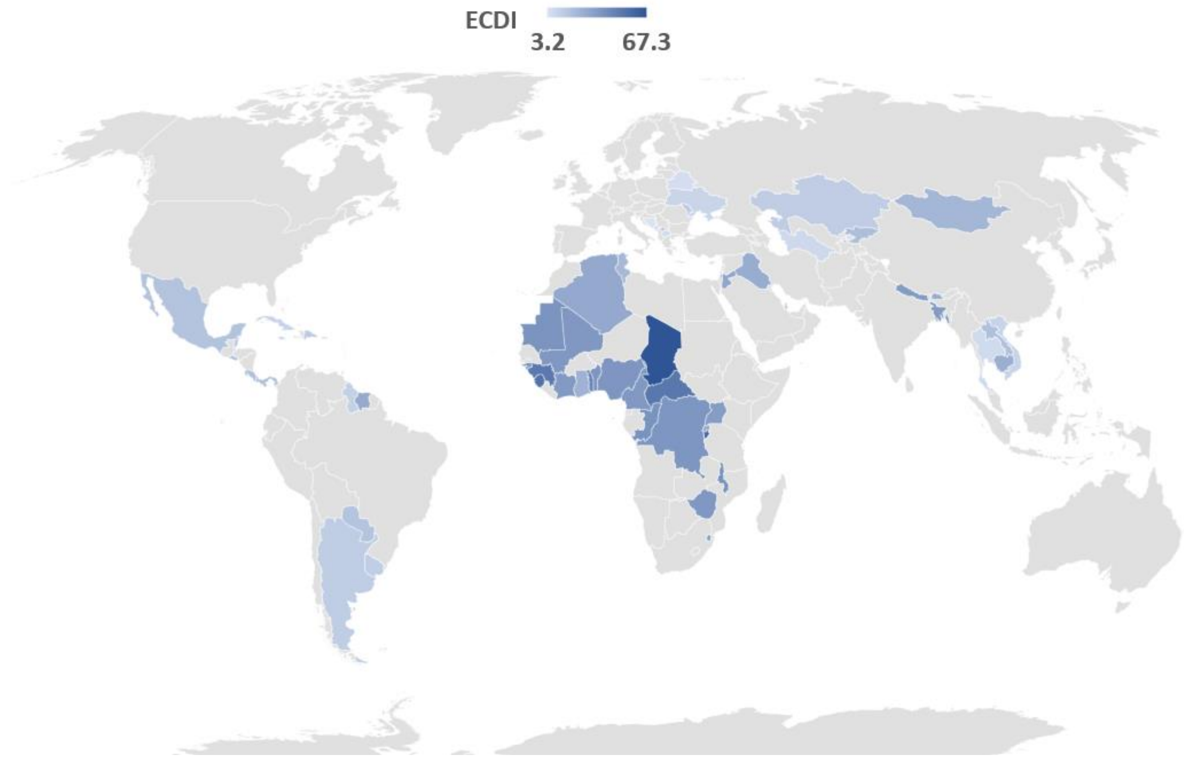

Fig S1. Prevalence of suspected developmental delay (ECDI) among children aged 36-49 months in 63 low and middle-income countries countries. Source: MICS and DHS surveys, 2010-2016.

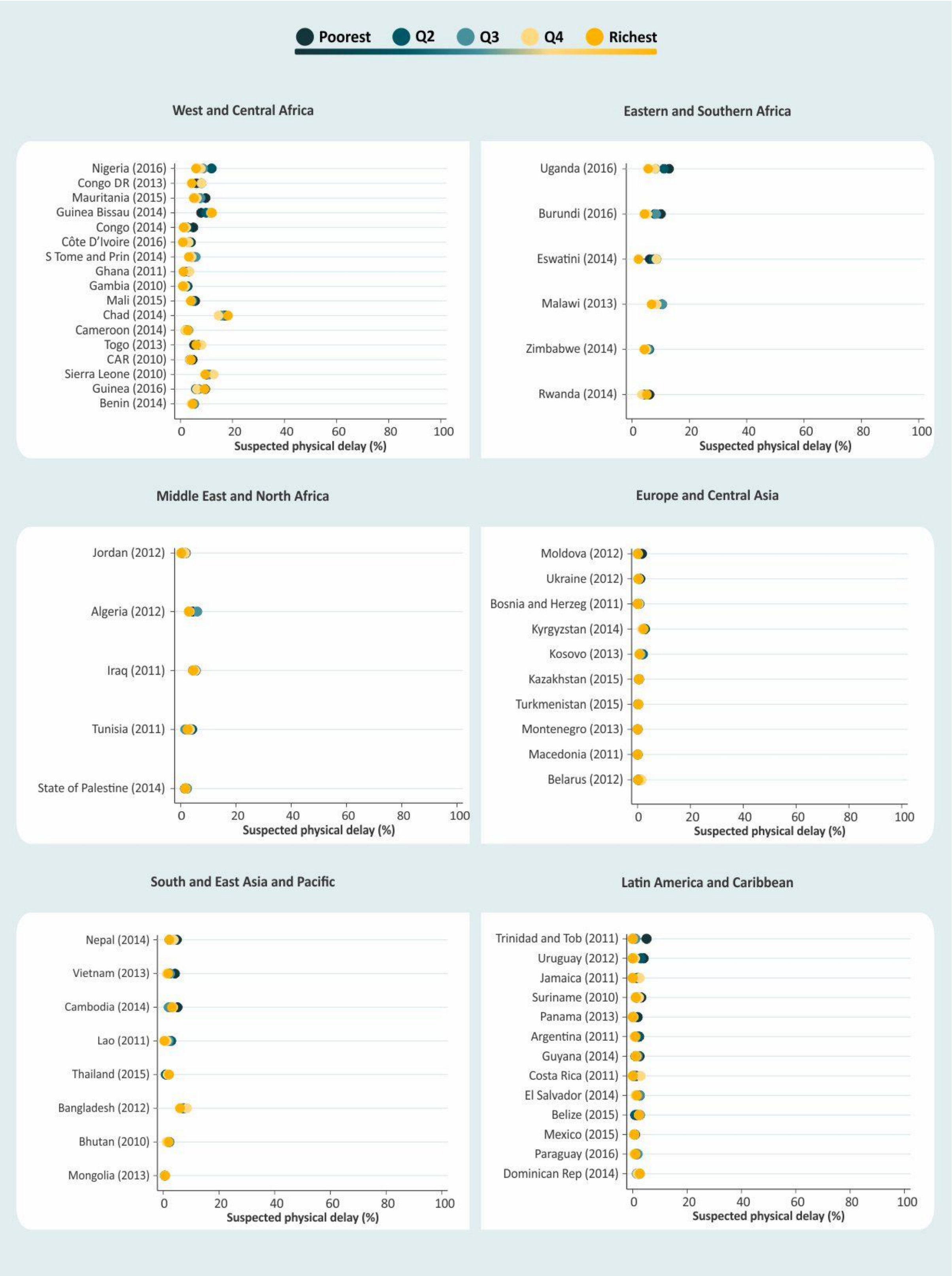

Fig S2. Prevalence of suspected developmental delay (physical domain) by wealth quintiles, countries grouped by world region. Source: MICS and DHS surveys, 2010-2016.

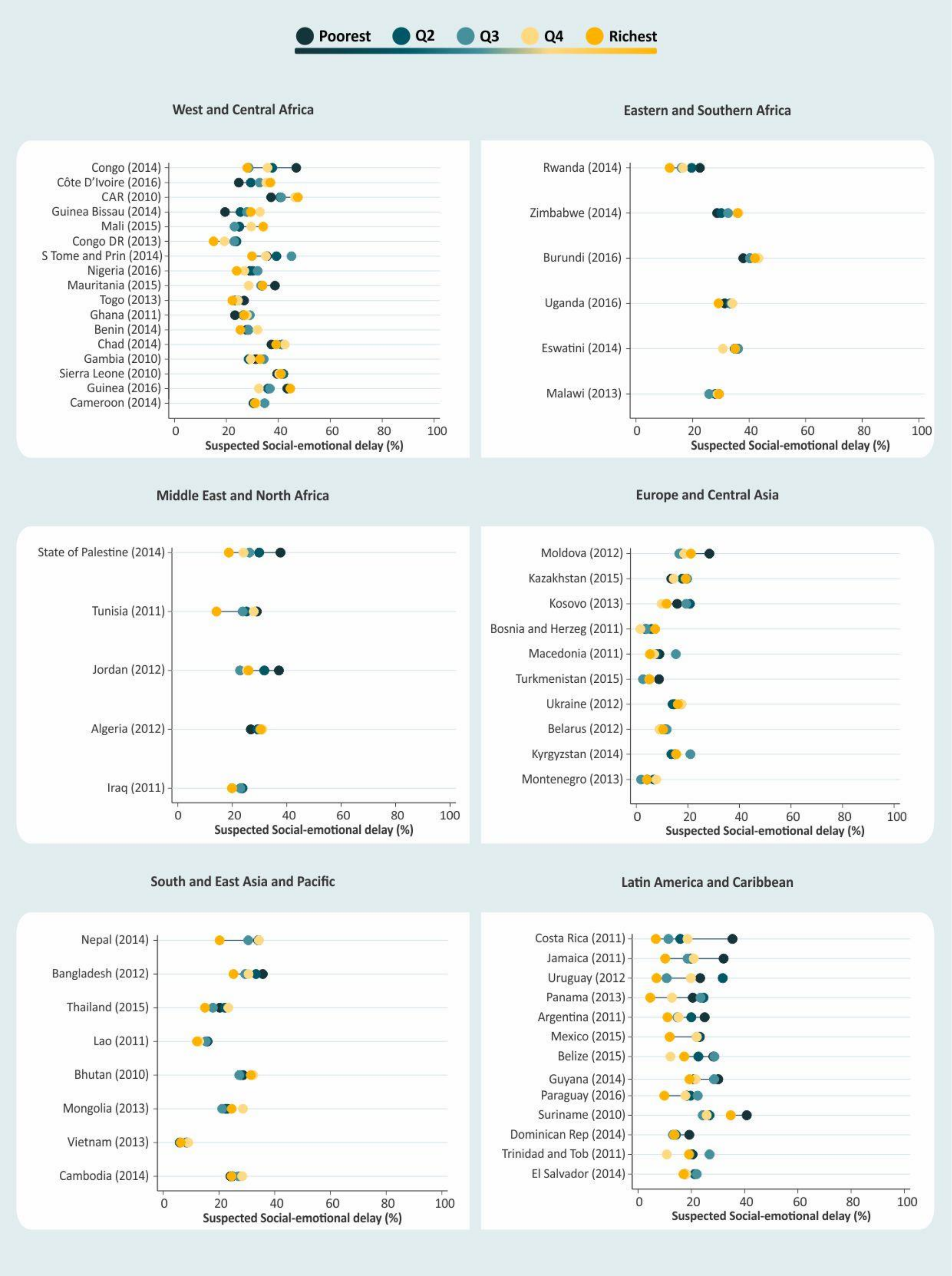

Fig S3. Prevalence of suspected developmental delay (social-emotional domain) by wealth quintiles, countries grouped by world region. Source: MICS and DHS surveys, 2010-2016.

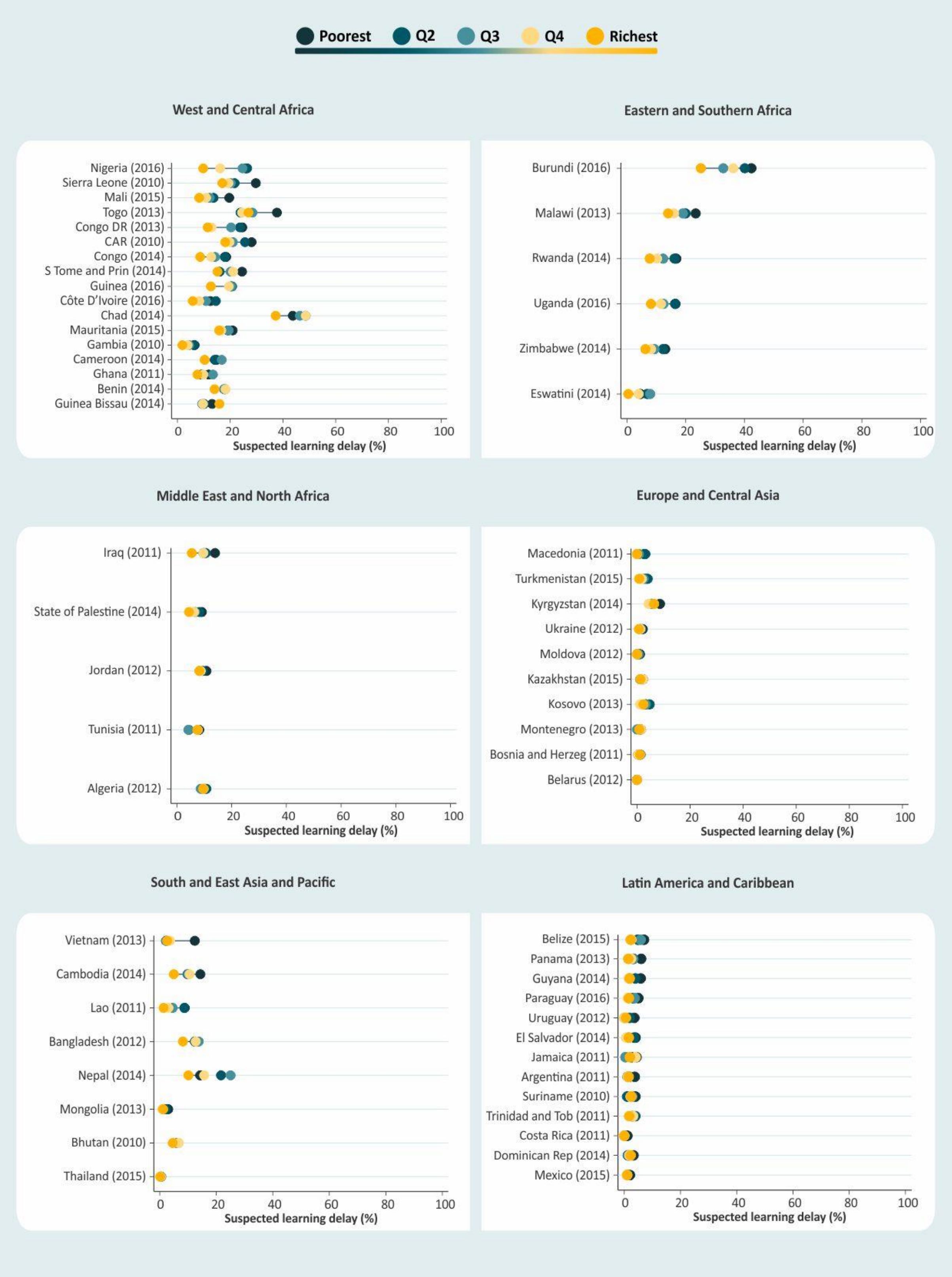

Fig S4. Prevalence of suspected developmental delay (learning domain) by wealth quintiles, countries grouped by world region. Source: MICS and DHS surveys, 2010-2016.

Table S1. Proportions of children with suspected developmental delay in each studied country for the combined indicator (ECDI), sample size and each of the domains; countries grouped by world region. Source: MICS and DHS surveys, 2010-2016.

	Children with suspected developmental delay (%)											
**Country**	**Year**	**N**	**Physical**	**IC95%**	**Social-emotional**	**IC95%**	**Learning**	**IC95%**	**Literacy-numeracy**	**IC95%**	**ECDI**	**IC95%**
**West & Central Africa**												
**Benin**	2014	4882	4.9	[3.7; 6.0]	28.5	[26.5; 30.5]	17.3	[15.6; 18.9]	92.4	[91.2; 93.7]	37.8	[35.8; 39.8]
**Cameroon**	2014	2833	2.4	[1.5; 3.3]	31.6	[29.5; 33.8]	13.6	[11.7; 15.5]	83.9	[81.5; 86.2]	36.7	[34.3; 39.0]
**CAR**	2010	3820	3.9	[3.0; 4.8]	42.4	[39.4; 45.3]	22.7	[20.5; 24.9]	92.7	[91.1; 94.2]	51.7	[49.0; 54.4]
**Chad**	2014	4098	16.5	[14.8; 18.1]	40.4	[37.8; 43.0]	45.4	[42.5; 48.3]	94.6	[93.5; 95.6]	67.3	[64.7; 70.0]
**Congo Brazzaville**	2014	3715	2.8	[2.0; 3.5]	36.1	[33.3; 38.9]	14.9	[12.8; 16.9]	86.3	[84.3; 88.3]	39.1	[36.0; 42.1]
**Congo DR**	2013	2807	7.0	[5.6; 8.5]	21.2	[18.0; 24.5]	19.2	[16.3; 22.0]	89.2	[87.1; 91.3]	34.0	[30.2; 37.8]
**Côte d'lvoire**	2016	3722	3.2	[2.2; 4.1]	31.0	[28.5; 33.5]	11.0	[8.7; 13.2]	92.5	[90.7; 94.3]	36.2	[33.4; 39.1]
**Gambia**	2010	4036	2.1	[1.4; 2.7]	31.3	[28.7; 33.9]	4.7	[3.6; 5.8]	87.7	[85.4; 89.9]	30.4	[27.8; 32.9]
**Ghana**	2011	3002	2.6	[1.8; 3.4]	26.6	[24.0; 29.1]	10.6	[8.6; 12.5]	71.6	[68.6; 74.6]	24.9	[22.1; 27.6]
**Guinea**	2016	3146	7.5	[6.1; 9.0]	38.5	[36.1; 40.8]	19.0	[16.7; 21.4]	94.2	[92.8; 95.5]	50.5	[47.8; 53.3]
**Guinea Bissau**	2014	2955	10.5	[8.1; 12.8]	26.5	[23.8; 29.3]	11.5	[9.8; 13.1]	92.4	[90.6; 94.3]	37.7	[34.5; 41.0]
**Mali**	2015	6548	4.6	[3.8; 5.4]	27.1	[25.4; 28.9]	13.1	[11.6; 14.5]	91.4	[90.2; 92.7]	37.1	[35.0; 39.1]
**Mauritania**	2015	4512	7.5	[6.2; 8.7]	33.9	[31.4; 36.4]	18.6	[16.4; 20.8]	72.5	[70.2; 74.9]	38.4	[36.1; 40.8]
**Nigeria**	2016	11673	9.5	[8.7; 10.4]	28.6	[27.2; 30.0]	21.2	[19.7; 22.6]	70.3	[68.4; 72.2]	37.9	[36.1; 39.6]
**São Tome e Príncipe**	2014	863	4.7	[2.7; 6.8]	37.5	[33.6; 41.4]	19.7	[16.4; 23.1]	84.3	[80.9; 87.8]	45.0	[40.6; 49.4]
**Sierra Leone**	2010	3636	10.7	[9.2; 12.2]	40.8	[37.9; 43.7]	22.2	[20.2; 24.3]	90.5	[89.1; 92.0]	54.0	[51.2; 56.8]
**Togo**	2013	2200	6.6	[5.1; 8.2]	24.3	[22.0; 26.7]	28.6	[25.7; 31.4]	92.3	[90.6; 93.9]	45.2	[42.1; 48.2]
**Eastern & South Africa**												
**Burundi**	2016	4707	7.4	[6.5; 8.4]	40.6	[38.3; 42.9]	35.9	[33.7; 38.1]	91.3	[90.2; 92.4]	59.2	[57.2; 61.3]
**Eswatini**	2014	1055	6.7	[4.9; 8.4]	34.8	[31.1; 38.4]	4.9	[3.1; 6.8]	82.7	[79.8; 85.5]	33.6	[30.2; 37.1]
**Malawi**	2013	7763	9.5	[8.4; 10.7]	28.2	[26.6; 29.8]	18.8	[17.3; 20.3]	82.8	[81.3; 84.2]	39.1	[37.4; 40.9]
**Rwanda**	2014	2673	4.9	[3.9; 5.9]	17.9	[15.5; 20.3]	13.2	[11.5; 14.8]	92.9	[91.6; 94.1]	28.4	[25.9; 30.8]
**Uganda**	2016	4770	9.4	[8.1; 10.7]	32.3	[30.5; 34.2]	13.3	[11.8; 14.8]	71.0	[68.9; 73.1]	34.8	[32.7; 36.8]

**Zimbabwe**	2014	4024	5.4	[4.4; 6.4]	32.4	[30.5; 34.3]	10.2	[8.8; 11.5]	91.4	[90.4; 92.5]	36.8	[35.0; 38.7]
**Middle East & North Africa**												
**Algeria**	2012	5403	4.1	[3.3; 4.9]	29.5	[27.6; 31.4]	9.8	[8.6; 10.9]	71.2	[69.0; 73.4]	29.2	[27.4; 31.0]
**Iraq**	2011	13669	5.1	[4.4; 5.7]	22.3	[21.0; 23.5]	10.4	[9.4; 11.3]	82.0	[80.5; 83.6]	27.7	[26.3; 29.1]
**Jordan**	2012	3670	1.1	[0.5; 1.6]	29.0	[25.6; 32.3]	9.2	[7.6; 10.8]	83.6	[80.9; 86.2]	30.4	[27.2; 33.6]
**Qatar**	2012	820	6.1	[3.7; 8.5]	23.7	[19.4; 28.0]	11.1	[7.6; 14.6]	33.3	[27.2; 39.4]	13.4	[9.4; 14.4]
**State of Palestine**	2014	3274	1.9	[0.9; 2.8]	28.2	[26.4; 30.0]	7.1	[6.0; 8.3]	81.2	[79.4; 82.9]	26.5	[24.8; 28.1]
**Tunisia**	2011	1152	3.1	[1.9; 4.3]	24.3	[21.3; 27.4]	6.3	[4.6; 8.1]	67.5	[63.6; 71.5]	22.1	[19.0; 25.1]
**Europe & Central Asia**												
**Belarus**	2012	1349	0.6	[0.1; 1.1]	10.3	[8.2; 12.3]	0.0	[0.0; 0.0]	52.2	[48.3; 56.1]	5.4	[3.9; 7.0]
**Bosnia and Herzegovina**	2011	917	0.3	[0.0; 0.6]	4.6	[2.4; 6.7]	0.9	[0.3; 1.5]	74.5	[70.2; 78.9]	3.3	[1.5; 5.1]
**Kazakhstan**	2015	2322	0.5	[0.0; 1.1]	17.0	[14.4; 19.5]	1.8	[0.9; 2.7]	72.8	[69.9; 75.7]	13.1	[10.5; 15.6]
**Kosovo**	2013	674	1.1	[0.0; 2.7]	15.5	[12.5; 18.5]	3.0	[1.2; 4.9]	82.6	[78.7; 86.5]	15.8	[13.0; 18.7]
**Kyrgyzstan**	2014	1770	2.3	[1.1; 3.4]	16.1	[14.0; 18.2]	6.2	[4.1; 8.2]	86.1	[83.7; 88.5]	19.7	[17.1; 22.3]
**Macedonia**	2011	561	0.0	[0.0; 0.1]	8.8	[6.0; 11.5]	1.4	[0.4; 2.5]	57.1	[51.9; 62.4]	6.9	[4.4; 9.5]
**Moldova**	2012	712	0.4	[0.0; 1.1]	20.4	[16.9; 23.9]	0.3	[0.0; 1.0]	70.7	[66.9; 74.6]	15.0	[11.9; 18.2]
**Montenegro**	2013	659	0.0	[0.0; 0.0]	5.1	[2.7; 7.4]	1.0	[0.0; 2.4]	78.8	[73.9; 83.7]	4.4	[2.3; 6.5]
**Turkmenistan**	2015	1517	0.3	[0.0; 0.9]	5.4	[3.9; 6.8]	2.6	[1.0; 4.3]	82.7	[79.5; 85.9]	7.7	[6.0; 9.4]
**Ukraine**	2012	1928	0.5	[0.0; 1.1]	16.0	[13.3; 18.7]	1.3	[0.6; 2.0]	54.3	[49.7; 58.9]	9.3	[7.3; 11.3]
**South Asia**												
**Bangladesh**	2012	8638	7.3	[6.3; 8.2]	31.3	[30.0; 32.7]	12.0	[11.0; 13.1]	79.6	[78.2; 80.9]	35.4	[33.9; 36.8]
**Bhutan**	2010	2497	1.8	[1.1; 2.5]	29.6	[27.1; 32.2]	6.1	[4.7; 7.4]	75.0	[72.2; 77.8]	27.4	[24.7; 30]
**Nepal**	2014	2284	3.5	[2.5; 4.6]	31.4	[28.7; 34.0]	17.8	[14.6; 21.1]	71.7	[68.0; 75.4]	35.1	[31.4; 38.8]
**East Asia & Pacific**												
**Cambodia**	2014	2621	3.5	[2.5; 4.4]	25.6	[23.4; 27.8]	10.2	[8.7; 11.7]	72.9	[70.1; 75.7]	26.6	[24.3; 29.0]
**Lao**	2011	4425	1.8	[1.3; 2.3]	14.7	[13.3; 16.2]	6.1	[4.8; 7.3]	80.1	[78.1; 82.2]	18.4	[16.8; 20.1]
**Mongolia**	2013	2337	0.6	[0.0; 1.1]	23.8	[21.8; 25.8]	1.7	[1.0; 2.4]	91.5	[90.1; 92.9]	23.2	[21.2; 25.1]
**Thailand**	2015	5078	1.5	[0.1; 2.8]	20.0	[17.4; 22.6]	0.4	[0.0; 0.9]	28.0	[25.1; 31.0]	6.7	[5.3; 8.2]

**Vietnam**	2013	1197	2.4	[1.1; 3.8]	7.6	[5.5; 9.8]	4.8	[3.1; 6.4]	71.9	[68.5; 75.4]	10.0	[7.8; 12.2]
**Latin America and the Caribbean**												
**Argentina**	2011	1239	1.5	[0.8; 2.3]	18.2	[16.2; 20.3]	2.1	[1.2; 3.0]	58.4	[55.5; 61.3]	13.2	[11.4; 15.0]
**Barbados**	2012	202	0.0	-	19.6	[14.1; 25.0]	0.2	[0.0; 0.6]	8.7	[4.5; 13.0]	3.2	[0.4; 5.9]
**Belize**	2015	1062	1.8	[0.2; 3.5]	22.6	[19.3; 25.8]	4.8	[2.4; 7.2]	46.2	[41.2; 51.1]	15.6	[12.7; 18.6]
**Costa Rica**	2011	883	1.1	[0.2; 1.9]	20.4	[16.0; 24.9]	0.4	[0.0; 0.8]	74.3	[68.2; 80.5]	18.2	[13.7; 22.6]
**Cuba**	2014	2172	0.2	[0.0; 0.9]	10.8	[8.0; 13.5]	1.8	[0.0; 4.7]	79.2	[72.6; 85.7]	9.9	[7.0; 12.9]
**Dominican Republic**	2014	8058	2.0	[1.3; 2.7]	14.9	[13.6; 16.2]	2.0	[1.4; 2.7]	79.4	[77.8; 81.0]	14.5	[13.2; 15.8]
**El Salvador**	2014	2995	2.1	[1.3; 2.8]	19.3	[17.4; 21.1]	2.4	[1.3; 3.4]	81.6	[79.3; 83.9]	18.2	[16.4; 19.9]
**Guyana**	2014	1336	1.7	[0.7; 2.6]	25.0	[22.1; 27.9]	3.5	[2.0; 5.0]	33.5	[30.1; 36.9]	11.4	[9.1; 13.7]
**Jamaica**	2011	662	1.3	[0.2; 2.5]	21.3	[17.0; 25.5]	2.7	[1.3; 4.2]	33.5	[27.9; 39.1]	10	[7.3; 12.7]
**Mexico**	2015	3414	0.6	[0.0; 1.3]	21.1	[18.2; 23.9]	1.3	[0.7; 1.9]	78.2	[74.7; 81.7]	17.3	[14.7; 19.9]
**Panama**	2013	2315	0.7	[0.2; 1.1]	18.6	[15.1; 22.0]	3.7	[2.3; 5.1]	81.2	[77.6; 84.7]	18.9	[15.6; 22.3]
**Paraguay**	2016	1845	1.2	[0.3; 2.1]	17.9	[15.5; 20.3]	3.1	[1.6; 4.5]	77.2	[74.2; 80.2]	17.2	[14.7; 19.7]
**St Lucia**	2012	123	1.1	[0.0; 3.2]	12.8	[6.4; 19.2]	1.2	[0.0; 3.0]	23.4	[13.4; 33.3]	6.9	[1.7; 12.1]
**Suriname**	2010	1278	2.1	[1.3; 3.0]	32.6	[29.1; 36.0]	2.8	[1.7; 3.9]	79.1	[75.8; 82.5]	28.7	[25.4; 32.0]
**Trinidad and Tobago**	2011	523	1.6	[0.4; 2.8]	20.1	[15.8; 24.4]	3.0	[1.1; 4.8]	17.2	[12.9; 21.5]	6	[3.2; 8.8]
**Uruguay**	2012	652	2.1	[0.0; 4.3]	19.8	[12.0; 27.6]	1.8	[0.0; 3.8]	50.1	[40.8; 59.4]	12.7	[6.0; 19.4]

Table S2. Prevalence of suspected developmental delay in each studied country for the physical domain; countries grouped by world region. Source: MICS and DHS surveys, 2010-2016.

	Wealth quintile (%)										Measures of inequality			
**Country**	**Poorest**	**IC95%**	**Q2**	**IC95%**	**Q3**	**IC95%**	**Q4**	**IC95%**	**Richest**	**IC95%**	**SII[1]**	**IC95%**	**CIX[2]**	**IC95%**
**West & Central Africa**														
**Benin**	4.8	[3.1; 6.5]	5.3	[3.3; 7.3]	5.3	[3.0; 7.5]	4.3	[2.8; 5.7]	4.7	[1.9; 7.5]	-0.8	[-1.0; -0.5]	-4.2	[-4.8; -3.5]
**Cameroon**	1.9	[0.9; 3.0]	2.5	[1.3; 3.8]	3.1	[1.2; 4.9]	1.9	[0.9; 2.9]	2.9	[1.3; 4.5]	0.7	[0.4; 1.1]	5.6	[4.0; 7.1]
**CAR**	4.7	[2.5; 6.9]	3.6	[2.3; 5.0]	3.6	[2.0; 5.1]	3.7	[2.0; 5.3]	4.1	[1.3; 6.8]	-1.0	[-1.1; -0.9]	0.9	[0.4; 1.3]
**Chad**	16.8	[13.5; 20.1]	17.4	[14.2; 20.6]	15.8	[12.5; 19.2]	14.6	[11.9; 17.3]	18.3	[15.0; 21.6]	-0.4	[-0.5; -0.2]	1.1	[1.0; 1.1]
**Congo Brazzaville**	5.0	[3.4; 6.5]	2.7	[1.5; 3.8]	2.1	[1.0; 3.3]	2.2	[0.7; 3.7]	1.2	[0.0; 2.4]	-7.2	[-7.7; -6.7]	-20.9	[-22.1; -19.7]
**Congo DR**	6.2	[3.9; 8.4]	8.1	[5.1; 11.1]	8.0	[4.6; 11.5]	8.1	[4.7; 11.5]	4.3	[2.2; 6.5]	-4.1	[-4.3; -3.8]	-1.8	[-1.9; -1.8]
**Côte D'Ivoire**	4.1	[2.3; 5.8]	3.5	[1.9; 5.1]	3.1	[1.5; 4.7]	3.2	[1.5; 4.8]	0.9	[0.0; 1.9]	-3.7	[-4.2; -3.3]	-14.1	[-15.4; -12.9]
**Gambia**	2.7	[1.4; 4.0]	2.7	[1.2; 4.2]	1.8	[0.9; 2.8]	1.9	[0.9; 2.9]	0.9	[0.1; 1.7]	-2.6	[-2.7; -2.5]	-17.6	[-18.2; -16.9]
**Ghana**	3.2	[1.9; 4.5]	1.5	[0.2; 2.8]	3.4	[1.4; 5.4]	3.5	[1.0; 6.1]	1.1	[0.0; 2.5]	-1.3	[-1.4; -1.1]	-9.8	[-10.2; -9.4]
**Guinea**	9.5	[6.6; 12.4]	5.9	[3.8; 8.1]	7.0	[4.5; 9.5]	6.3	[3.8; 8.8]	9.3	[4.3; 14.3]	-1.2	[-1.4; -1.0]	1.7	[1.5; 2.0]
**Guinea Bissau**	7.9	[5.3; 10.5]	9.8	[7.4; 12.3]	11.5	[7.4; 15.6]	11.9	[7.4; 16.3]	12.1	[6.9; 17.4]	5.8	[5.7; 6.0]	3.5	[3.2; 3.7]
**Mali**	5.7	[4.1; 7.2]	4.7	[3.0; 6.3]	4.5	[3.3; 5.7]	4.0	[2.8; 5.3]	4.0	[2.9; 5.0]	-2.8	[-3.0; -2.6]	-7.0	[-7.5; -6.4]
**Mauritania**	9.7	[7.0; 12.4]	7.6	[5.4; 9.8]	7.7	[5.6; 9.8]	6.3	[4.0; 8.6]	5.1	[3.3; 7.0]	-5.9	[-6.2; -5.6]	-11.8	[-12.4; -11.3]
**Nigeria**	11.9	[10.2; 13.5]	12.1	[10.4; 13.7]	8.7	[7.1; 10.3]	7.9	[6.4; 9.4]	6.0	[4.7; 7.3]	-8.5	[-8.7; -8.3]	-13.5	[-13.7; -13.2]
**S Tome and Principe**	5.3	[1.8; 8.9]	4.2	[1.6; 6.9]	5.9	[1.8; 10.0]	4.3	[1.0; 7.7]	3.2	[0.0; 6.6]	-1.5	[-2.4; -0.6]	-7.7	[-10.1; -5.3]
**Sierra Leone**	10.0	[7.3; 12.8]	10.0	[7.3; 12.8]	11.0	[8.2; 13.9]	12.8	[9.4; 16.2]	9.5	[6.5; 12.6]	1.5	[1.2; 1.7]	0.1	[-0.2; 0.5]
**Togo**	5.2	[3.2; 7.2]	7.0	[4.1; 9.9]	7.0	[4.0; 9.9]	8.1	[4.4; 11.8]	6.0	[3.1; 9.0]	2.2	[1.8; 2.6]	1.8	[0.9; 2.6]
**Eastern & Southern Africa**														
**Burundi**	10.1	[8.0; 12.2]	7.9	[5.7; 10.1]	8.5	[6.6; 10.3]	5.4	[3.7; 7.0]	4.3	[2.7; 6.0]	-7.3	[-7.4; -7.1]	-15.1	[-15.4; -14.7]
**Eswatini**	6.2	[3.3; 9.0]	7.5	[4.2; 10.8]	8.6	[4.1; 13.0]	8.5	[3.7; 13.3]	2.2	[0.0; 4.9]	-0.7	[-1.6; 0.1]	-5.3	[-6.8; -3.9]
**Malawi**	10.3	[8.4; 12.3]	10.5	[8.5; 12.6]	10.5	[8.3; 12.7]	8.6	[6.6; 10.6]	6.8	[0.4; 9.6]	-4.7	[-5.0; -4.3]	-6.7	[-7.2; -6.1]
**Rwanda**	6.1	[4.2; 8.1]	4.2	[2.6; 5.8]	5.2	[3.2; 7.1]	3.4	[1.6; 5.2]	5.2	[2.5; 7.9]	-1.6	[-1.7; -1.5]	0.3	[0.2; 0.5]
**Uganda**	12.9	[10.3; 15.6]	11.2	[8.9; 13.5]	8.1	[5.7; 10.6]	8.0	[5.4; 10.5]	5.6	[3.4; 7.8]	-9.1	[-9.2; -9.1]	-14.1	[-14.2; -13.9]
**Zimbabwe**	5.5	[3.9; 7.2]	6.1	[4.3; 7.8]	5.6	[3.8; 7.4]	5.0	[3.5; 6.5]	4.3	[2.7; 5.8]	-2.1	[-2.7; -1.6]	-4.6	[-5.8; -3.5]
**Middle East & North Africa**														
**Algeria**	4.3	[2.7; 5.9]	3.7	[2.4; 5.1]	6.0	[3.9; 8.1]	3.2	[1.9; 4.6]	3.0	[1.5; 4.5]	-1.4	[-1.4; -1.3]	-9.6	[-9.8; -9.4]
**Iraq**	5.4	[4.3; 6.5]	5.4	[4.2; 6.7]	4.5	[3.3; 5.6]	5.2	[3.8; 6.7]	4.5	[2.5; 6.6]	-1.1	[-1.1; -1.0]	-4.9	[-5.1; -4.7]
**Jordan**	1.8	[0.0; 3.7]	0.6	[0.0; 1.1]	0.8	[0.0; 1.8]	1.6	[0.6; 2.6]	0.2	[0.0; 0.4]	-1.1	[-1.2; -1.0]	-10.9	[-11.7; -10.2]
**State of Palestine**	1.6	[0.6; 2.5]	2.0	[0.8; 3.1]	2.3	[0.9; 3.8]	1.8	[0.7; 2.9]	1.7	[0.6; 2.8]	0.0	[-0.5; 0.4]	-2.0	[-4.0; 0.1]
**Tunisia**	3.4	[1.4; 5.3]	4.2	[1.6; 6.8]	1.7	[0.0; 3.7]	3.4	[0.7; 6.1]	2.5	[0.0; 5.1]	-1.3	[-1.5; -1.0]	-4.5	[-5.5; -3.5]
**Europe & Central Asia**														
**Belarus**	0.2	[0.0; 0.6]	0.3	[0.0; 0.6]	1.2	[0.0; 2.8]	1.5	[0.0; 3.1]	0.2	[0.0; 0.5]	0.3	[0.2; 0.4]	13.5	[12.3; 14.8]

**Bosnia and Herzegovina**	0.4	[0.0; 1.2]	0.0	-	0.7	[0.0; 1.9]	0.3	[0.0; 1.0]	0.0	-	-0.4	[-0.4; -0.3]	-9.0	[-10.8; -7.2]
**Kazakhstan**	0.6	[0.0; 1.3]	0.5	[0.0; 1.1]	0.4	[0.0; 1.0]	0.5	[0.0; 1.2]	0.5	[0.0; 1.2]	0.0	[-0.3; 0.3]	-1.1	[-4.3; 2.0]
**Kosovo**	0.8	[0.0; 2.3]	2.0	[0.0; 5.2]	1.3	[0.0; 3.4]	0.8	[0.0; 2.4]	0.7	[0.0; 1.9]	-1.2	[-2.0; -0.4]	-1.4	[-6.9; 4.0]
**Kyrgyzstan**	2.5	[0.9; 4.2]	2.8	[0.9; 4.7]	2.0	[0.7; 3.4]	1.7	[0.4; 3.0]	2.2	[0.5; 4.0]	-1.6	[-2.4; -0.9]	-4.9	[-7.4; -2.3]
**Macedonia**	0.0	-	0.0	-	0.0	-	0.0	-	0.0	-	0.0	[-0.3; 0.2]	5.4	[-6.1; 16.9]
**Moldova**	1.6	[0.0; 3.8]	0.4	[0.0; 1.6]	0.0	-	0.0	-	0.1	[0.0; 0.5]	-2.8	[-3.5; -2.1]	-74.3	[-80.6; -68.0]
**Montenegro**	0.0	[0.0; 0.0]	0.0	[0.0; 0.0]	0.0	-	0.0	[0.0; 0.0]	0.0	[0.0; 0.0]	0.9	[-0.8; 2.5]	-6.7	[-28.2; 14.7]
**Turkmenistan**	0.3	[0.0; 1.0]	0.2	[0.0; 0.6]	0.2	[0.0; 0.8]	0.3	[0.0; 1.2]	0.2	[0.0; 0.9]	0.5	[-0.2; 1.2]	2.2	[-2.5; 6.9]
**Ukraine**	1.0	[0.0; 2.4]	0.4	[0.0; 1.2]	0.4	[0.0; 1.2]	0.3	[0.0; 0.9]	0.2	[0.0; 0.6]	-3.3	[-3.9; -2.6]	-23.8	[-27.2; -20.4]
**South Asia**														
**Bangladesh**	7.1	[5.4; 8.7]	7.3	[5.7; 8.9]	7.6	[5.8; 9.4]	8.5	[6.5; 10.6]	6.0	[4.2; 7.8]	0.4	[0.2; 0.5]	-2.6	[-2.9; -2.2]
**Bhutan**	2.2	[0.5; 4.0]	1.5	[0.4; 2.6]	2.2	[0.5; 3.9]	1.2	[0.0; 2.3]	1.9	[0.2; 3.6]	-0.6	[-0.7; -0.6]	-7.5	[-7.6; -7.5]
**Nepal**	4.9	[3.3; 6.4]	2.2	[0.6; 3.9]	3.9	[1.7; 6.1]	3.8	[1.1; 6.4]	2.2	[0.0; 4.7]	-2.8	[-3.0; -2.7]	-6.5	[-7.0; -6.0]
**East Asia & the Pacific**														
**Cambodia**	5.2	[2.8; 7.5]	3.1	[1.4; 4.9]	1.9	[0.0; 3.1]	3.6	[1.4; 5.8]	3.1	[1.2; 5.0]	-2.4	[-2.5; -2.2]	-8.6	[-9.0; -8.3]
**Lao**	1.9	[1.1; 2.7]	2.9	[1.6; 4.2]	2.0	[1.0; 3.0]	1.3	[0.3; 2.2]	0.4	[0.0; 0.9]	-1.7	[-1.8; -1.5]	-21.2	[-21.9; -20.6]
**Mongolia**	0.6	[0.0; 1.4]	0.5	[0.0; 1.1]	0.5	[0.0; 1.1]	0.5	[0.0; 1.1]	0.7	[0.0; 1.6]	0.2	[0.0; 0.3]	4.6	[2.4; 6.7]
**Thailand**	1.1	[0.1; 2.0]	0.9	[0.0; 1.6]	2.0	[0.3; 3.6]	1.6	[0.3; 2.9]	2.1	[0.0; 5.7]	2.1	[1.9; 2.3]	16.7	[15.6; 17.9]
**Vietnam**	4.2	[1.1; 7.4]	2.3	[0.3; 4.2]	2.5	[0.3; 4.6]	1.2	[0.0; 2.7]	1.8	[0.1; 3.4]	-5.2	[-6.0; -4.4]	-26.4	[-29.0; -23.7]
**Latin America & Caribbean**														
**Argentina**	2.3	[0.8; 3.9]	2.0	[0.1; 4.0]	1.1	[0.3; 2.0]	0.7	[0.0; 2.0]	0.9	[0.1; 1.7]	-2.3	[-2.4; -2.3]	-22.0	[-22.4; -21.7]
**Belize**	1.6	[0.0; 3.3]	0.9	[0.0; 2.0]	2.7	[0.6; 4.7]	2.3	[0.0; 5.6]	2.3	[0.0; 5.0]	2.5	[1.8; 3.2]	11.8	[8.7; 14.9]
**Costa Rica**	1.3	[0.0; 3.4]	0.2	[0.0; 0.6]	0.5	[0.0; 1.4]	2.9	[0.0; 6.7]	0.0	-	-1.0	[-1.8; -0.2]	-9.5	[-12.8; -6.1]
**Dominican Republic**	2.4	[1.3; 3.5]	1.9	[1.0; 2.9]	1.5	[0.7; 2.3]	1.8	[0.9; 2.7]	2.6	[1.0; 4.2]	-0.7	[-1.2; -0.2]	-1.7	[-3.2; -0.1]
**El Salvador**	2.5	[1.2; 3.8]	2.5	[1.4; 3.7]	2.4	[1.2; 3.7]	1.0	[0.3; 1.7]	1.5	[0.5; 2.6]	-2.9	[-3.4; -2.5]	-13.9	[-15.9; -12.0]
**Guyana**	2.5	[0.9; 4.0]	1.0	[0.0; 2.1]	1.8	[0.0; 3.6]	1.3	[0.0; 2.7]	1.1	[0.0; 2.3]	-3.3	[-3.9; -2.6]	-28.2	[-31.4; -25.0]
**Jamaica**	2.1	[0.0; 5.5]	1.6	[0.0; 3.7]	0.0	-	2.5	[0.2; 4.9]	0.0	-	-2.0	[-2.5; -1.5]	-17.4	[-20.0; -14.8]
**Mexico**	0.8	[0.0; 1.7]	0.6	[0.0; 1.5]	0.6	[0.0; 1.3]	0.5	[0.0; 1.1]	0.5	[0.0; 1.3]	-1.6	[-2.0; -1.3]	-6.7	[-9.4; -4.0]
**Panama**	1.8	[0.3; 3.2]	0.4	[0.0; 0.8]	0.1	[0.0; 0.2]	0.3	[0.0; 0.8]	0.0	-	-6.1	[-6.4; -5.9]	-50.3	[-51.7; -48.9]
**Paraguay**	1.2	[0.0; 2.5]	1.4	[0.0; 2.9]	1.8	[0.4; 3.2]	0.6	[0.0; 1.3]	1.0	[0.1; 1.9]	-1.1	[-1.5; -0.7]	-7.4	[-10.3; -4.6]
**Suriname**	3.1	[1.7; 4.6]	1.5	[0.3; 2.7]	1.2	[0.0; 3.2]	2.3	[0.0; 5.6]	1.1	[0.0; 3.2]	-3.4	[-3.5; -3.2]	-19.9	[-20.4; -19.4]
**Trinidad and Tobago**	5.1	[1.1; 9.1]	0.0	-	0.9	[0.0; 2.9]	0.0	-	0.0	-	-10.9	[-11.6; -10.1]	-50.5	[-55.2; -45.9]
**Uruguay**	4.0	[0.0; 9.6]	3.0	[0.0; 7.4]	0.0	-	0.8	[0.0; 2.4]	0.0	-	-8.7	[-9.2; -8.2]	-29.1	[-30.7; -27.5]

Note: [1] Slope Index of Inequality; [2] Concentration Index

Table S3. Prevalence of suspected developmental delay in each studied country for the social-emotional domain; countries grouped by world region. Source: MICS and DHS surveys, 2010-2016.

	Wealth quintile (%)										Measures of inequality			
**Country**	**Poorest**	**IC95%**	**Q2**	**IC95%**	**Q3**	**IC95%**	**Q4**	**IC95%**	**Richest**	**IC95%**	**SII[1]**	**IC95%**	**CIX[2]**	**IC95%**
**West & Central Africa**														
**Benin**	27.8	[24.0; 31.5]	28.5	[24.7; 32.3]	28.5	[24.3; 32.7]	32.0	[27.8; 36.1]	25.4	[21.6; 29.1]	-1.7	[-2.0; -1.4]	-1.4	[-1.6; -1.3]
**Cameroon**	30.7	[26.1; 35.3]	30.4	[25.7; 35.1]	34.7	[30.3; 39.2]	31.3	[26.4; 36.2]	31.0	[25.8; 36.2]	1.1	[0.9; 1.3]	0.2	[0.1; 0.3]
**CAR**	37.2	[32.4; 42.0]	40.7	[36.8; 44.7]	41.1	[36.0; 46.2]	46.4	[40.6; 52.1]	47.6	[40.7; 54.4]	11.9	[11.8; 12.1]	3.0	[2.9; 3.1]
**Chad**	37.3	[33.1; 41.6]	41.2	[37.2; 45.2]	41.5	[36.9; 46.2]	42.5	[37.0; 48.0]	39.1	[33.8; 44.4]	3.0	[2.8; 3.3]	-0.4	[-0.5; -0.4]
**Congo Brazzaville**	46.9	[43.6; 50.2]	37.7	[32.6; 42.8]	28.6	[22.8; 34.4]	35.9	[29.6; 42.1]	28.0	[21.8; 34.2]	-24.9	[-25.4; -24.4]	-8.7	[-8.8; -8.5]
**Congo DR**	23.4	[17.6; 29.2]	23.9	[18.3; 29.4]	23.0	[17.7; 28.3]	19.2	[13.5; 25.0]	15.0	[10.5; 19.5]	-7.7	[-7.9; -7.4]	-2.3	[-2.4; -2.2]
**Côte D'Ivoire**	24.8	[21.3; 28.3]	29.4	[24.8; 34.0]	32.8	[28.3; 37.3]	35.3	[28.7; 41.9]	37.0	[30.0; 43.9]	15.0	[14.9; 15.1]	6.6	[6.5; 6.7]
**Gambia**	31.3	[27.4; 35.2]	28.5	[24.6; 32.5]	34.4	[29.4; 39.4]	29.3	[24.4; 34.3]	32.9	[27.6; 38.3]	1.3	[1.1; 1.6]	0.7	[0.6; 0.8]
**Ghana**	23.3	[19.5; 27.1]	26.5	[21.8; 31.2]	29.0	[22.7; 35.4]	27.9	[21.6; 34.1]	26.8	[19.4; 34.1]	7.3	[6.9; 7.7]	1.3	[1.0; 1.5]
**Guinea**	43.4	[38.3; 48.5]	36.0	[31.9; 40.1]	36.7	[31.7; 41.8]	32.5	[28.1; 36.9]	44.6	[38.7; 50.5]	-3.8	[-4.5; -3.2]	0.6	[0.6; 0.7]
**Guinea Bissau**	19.5	[15.8; 23.2]	25.4	[21.4; 29.4]	27.9	[23.2; 32.6]	32.9	[26.6; 39.3]	29.4	[20.5; 38.2]	15.9	[15.4; 16.3]	4.0	[3.8; 4.3]
**Mali**	25.0	[21.9; 28.1]	24.9	[21.6; 28.3]	23.1	[20.2; 26.0]	29.5	[26.3; 32.8]	34.2	[31.0; 37.3]	11.0	[10.8; 11.1]	7.0	[6.9; 7.1]
**Mauritania**	38.7	[33.6; 43.9]	33.7	[29.7; 37.7]	33.4	[29.1; 37.6]	28.6	[24.1; 33.0]	33.9	[28.3; 39.6]	-7.9	[-8.2; -7.7]	-3.0	[-3.0; -2.9]
**Nigeria**	29.1	[26.5; 31.7]	30.1	[27.4; 32.8]	32.0	[28.5; 35.6]	26.9	[23.8; 30.0]	24.0	[21.6; 26.4]	-6.0	[-6.1; -5.8]	-4.5	[-4.5; -4.5]
**S Tome and Principe**	35.6	[27.8; 43.4]	39.3	[32.2; 46.4]	45.0	[35.7; 54.4]	35.2	[27.5; 42.9]	29.9	[21.3; 38.5]	-2.9	[-4.0; -1.9]	-3.0	[-3.2; -2.9]
**Sierra Leone**	39.5	[34.6; 44.5]	42.0	[37.0; 46.9]	41.1	[35.6; 46.7]	40.3	[35.6; 45.1]	41.0	[35.4; 46.6]	1.1	[0.8; 1.3]	-0.3	[-0.4; -0.3]
**Togo**	26.8	[21.9; 31.8]	23.3	[18.8; 27.7]	24.5	[20.0; 29.1]	24.4	[19.2; 29.5]	22.3	[17.4; 27.2]	-5.0	[-5.2; -4.8]	-2.5	[-2.7; -2.4]
**Eastern & Southern Africa**														
**Burundi**	37.9	[34.1; 41.8]	40.3	[36.6; 44.0]	40.3	[36.4; 44.2]	43.4	[38.9; 47.8]	42.1	[37.8; 46.3]	5.6	[5.4; 5.8]	1.5	[1.4; 1.6]
**Eswatini**	36.0	[29.8; 42.1]	34.9	[28.0; 41.8]	36.1	[29.1; 43.1]	30.8	[21.8; 39.7]	35.1	[25.3; 44.8]	-3.1	[-4.4; -1.7]	-2.4	[-3.0; -1.8]
**Malawi**	29.2	[25.8; 32.7]	28.1	[24.9; 31.3]	25.9	[22.9; 28.8]	28.9	[25.7; 32.0]	29.4	[25.5; 33.3]	0.0	[-0.3; 0.2]	0.7	[0.6; 0.8]
**Rwanda**	22.6	[18.5; 26.7]	19.7	[15.6; 23.8]	16.1	[12.7; 19.5]	16.6	[12.9; 20.3]	11.9	[8.0; 15.8]	-12.6	[-12.8; -12.4]	-11.7	[-12.0; -11.4]
**Uganda**	31.4	[27.7; 35.0]	33.4	[30.1; 36.7]	33.7	[29.9; 37.5]	34.1	[30.0; 38.2]	29.2	[25.4; 32.9]	-0.4	[-0.6; -0.1]	-2.0	[-2.1; -2.0]
**Zimbabwe**	28.7	[25.0; 32.4]	30.2	[26.4; 33.9]	32.6	[28.7; 36.5]	36.3	[32.5; 40.0]	35.9	[31.7; 40.2]	10.4	[10.0; 10.8]	4.9	[4.7; 5.1]
**Middle East & North Africa**														
**Algeria**	26.8	[22.9; 30.8]	29.3	[25.8; 32.8]	30.2	[26.5; 33.9]	31.0	[27.3; 34.8]	30.4	[26.0; 34.8]	4.8	[4.8; 4.9]	2.0	[1.9; 2.0]
**Iraq**	23.3	[21.3; 25.4]	23.8	[21.3; 26.2]	22.9	[20.2; 25.6]	19.8	[17.0; 22.7]	19.9	[16.3; 23.6]	-4.1	[-4.2; -4.0]	-3.5	[-3.6; -3.5]
**Jordan**	37.1	[31.3; 43.0]	31.8	[26.6; 36.9]	22.9	[17.1; 28.6]	25.4	[19.7; 31.2]	26.0	[17.1; 34.9]	-17.6	[-17.9; -17.4]	-6.2	[-6.3; -6.0]
**State of Palestine**	37.7	[33.9; 41.5]	29.9	[26.1; 33.6]	26.3	[22.8; 29.8]	24.1	[20.7; 27.5]	18.7	[15.3; 22.1]	-22.3	[-22.7; -21.8]	-13.1	[-13.3; -12.8]
**Tunisia**	29.0	[23.1; 34.9]	25.2	[19.3; 31.1]	23.8	[16.7; 31.0]	27.9	[20.5; 35.3]	14.2	[8.2; 20.2]	-11.4	[-12.5; -10.3]	-9.0	[-9.4; -8.6]
**Europe & Central Asia**														
**Belarus**	11.2	[4.8; 17.6]	9.2	[4.9; 13.5]	11.8	[6.2; 17.4]	9.0	[4.3; 13.6]	10.4	[7.2; 13.7]	-0.5	[-0.7; -0.3]	-2.5	[-2.7; -2.3]

**Bosnia and Herzegovina**	3.6	[1.0; 6.3]	5.9	[0.0; 12.2]	4.0	[1.5; 6.6]	1.6	[0.0; 3.1]	7.3	[1.3; 13.3]	2.1	[1.7; 2.5]	-6.3	[-6.8; -5.7]
**Kazakhstan**	13.6	[9.3; 17.8]	17.9	[10.8; 24.9]	19.7	[14.7; 24.7]	14.5	[10.3; 18.6]	19.1	[13.8; 24.4]	4.5	[3.8; 5.2]	2.2	[1.6; 2.8]
**Kosovo**	15.9	[10.4; 21.4]	20.8	[13.1; 28.5]	19.4	[12.5; 26.2]	9.8	[4.5; 15.0]	11.7	[5.7; 17.8]	-8.8	[-10.4; -7.1]	-5.4	[-7.0; -3.9]
**Kyrgyzstan**	15.0	[10.8; 19.2]	13.6	[9.7; 17.5]	21.0	[15.9; 26.0]	15.5	[10.4; 20.5]	15.5	[10.3; 20.7]	2.7	[1.8; 3.7]	0.6	[-0.3; 1.4]
**Macedonia**	9.0	[2.0; 16.0]	7.7	[2.9; 12.6]	15.3	[7.5; 23.1]	6.9	[2.1; 11.7]	5.3	[1.2; 9.4]	-4.3	[-4.9; -3.7]	-4.8	[-5.3; -4.2]
**Moldova**	28.3	[19.4; 37.3]	17.3	[10.2; 24.4]	16.6	[9.3; 23.9]	18.6	[11.5; 25.7]	21.2	[15.5; 26.9]	-2.2	[-3.6; -0.7]	-5.6	[-6.2; -5.0]
**Montenegro**	4.2	[0.2; 8.3]	6.8	[1.9; 11.7]	1.9	[0.0; 4.5]	7.8	[0.4; 15.2]	4.1	[0.2; 8.0]	0.4	[-0.4; 1.1]	0.3	[-1.7; 2.3]
**Turkmenistan**	8.8	[5.5; 12.1]	5.0	[2.2; 7.8]	2.6	[0.4; 4.7]	5.0	[2.0; 8.1]	5.1	[2.4; 7.9]	-3.2	[-3.9; -2.6]	-4.6	[-5.8; -3.3]
**Ukraine**	14.8	[9.8; 19.8]	14.1	[9.3; 18.8]	17.5	[11.8; 23.1]	17.6	[12.0; 23.1]	16.1	[10.9; 21.3]	3.0	[2.4; 3.6]	3.1	[2.6; 3.7]
**South Asia**														
**Bangladesh**	35.7	[33.3; 38.1]	33.3	[30.8; 35.8]	29.4	[26.7; 32.2]	30.6	[27.4; 33.8]	25.2	[22.0; 28.4]	-11.6	[-11.9; -11.4]	-5.1	[-5.2; -5.0]
**Bhutan**	28.7	[23.2; 34.2]	27.8	[23.8; 31.8]	27.3	[22.2; 32.4]	32.3	[25.6; 39.0]	31.4	[25.1; 37.8]	4.3	[3.9; 4.7]	1.8	[1.6; 2.0]
**Nepal**	34.1	[29.4; 38.7]	34.2	[27.9; 40.5]	30.5	[25.5; 35.5]	34.3	[28.1; 40.6]	20.2	[14.1; 26.4]	-10.2	[-10.9; -9.5]	-7.0	[-7.2; -6.9]
**East Asia & the Pacific**														
**Cambodia**	24.1	[20.5; 27.6]	24.9	[20.6; 29.2]	27.1	[21.4; 32.9]	28.3	[22.5; 34.1]	24.5	[19.9; 29.1]	1.9	[1.7; 2.1]	-0.4	[-0.4; -0.4]
**Lao**	15.9	[13.2; 18.7]	15.7	[13.2; 18.2]	15.3	[12.7; 17.9]	12.9	[10.1; 15.7]	12.1	[8.9; 15.3]	-4.4	[-4.4; -4.3]	-4.4	[-4.5; -4.4]
**Mongolia**	22.4	[19.1; 25.6]	23.2	[18.8; 27.5]	21.2	[17.0; 25.3]	28.6	[23.5; 33.7]	24.6	[20.1; 29.0]	4.8	[4.3; 5.4]	3.9	[3.5; 4.2]
**Thailand**	20.3	[15.7; 24.9]	22.2	[17.3; 27.0]	17.8	[13.5; 22.1]	23.3	[17.7; 29.0]	15.0	[9.0; 20.9]	-3.1	[-3.5; -2.7]	-4.4	[-4.6; -4.1]
**Vietnam**	8.4	[5.4; 11.3]	5.9	[2.2; 9.6]	8.5	[4.5; 12.5]	9.0	[4.4; 13.6]	6.3	[2.1; 10.5]	-1.4	[-2.4; -0.4]	-4.5	[-6.2; -2.7]
**Latin America & Caribbean**														
**Argentina**	25.0	[20.4; 29.5]	20.0	[14.9; 25.0]	14.8	[10.6; 19.0]	15.2	[10.0; 20.4]	11.0	[7.2; 14.8]	-17.4	[-17.9; -17.0]	-14.5	[-14.9; -14.1]
**Belize**	28.3	[21.5; 35.0]	22.6	[16.2; 29.0]	28.7	[22.4; 34.9]	12.1	[7.5; 16.8]	17.3	[10.5; 24.1]	-16.5	[-17.6; -15.5]	-11.9	[-12.6; -11.2]
**Costa Rica**	35.5	[26.4; 44.5]	15.9	[9.0; 22.8]	11.4	[5.6; 17.1]	18.6	[7.4; 29.8]	6.7	[0.6; 12.7]	-35.6	[-36.7; -34.6]	-23.9	[-24.7; -23.2]
**Dominican Republic**	19.2	[16.5; 21.9]	14.2	[11.9; 16.4]	13.1	[10.5; 15.6]	13.7	[10.2; 17.1]	13.6	[9.6; 17.5]	-8.0	[-8.5; -7.6]	-7.0	[-7.4; -6.5]
**El Salvador**	17.7	[14.6; 20.9]	21.2	[17.5; 24.9]	22.0	[17.6; 26.4]	18.1	[13.7; 22.4]	17.1	[12.4; 21.7]	-1.6	[-2.3; -0.8]	-1.3	[-1.9; -0.7]
**Guyana**	30.1	[25.9; 34.2]	21.1	[14.1; 28.0]	28.5	[21.9; 35.2]	21.6	[14.7; 28.5]	19.3	[12.8; 25.8]	-13.2	[-14.0; -12.3]	-11.5	[-12.1; -10.9]
**Jamaica**	32.1	[22.8; 41.4]	19.9	[12.8; 26.9]	18.6	[10.5; 26.6]	20.9	[12.7; 29.1]	10.2	[1.5; 18.8]	-20.9	[-22.3; -19.5]	-16.7	[-17.6; -15.9]
**Mexico**	23.2	[19.0; 27.3]	22.7	[18.5; 26.9]	22.0	[14.5; 29.6]	22.0	[16.2; 27.8]	11.8	[4.8; 18.9]	-7.7	[-8.4; -7.0]	-6.1	[-6.6; -5.6]
**Panama**	20.5	[16.5; 24.6]	24.6	[15.6; 33.6]	23.5	[15.8; 31.1]	12.7	[5.1; 20.4]	4.6	[0.8; 8.3]	-9.6	[-10.7; -8.6]	-8.7	[-9.0; -8.3]
**Paraguay**	18.3	[14.2; 22.4]	19.7	[14.3; 25.1]	22.4	[16.2; 28.5]	17.8	[12.3; 23.2]	9.8	[5.5; 14.2]	-7.3	[-8.2; -6.4]	-7.7	[-8.4; -7.1]
**Suriname**	40.8	[35.6; 46.0]	26.8	[19.3; 34.3]	24.3	[16.4; 32.2]	25.8	[16.4; 35.1]	34.8	[23.8; 45.8]	-22.8	[-23.9; -21.6]	-10.4	[-10.5; -10.3]
**Trinidad and Tobago**	20.4	[12.7; 28.2]	19.6	[9.6; 29.6]	26.8	[18.2; 35.4]	10.8	[4.1; 17.5]	19.1	[10.0; 28.2]	-3.0	[-5.3; -0.7]	2.6	[1.2; 4.0]
**Uruguay**	23.3	[9.1; 37.6]	31.8	[9.9; 53.8]	10.7	[2.5; 18.8]	19.8	[2.3; 37.3]	6.9	[2.6; 11.3]	-23.3	[-24.5; -22.1]	-18.3	[-18.8; -17.8]

Note: [1] Slope Index of Inequality; [2] Concentration Index

Table S4. Prevalence of suspected developmental delay in each studied country for the learning domain; countries grouped by world region. Source: MICS and DHS surveys, 2010-2016.

	Wealth quintile (%)										Measures of inequality			
**Country**	**Poorest**	**IC95%**	**Q2**	**IC95%**	**Q3**	**IC95%**	**Q4**	**IC95%**	**Richest**	**IC95%**	**SII[1]**	**IC95%**	**CIX[2]**	**IC95%**
							**West & Central Africa**							
**Benin**	18.1	[14.8; 21.4]	17.7	[14.3; 21.0]	17.9	[14.5; 21.2]	18.3	[14.7; 21.8]	14.1	[11.3; 16.8]	-4.9	[-5.5; -4.3]	-4.5	[-5.0; -4.0]
**Cameroon**	14.8	[11.0; 18.7]	14.1	[10.9; 17.3]	16.8	[13.0; 20.7]	10.2	[7.2; 13.1]	10.4	[6.7; 14.1]	-6.9	[-7.9; -5.9]	-7.3	[-8.3; -6.3]
**CAR**	28.1	[23.4; 32.9]	25.6	[21.9; 29.3]	21.0	[17.6; 24.5]	19.8	[15.3; 24.3]	18.1	[13.6; 22.6]	-13.3	[-13.9; -12.7]	-6.7	[-7.1; -6.3]
**Chad**	43.8	[39.1; 48.4]	48.6	[43.9; 53.4]	46.4	[41.9; 51.0]	48.7	[42.3; 55.1]	37.3	[32.3; 42.2]	-5.4	[-6.0; -4.8]	-3.1	[-3.3; -3.0]
**Congo Brazzaville**	18.5	[14.8; 22.2]	17.9	[14.0; 21.9]	14.3	[10.6; 17.9]	12.8	[8.1; 17.5]	8.6	[4.9; 12.3]	-9.7	[-10.3; -9.1]	-11.5	[-12.0; -11.0]
**Congo DR**	24.6	[19.3; 29.9]	23.7	[18.2; 29.2]	20.3	[14.1; 26.6]	12.9	[8.5; 17.4]	11.5	[7.7; 15.3]	-12.0	[-12.2; -11.8]	-2.7	[-2.8; -2.6]
**Côte D'Ivoire**	12.5	[8.7; 16.2]	14.6	[10.8; 18.4]	10.9	[7.9; 13.8]	8.3	[5.7; 10.9]	5.8	[2.8; 8.8]	-8.5	[-9.1; -7.9]	-12.8	[-13.5; -12.1]
**Gambia**	6.5	[4.6; 8.5]	6.2	[3.8; 8.5]	4.4	[2.6; 6.2]	3.9	[2.0; 5.7]	1.9	[0.5; 3.3]	-6.8	[-7.1; -6.5]	-18.1	[-18.8; -17.4]
**Ghana**	11.9	[9.5; 14.3]	9.1	[6.0; 12.2]	13.5	[9.0; 17.9]	9.8	[5.1; 14.6]	7.6	[3.5; 11.7]	-3.4	[-3.7; -3.1]	-4.5	[-4.8; -4.1]
**Guinea**	20.3	[16.0; 24.6]	20.7	[17.0; 24.5]	20.4	[16.2; 24.6]	19.5	[14.9; 24.1]	12.7	[8.5; 16.9]	-7.1	[-7.9; -6.3]	-7.4	[-8.0; -6.8]
**Guinea Bissau**	13.1	[10.4; 15.9]	10.1	[7.2; 13.1]	9.4	[6.7; 12.1]	9.7	[6.3; 13.1]	15.9	[11.1; 20.7]	-2.2	[-3.1; -1.3]	3.2	[2.2; 4.1]
**Mali**	19.7	[16.3; 23.0]	13.7	[11.1; 16.4]	12.3	[9.7; 14.8]	10.9	[8.5; 13.3]	8.3	[6.5; 10.0]	-15.4	[-15.9; -14.9]	-14.8	[-15.3; -14.3]
**Mauritania**	20.9	[16.7; 25.1]	19.2	[15.2; 23.1]	19.4	[15.2; 23.6]	16.6	[13.0; 20.2]	15.8	[11.4; 20.2]	-7.1	[-7.8; -6.5]	-7.1	[-7.6; -6.6]
**Nigeria**	25.9	[23.2; 28.6]	26.4	[23.9; 28.9]	24.7	[20.8; 28.5]	16.2	[13.6; 18.8]	9.8	[8.1; 11.5]	-21.0	[-21.4; -20.6]	-16.3	[-16.6; -16.1]
**S Tome and Principe**	24.5	[18.5; 30.6]	16.0	[9.7; 22.3]	20.2	[11.3; 29.0]	21.1	[14.0; 28.2]	15.2	[7.7; 22.7]	-5.6	[-7.3; -3.9]	-7.1	[-8.3; -5.9]
**Sierra Leone**	29.7	[25.6; 33.9]	21.7	[18.4; 25.0]	20.2	[16.4; 24.1]	19.4	[15.4; 23.4]	17.0	[13.1; 21.0]	-15.1	[-15.5; -14.7]	-9.8	[-10.1; -9.5]
**Togo**	37.7	[32.8; 42.7]	23.9	[17.8; 30.1]	28.4	[23.3; 33.6]	24.5	[19.0; 29.9]	27.0	[21.3; 32.6]	-16.0	[-16.9; -15.2]	-4.3	[-4.7; -3.9]
							**Eastern & Southern Africa**							
**Burundi**	42.4	[38.8; 45.9]	40.1	[36.1; 44.1]	32.8	[29.4; 36.3]	36.2	[31.9; 40.6]	25.2	[20.7; 29.7]	-19.4	[-20.0; -18.9]	-10.1	[-10.4; -9.8]
**Eswatini**	4.6	[1.7; 7.5]	6.9	[3.3; 10.5]	7.9	[3.9; 11.9]	4.0	[0.8; 7.2]	0.4	[0.0; 1.4]	-2.9	[-3.8; -2.0]	-11.4	[-13.4; -9.5]
**Malawi**	23.4	[20.2; 26.6]	20.0	[17.3; 22.7]	19.1	[16.5; 21.6]	16.0	[13.5; 18.5]	14.0	[10.9; 17.2]	-11.6	[-12.1; -11.2]	-8.8	[-9.1; -8.4]
**Rwanda**	16.8	[13.5; 20.0]	16.2	[12.9; 19.5]	12.3	[9.3; 15.3]	10.2	[7.2; 13.2]	7.8	[5.0; 10.5]	-12.8	[-13.3; -12.3]	-11.5	[-12.1; -10.9]
**Uganda**	16.5	[13.9; 19.1]	16.4	[13.3; 19.5]	12.4	[9.8; 15.1]	11.6	[9.0; 14.2]	8.2	[5.8; 10.6]	-10.6	[-10.9; -10.4]	-11.1	[-11.4; -10.9]
**Zimbabwe**	13.0	[10.5; 15.5]	12.3	[9.6; 15.0]	9.4	[7.3; 11.5]	8.2	[6.1; 10.3]	6.3	[4.5; 8.1]	-10.2	[-10.9; -9.4]	-12.5	[-13.4; -11.7]
							**Middle East & North Africa**							
**Algeria**	9.9	[8.0; 11.9]	10.8	[7.5; 14.1]	8.7	[6.6; 10.8]	9.8	[7.6; 12.0]	9.6	[7.2; 12.0]	-1.0	[-1.3; -0.6]	-2.7	[-3.2; -2.2]
**Iraq**	13.9	[12.2; 15.7]	10.6	[8.9; 12.3]	9.7	[7.9; 11.4]	9.7	[7.7; 11.7]	5.5	[3.8; 7.1]	-9.3	[-9.5; -9.1]	-14.1	[-14.4; -13.8]
**Jordan**	10.8	[8.0; 13.6]	9.4	[6.2; 12.7]	8.4	[5.4; 11.5]	8.7	[5.0; 12.5]	8.2	[2.8; 13.6]	-3.3	[-3.3; -3.2]	-7.4	[-7.5; -7.2]
**State of Palestine**	9.1	[6.6; 11.6]	8.2	[6.0; 10.4]	6.6	[4.8; 8.4]	6.3	[4.5; 8.2]	4.4	[2.6; 6.2]	-6.7	[-7.2; -6.2]	-14.3	[-15.2; -13.3]
**Tunisia**	8.2	[4.2; 12.2]	4.3	[2.0; 6.7]	4.2	[1.6; 6.9]	7.7	[3.3; 12.1]	7.5	[2.2; 12.7]	-0.4	[-1.4; 0.6]	4.2	[2.6; 5.8]
							**Europe & Central Asia**							
**Belarus**	0.0	-	0.0	-	0.0	-	0.1	[0.0; 0.4]	0.0	[0.0; 0.0]	0.6	[0.5; 0.8]	22.2	[17.0; 27.4]

**Bosnia and Herzegovina**	1.0	[0.0; 2.5]	0.4	[0.0; 1.3]	1.4	[0.0; 3.0]	0.4	[0.0; 1.2]	1.2	[0.0; 2.4]	0.2	[0.1; 0.2]	-5.4	[-5.8; -5.0]
**Kazakhstan**	2.3	[0.7; 3.8]	2.0	[0.2; 3.8]	1.2	[0.3; 2.1]	2.3	[0.7; 3.9]	1.3	[0.3; 2.2]	-1.1	[-1.4; -0.8]	-7.4	[-9.3; -5.5]
**Kosovo**	3.4	[0.1; 6.7]	4.8	[0.9; 8.6]	3.3	[0.0; 6.9]	1.1	[0.0; 3.1]	2.4	[0.0; 5.1]	-3.6	[-4.5; -2.7]	-16.6	[-20.4; -12.8]
**Kyrgyzstan**	8.7	[5.1; 12.2]	5.4	[2.4; 8.4]	5.7	[2.8; 8.6]	4.4	[1.4; 7.3]	6.4	[2.9; 10.0]	-4.8	[-6.0; -3.6]	-2.4	[-4.3; -0.6]
**Macedonia**	2.7	[0.0; 5.6]	3.1	[0.0; 6.9]	0.7	[0.0; 2.2]	0.0	-	0.0	-	-6.0	[-6.6; -5.4]	-44.5	[-48.6; -40.5]
**Moldova**	1.1	[0.0; 2.8]	0.0	-	0.6	[0.0; 2.3]	0.0	-	0.0	-	-2.8	[-3.5; -2.0]	-66.6	[-72.5; -60.7]
**Montenegro**	1.5	[0.0; 4.3]	1.1	[0.0; 2.5]	0.0	-	1.5	[0.0; 4.9]	0.9	[0.0; 2.2]	-0.5	[-1.1; 0.2]	-12.0	[-18.6; -5.3]
**Turkmenistan**	3.3	[0.8; 5.8]	4.0	[1.2; 6.9]	2.5	[0.0; 5.1]	1.8	[0.3; 3.3]	0.8	[0.0; 1.8]	-7.0	[-8.0; -5.9]	-24.6	[-27.3; -22.0]
**Ukraine**	2.2	[0.7; 3.6]	1.1	[0.2; 2.1]	1.5	[0.3; 2.7]	1.2	[0.2; 2.2]	0.7	[0.1; 1.3]	-3.2	[-3.8; -2.6]	-16.3	[-19.0; -13.6]
**South Asia**														
**Bangladesh**	12.6	[10.8; 14.4]	12.4	[10.6; 14.2]	13.8	[11.5; 16.1]	12.7	[10.1; 15.3]	8.2	[5.7; 10.7]	-2.7	[-2.9; -2.5]	-5.3	[-5.6; -5.1]
**Bhutan**	6.2	[3.6; 8.7]	6.5	[4.0; 8.9]	6.1	[3.7; 8.5]	6.8	[3.8; 9.7]	4.6	[2.4; 6.7]	-1.7	[-2.1; -1.3]	-6.0	[-6.9; -5.2]
**Nepal**	14.3	[11.2; 17.4]	21.7	[15.4; 27.9]	25.1	[17.6; 32.5]	15.7	[11.0; 20.3]	10.1	[5.9; 14.4]	1.4	[0.5; 2.2]	-6.5	[-7.1; -6.0]
**East Asia & the Pacific**														
**Cambodia**	14.4	[10.8; 17.9]	9.8	[6.8; 12.8]	10.1	[6.8; 13.4]	10.5	[7.0; 14.0]	5.0	[2.9; 7.0]	-10.9	[-11.2; -10.6]	-19.5	[-19.8; -19.1]
**Lao**	8.8	[6.3; 11.3]	8.7	[6.3; 11.1]	4.6	[2.6; 6.5]	3.1	[1.7; 4.5]	1.4	[0.3; 2.4]	-10.6	[-10.9; -10.4]	-26.0	[-26.4; -25.6]
**Mongolia**	3.0	[1.5; 4.5]	2.1	[0.8; 3.4]	1.1	[0.2; 1.9]	0.9	[0.0; 1.7]	1.2	[0.1; 2.3]	-3.4	[-3.7; -3.1]	-21.0	[-22.4; -19.7]
**Thailand**	0.5	[0.0; 1.2]	0.3	[0.0; 0.8]	0.5	[0.0; 1.4]	0.4	[0.0; 0.9]	0.1	[0.0; 0.3]	-1.5	[-1.8; -1.2]	-13.7	[-16.2; -11.3]
**Vietnam**	12.4	[7.7; 17.1]	2.2	[0.4; 4.1]	2.2	[0.4; 4.0]	3.6	[1.5; 5.7]	2.6	[0.4; 4.9]	-14.5	[-16.1; -13.0]	-39.8	[-42.0; -37.6]
**Latin America & Caribbean**														
**Argentina**	3.7	[1.5; 6.0]	1.7	[0.0; 3.5]	1.1	[0.3; 2.0]	1.3	[0.0; 2.9]	1.6	[0.3; 2.8]	-3.0	[-3.1; -2.9]	-24.3	[-24.8; -23.8]
**Belize**	7.0	[3.0; 11.1]	4.9	[0.6; 9.2]	5.8	[2.8; 8.9]	2.5	[0.0; 5.1]	2.3	[0.1; 4.4]	-8.1	[-9.3; -6.9]	-13.5	[-16.2; -10.8]
**Costa Rica**	1.1	[0.0; 2.5]	0.2	[0.0; 0.6]	0.0	-	0.0	-	0.0	-	-4.1	[-4.3; -3.9]	-63.0	[-68.1; -57.9]
**Dominican Republic**	3.3	[1.9; 4.7]	1.3	[0.7; 1.9]	1.5	[0.8; 2.3]	1.9	[1.0; 2.7]	2.3	[0.9; 3.7]	-3.2	[-3.8; -2.6]	-12.2	[-13.8; -10.6]
**El Salvador**	4.0	[1.9; 6.2]	3.5	[1.7; 5.3]	1.5	[0.7; 2.3]	0.7	[0.1; 1.2]	1.6	[0.5; 2.8]	-6.7	[-7.3; -6.0]	-25.1	[-27.0; -23.2]
**Guyana**	5.9	[3.0; 8.8]	3.9	[0.9; 7.0]	1.8	[0.3; 3.3]	1.6	[0.0; 3.1]	1.9	[0.2; 3.7]	-9.8	[-10.8; -8.7]	-30.9	[-33.7; -28.1]
**Jamaica**	4.4	[0.9; 7.9]	2.6	[0.2; 5.0]	0.4	[0.0; 1.3]	4.0	[0.2; 7.9]	2.0	[0.0; 5.7]	-2.3	[-2.6; -1.9]	-13.0	[-13.9; -12.2]
**Mexico**	2.0	[1.0; 3.0]	1.1	[0.4; 1.8]	1.0	[0.1; 2.0]	1.0	[0.2; 1.7]	1.1	[0.1; 2.0]	-3.4	[-3.9; -2.9]	-16.1	[-18.4; -13.9]
**Panama**	6.0	[3.9; 8.2]	3.3	[0.0; 7.4]	3.1	[0.0; 6.9]	2.4	[0.0; 5.7]	1.4	[0.0; 3.8]	-8.4	[-8.8; -7.9]	-33.9	[-35.0; -32.8]
**Paraguay**	5.0	[1.5; 8.5]	3.1	[0.9; 5.2]	3.7	[1.4; 6.0]	1.3	[0.2; 2.3]	1.6	[0.0; 3.6]	-6.5	[-7.1; -5.9]	-28.3	[-30.3; -26.4]
**Suriname**	4.0	[2.4; 5.6]	1.0	[0.0; 2.6]	2.8	[0.0; 6.0]	2.3	[0.0; 5.5]	2.5	[0.0; 5.6]	-3.5	[-3.7; -3.3]	-19.3	[-19.9; -18.7]
**Trinidad and Tobago**	3.0	[0.0; 6.3]	3.9	[0.0; 8.3]	2.7	[0.0; 5.8]	3.2	[0.0; 7.9]	1.7	[0.0; 5.0]	-1.0	[-1.2; -0.8]	-3.8	[-4.4; -3.2]
**Uruguay**	3.6	[0.0; 8.9]	1.9	[0.0; 4.6]	0.0	-	0.0	-	0.6	[0.0; 1.7]	-6.2	[-6.4; -5.9]	-26.5	[-27.3; -25.7]

Note: [1] Slope Index of Inequality; [2] Concentration Index

Table S5. Prevalence of suspected developmental delay in each studied country for the literacy-numeracy domain; countries grouped by world region. Source: MICS and DHS surveys, 2010-2016

	Wealth quintile (%)										Measures of inequality			
**Country**	**Poorest**	**IC95%**	**Q2**	**IC95%**	**Q3**	**IC95%**	**Q4**	**IC95%**	**Richest**	**IC95%**	**SII[1]**	**IC95%**	**CIX[2]**	**IC95%**
**West & Central Africa**														
**Benin**	99.0	[98.3; 99.8]	98.1	[97.0; 99.3]	95.9	[94.4; 97.5]	91.1	[88.6; 93.6]	74.6	[70.5; 78.8]	-37.9	[-39.0; -36.8]	-6.4	[-6.7; -6.1]
**Cameroon**	98.6	[97.4; 99.8]	91.1	[88.6; 93.6]	84.6	[80.4; 88.9]	73.5	[68.3; 78.7]	58.8	[53.1; 64.4]	-44.0	[-45.4; -42.5]	-8.3	[-8.7; -7.9]
**CAR**	98.6	[97.8; 99.4]	97.2	[95.7; 98.6]	93.5	[90.9; 96.0]	93.3	[91.2; 95.5]	77.5	[71.9; 83.2]	-18.3	[-19.0; -17.5]	-2.3	[-2.5; -2.1]
**Chad**	96.8	[95.3; 98.3]	96.4	[94.9; 97.9]	95.3	[93.6; 97.1]	95.4	[93.5; 97.3]	87.2	[83.6; 90.8]	-9.2	[-9.4; -8.9]	-1.4	[-1.5; -1.3]
**Congo Brazzaville**	95.3	[93.8; 96.7]	90.9	[88.0; 93.9]	85.9	[82.2; 89.6]	76.9	[71.7; 82.2]	78.0	[72.4; 83.7]	-22.8	[-23.8; -21.9]	-3.9	[-4.1; -3.8]
**Congo DR**	89.2	[84.7; 93.6]	92.8	[90.0; 95.6]	91.4	[88.1; 94.7]	89.9	[86.1; 93.7]	81.1	[75.2; 87.0]	-3.1	[-3.3; -2.9]	-0.7	[-0.7; -0.6]
**Côte D'Ivoire**	99.1	[98.4; 99.7]	98.0	[96.7; 99.4]	97.5	[96.3; 98.8]	89.9	[86.2; 93.6]	67.2	[59.0; 75.4]	-27.9	[-29.1; -26.7]	-3.7	[-4.0; -3.5]
**Gambia**	92.6	[90.0; 95.2]	89.6	[85.6; 93.5]	89.8	[86.2; 93.4]	89.8	[86.0; 93.7]	73.5	[66.6; 80.4]	-13.6	[-14.5; -12.8]	-3.1	[-3.3; -2.9]
**Ghana**	93.0	[91.1; 94.9]	81.1	[75.9; 86.2]	73.4	[68.1; 78.7]	60.0	[52.5; 67.6]	37.5	[29.7; 45.4]	-50.5	[-51.9; -49.1]	-10.0	[-10.4; -9.6]
**Guinea**	99.1	[98.4; 99.8]	99.2	[98.6; 99.9]	97.6	[96.1; 99.1]	91.3	[88.5; 94.2]	78.7	[73.2; 84.2]	-27.2	[-28.5; -26.0]	-3.6	[-3.9; -3.4]
**Guinea Bissau**	98.9	[98.3; 99.5]	97.2	[95.2; 99.1]	97.5	[96.1; 98.9]	87.7	[83.0; 92.5]	73.0	[65.9; 80.1]	-24.8	[-26.2; -23.3]	-2.8	[-3.0; -2.5]
**Mali**	98.6	[97.8; 99.4]	94.9	[93.1; 96.6]	91.6	[88.8; 94.4]	90.8	[88.2; 93.4]	79.4	[76.4; 82.4]	-22.1	[-22.4; -21.8]	-3.5	[-3.6; -3.4]
**Mauritania**	78.8	[75.1; 82.4]	77.7	[73.5; 81.9]	71.2	[66.9; 75.4]	71.5	[66.6; 76.4]	59.6	[54.1; 65.2]	-20.7	[-21.5; -19.8]	-4.9	[-5.1; -4.7]
**Nigeria**	95.7	[94.7; 96.6]	90.1	[88.6; 91.5]	75.6	[72.5; 78.7]	50.9	[47.0; 54.9]	26.2	[22.9; 29.5]	-77.3	[-77.6; -77.0]	-19.3	[-19.5; -19.1]
**S Tome and Principe**	88.2	[83.7; 92.6]	84.6	[77.7; 91.6]	83.5	[77.6; 89.4]	84.2	[77.5; 91.0]	79.5	[70.3; 88.7]	-7.7	[-9.3; -6.0]	-2.1	[-2.4; -1.8]
**Sierra Leone**	96.3	[94.6; 98.0]	95.7	[93.9; 97.6]	91.4	[89.1; 93.8]	90.9	[88.1; 93.7]	70.2	[64.2; 76.1]	-26.1	[-27.1; -25.1]	-4.6	[-4.8; -4.3]
**Togo**	98.1	[97.3; 99.0]	98.0	[96.8; 99.2]	96.8	[95.1; 98.5]	86.7	[82.2; 91.2]	79.1	[74.4; 83.9]	-25.3	[-26.5; -24.1]	-3.1	[-3.4; -2.9]
**Eastern & Southern Africa**														
**Burundi**	95.9	[94.6; 97.1]	94.7	[93.1; 96.3]	94.1	[92.3; 96.0]	91.3	[89.0; 93.6]	77.1	[73.6; 80.7]	-22.1	[-22.7; -21.5]	-4.4	[-4.6; -4.1]
**Eswatini**	94.1	[91.4; 96.8]	86.5	[81.9; 91.0]	86.5	[80.8; 92.2]	79.3	[72.1; 86.5]	58.3	[48.9; 67.7]	-31.9	[-34.3; -29.4]	-6.7	[-7.2; -6.2]
**Malawi**	91.4	[89.6; 93.3]	90.3	[88.3; 92.3]	84.1	[81.7; 86.4]	79.0	[76.1; 81.9]	63.5	[59.6; 67.4]	-30.4	[-30.9; -29.9]	-5.4	[-5.6; -5.2]
**Rwanda**	94.4	[92.1; 96.7]	95.1	[93.3; 96.9]	95.4	[93.6; 97.3]	94.6	[92.2; 96.9]	81.6	[77.3; 85.9]	-11.7	[-12.3; -11.0]	-2.6	[-2.8; -2.4]
**Uganda**	87.1	[84.4; 89.8]	81.6	[78.8; 84.4]	72.3	[68.9; 75.8]	64.5	[60.3; 68.6]	43.3	[38.4; 48.1]	-46.3	[-47.0; -45.5]	-9.9	[-10.2; -9.6]
**Zimbabwe**	95.1	[93.5; 96.6]	95.1	[93.7; 96.6]	91.7	[89.7; 93.8]	89.0	[86.6; 91.5]	83.2	[79.7; 86.6]	-15.0	[-15.8; -14.1]	-2.7	[-2.8; -2.5]
**Middle East & North Africa**														
**Algeria**	81.3	[77.7; 84.9]	76.9	[73.1; 80.6]	70.0	[66.2; 73.8]	66.6	[62.7; 70.6]	58.7	[53.6; 63.8]	-26.3	[-27.1; -25.5]	-6.5	[-6.7; -6.3]
**Iraq**	91.6	[90.2; 93.0]	87.8	[85.6; 89.9]	82.5	[79.9; 85.1]	74.5	[70.9; 78.1]	64.1	[59.1; 69.2]	-27.3	[-27.7; -26.9]	-5.6	[-5.7; -5.6]
**Jordan**	89.9	[86.3; 93.4]	87.7	[84.5; 91.0]	85.4	[81.1; 89.6]	80.8	[74.9; 86.7]	67.4	[59.0; 75.8]	-18.1	[-19.2; -17.0]	-3.8	[-4.0; -3.6]
**State of Palestine**	85.1	[82.3; 87.9]	80.4	[77.1; 83.6]	83.0	[79.6; 86.3]	80.6	[77.2; 83.9]	74.6	[70.3; 78.8]	-11.2	[-13.2; -9.2]	-2.5	[-2.9; -2.1]
**Tunisia**	84.4	[78.2; 90.6]	69.3	[62.4; 76.2]	65.4	[57.0; 73.8]	62.7	[54.6; 70.8]	53.6	[44.3; 62.9]	-31.8	[-34.3; -29.4]	-7.3	[-8.0; -6.7]
**Europe & Central Asia**														
**Belarus**	67.9	[57.6; 78.1]	65.5	[57.6; 73.4]	50.0	[41.1; 59.0]	45.1	[37.1; 53.2]	41.4	[34.4; 48.3]	-31.4	[-34.4; -28.5]	-9.9	[-10.9; -8.9]

**Bosnia and Herzegovina**	83.2	[77.5; 88.9]	74.2	[65.5; 82.9]	79.0	[73.1; 84.9]	70.4	[58.4; 82.3]	68.9	[61.5; 76.3]	-15.0	[-16.9; -13.0]	-3.6	[-4.0; -3.1]
**Kazakhstan**	79.3	[73.1; 85.4]	77.8	[71.5; 84.2]	74.9	[67.6; 82.3]	66.7	[60.8; 72.5]	62.8	[57.0; 68.6]	-19.6	[-21.6; -17.7]	-5.0	[-5.5; -4.6]
**Kosovo**	87.1	[82.4; 91.9]	89.8	[84.5; 95.1]	90.0	[84.6; 95.4]	76.7	[68.5; 84.9]	67.2	[54.7; 79.8]	-24.2	[-26.8; -21.6]	-5.1	[-5.5; -4.6]
**Kyrgyzstan**	94.1	[91.7; 96.5]	92.5	[89.1; 95.9]	86.8	[82.6; 91.1]	82.2	[77.0; 87.3]	66.7	[58.8; 74.6]	-28.5	[-30.0; -26.9]	-4.1	[-4.5; -3.7]
**Macedonia**	66.0	[51.3; 80.8]	55.1	[44.4; 65.8]	54.7	[44.6; 64.9]	50.0	[39.1; 61.0]	57.9	[46.6; 69.3]	-9.6	[-13.4; -5.9]	-4.4	[-5.4; -3.4]
**Moldova**	85.0	[78.0; 91.9]	77.5	[69.6; 85.3]	73.3	[65.2; 81.4]	63.0	[53.3; 72.7]	54.4	[46.4; 62.5]	-33.1	[-37.5; -28.8]	-8.5	[-9.7; -7.4]
**Montenegro**	87.5	[81.0; 93.9]	84.5	[75.9; 93.1]	71.1	[59.2; 83.0]	71.9	[61.5; 82.4]	78.7	[70.6; 86.9]	-14.9	[-18.1; -11.6]	-3.9	[-4.6; -3.2]
**Turkmenistan**	89.5	[85.0; 94.0]	86.8	[81.8; 91.8]	75.7	[68.9; 82.5]	80.3	[74.7; 85.9]	79.1	[73.6; 84.6]	-13.3	[-15.3; -11.4]	-1.9	[-2.3; -1.5]
**Ukraine**	68.5	[60.5; 76.6]	60.9	[52.7; 69.1]	54.4	[43.7; 65.1]	45.1	[36.0; 54.2]	43.4	[34.4; 52.5]	-30.4	[-32.9; -27.9]	-9.6	[-10.4; -8.8]
**South Asia**														
**Bangladesh**	90.1	[88.7; 91.6]	86.3	[84.5; 88.2]	81.8	[79.3; 84.2]	76.1	[73.1; 79.0]	56.2	[51.9; 60.5]	-32.2	[-33.0; -31.3]	-6.0	[-6.2; -5.8]
**Bhutan**	87.4	[83.3; 91.4]	85.0	[80.9; 89.1]	81.8	[76.9; 86.6]	72.5	[66.9; 78.1]	47.7	[41.4; 54.1]	-40.3	[-41.6; -38.9]	-9.5	[-9.8; -9.1]
**Nepal**	89.6	[86.7; 92.5]	82.5	[77.8; 87.1]	79.1	[72.7; 85.4]	60.6	[52.8; 68.3]	31.9	[24.8; 39.1]	-52.5	[-54.2; -50.9]	-12.2	[-12.7; -11.6]
**East Asia & the Pacific**														
**Cambodia**	82.0	[77.5; 86.5]	78.3	[73.1; 83.5]	76.5	[71.4; 81.7]	68.7	[63.0; 74.4]	54.7	[49.4; 60.0]	-34.2	[-35.2; -33.2]	-8.1	[-8.4; -7.9]
**Lao**	93.5	[91.6; 95.5]	91.6	[89.7; 93.5]	82.3	[79.1; 85.6]	69.2	[65.0; 73.3]	43.4	[38.1; 48.6]	-48.0	[-48.9; -47.1]	-9.1	[-9.4; -8.8]
**Mongolia**	93.8	[91.7; 95.9]	94.3	[92.1; 96.4]	90.8	[88.2; 93.5]	89.9	[86.8; 93.0]	87.7	[84.0; 91.3]	-8.4	[-9.2; -7.6]	-1.5	[-1.6; -1.3]
**Thailand**	40.4	[33.6; 47.2]	30.4	[24.9; 35.9]	22.9	[18.1; 27.6]	25.3	[19.3; 31.3]	17.3	[11.8; 22.7]	-24.7	[-25.8; -23.7]	-15.4	[-15.9; -14.8]
**Vietnam**	83.0	[77.1; 89.0]	73.5	[65.7; 81.2]	67.5	[60.1; 74.9]	67.2	[60.2; 74.1]	66.5	[59.2; 73.9]	-21.6	[-23.8; -19.5]	-5.8	[-6.3; -5.3]
**Latin America & Caribbean**														
**Argentina**	68.2	[62.8; 73.6]	60.3	[54.3; 66.3]	52.2	[46.3; 58.1]	53.2	[46.7; 59.7]	51.1	[44.5; 57.7]	-20.3	[-21.8; -18.9]	-6.0	[-6.4; -5.6]
**Belize**	57.3	[47.5; 67.0]	53.8	[45.4; 62.3]	44.1	[35.8; 52.4]	38.9	[30.3; 47.6]	25.8	[16.7; 34.8]	-33.5	[-36.2; -30.7]	-10.3	[-11.4; -9.3]
**Costa Rica**	80.7	[73.1; 88.3]	83.9	[76.6; 91.2]	69.1	[50.9; 87.2]	62.3	[46.1; 78.6]	68.7	[53.9; 83.5]	-23.0	[-26.0; -20.0]	-4.8	[-5.4; -4.1]
**Dominican Republic**	90.2	[87.9; 92.6]	84.0	[81.5; 86.5]	81.1	[78.4; 83.7]	74.2	[70.1; 78.2]	61.0	[56.1; 65.9]	-30.6	[-31.3; -29.8]	-6.7	[-6.9; -6.5]
**El Salvador**	88.8	[85.8; 91.9]	87.2	[83.6; 90.9]	83.7	[80.0; 87.3]	77.3	[72.8; 81.9]	65.6	[58.8; 72.4]	-26.7	[-27.6; -25.9]	-5.7	[-5.8; -5.5]
**Guyana**	48.4	[42.8; 54.0]	33.6	[26.0; 41.3]	22.2	[16.6; 27.8]	25.7	[18.7; 32.7]	24.6	[16.9; 32.2]	-34.2	[-37.6; -30.9]	-13.4	[-14.9; -12.0]
**Jamaica**	50.8	[40.9; 60.8]	31.3	[22.9; 39.6]	25.2	[17.0; 33.5]	25.6	[16.6; 34.7]	30.6	[9.5; 51.6]	-27.1	[-30.5; -23.7]	-13.9	[-15.2; -12.6]
**Mexico**	86.3	[82.0; 90.6]	77.0	[71.8; 82.3]	74.5	[68.6; 80.4]	70.0	[61.2; 78.8]	81.6	[65.4; 97.7]	-16.0	[-17.3; -14.8]	-3.0	[-3.2; -2.8]
**Panama**	88.1	[83.5; 92.7]	87.3	[82.1; 92.5]	81.0	[74.2; 87.8]	74.3	[64.4; 84.3]	64.2	[53.8; 74.6]	-20.8	[-22.0; -19.7]	-4.9	[-5.1; -4.7]
**Paraguay**	93.4	[91.1; 95.8]	81.2	[74.9; 87.4]	77.3	[70.0; 84.6]	70.3	[63.5; 77.0]	56.0	[47.6; 64.4]	-41.6	[-43.0; -40.2]	-9.3	[-9.6; -8.9]
**Suriname**	91.7	[88.8; 94.5]	78.6	[71.5; 85.8]	72.8	[64.2; 81.5]	69.5	[60.0; 79.1]	62.6	[52.9; 72.3]	-34.7	[-36.0; -33.4]	-6.9	[-7.1; -6.6]
**Trinidad and Tobago**	27.1	[17.6; 36.5]	18.0	[8.6; 27.4]	14.4	[8.7; 20.2]	13.0	[5.9; 20.0]	7.5	[1.7; 13.4]	-21.3	[-25.0; -17.6]	-18.9	[-21.6; -16.2]
**Uruguay**	59.1	[40.2; 78.0]	45.0	[28.6; 61.4]	40.0	[21.8; 58.3]	59.3	[44.1; 74.4]	37.6	[28.5; 46.6]	-21.9	[-25.7; -18.0]	-8.3	[-9.5; -7.0]

Note: [1] Slope Index of Inequality; [2] Concentration Index

Table S6. Prevalence of suspected developmental delay in each studied country for the combined indicator (ECDI); countries grouped by world region. Source: MICS and DHS surveys, 2010-2016.

	Wealth quintile (%)										Measures of inequality			
**Country**	**Poorest**	**IC95%**	**Q2**	**IC95%**	**Q3**	**IC95%**	**Q4**	**IC95%**	**Richest**	**IC95%**	**SII[1]**	**IC95%**	**CIX[2]**	**IC95%**
**West & Central Africa**														
**Benin**	41.1	[37.1; 45.2]	40.2	[35.8; 44.6]	39.5	[35.2; 43.7]	39.1	[34.7; 43.5]	27.5	[24.0; 31.0]	-16.2	[-16.8; -15.6]	-7.9	[-8.2; -7.7]
**Cameroon**	41.7	[36.8; 46.5]	38.9	[34.3; 43.6]	43.3	[38.0; 48.5]	29.3	[24.8; 33.9]	24.6	[19.8; 29.4]	-20.1	[-21.1; -19.0]	-9.3	[-9.7; -8.8]
**CAR**	54.2	[49.1; 59.3]	54.5	[50.4; 58.7]	50.0	[44.9; 55.2]	51.0	[45.0; 57.0]	47.9	[41.9; 53.9]	-7.5	[-8.0; -7.1]	-1.8	[-1.9; -1.6]
**Chad**	64.6	[60.1; 69.2]	71.5	[67.3; 75.7]	71.0	[66.5; 75.5]	69.7	[65.1; 74.3]	57.2	[52.5; 62.0]	-6.9	[-7.5; -6.2]	-2.5	[-2.6; -2.4]
**Congo Brazzaville**	53.9	[50.4; 57.5]	43.2	[37.2; 49.2]	31.1	[25.7; 36.5]	34.4	[28.0; 40.8]	27.3	[21.5; 33.1]	-34.1	[-34.8; -33.3]	-11.6	[-11.9; -11.4]
**Congo DR**	39.2	[32.6; 45.9]	41.5	[35.5; 47.5]	35.3	[28.2; 42.3]	27.9	[20.2; 35.7]	22.5	[17.5; 27.4]	-12.8	[-13.1; -12.5]	-2.8	[-2.9; -2.7]
**Côte D'Ivoire**	36.4	[32.2; 40.6]	38.9	[34.0; 43.8]	38.9	[34.4; 43.4]	36.7	[30.1; 43.2]	27.3	[20.0; 34.6]	-4.2	[-4.9; -3.6]	-3.8	[-4.0; -3.6]
**Gambia**	33.0	[29.5; 36.4]	28.4	[23.8; 33.0]	34.8	[30.2; 39.4]	29.9	[24.9; 34.9]	24.4	[19.1; 29.7]	-6.3	[-6.9; -5.7]	-4.3	[-4.6; -4.1]
**Ghana**	29.7	[25.5; 33.9]	27.1	[22.1; 32.1]	28.5	[22.1; 35.0]	21.3	[15.7; 26.8]	14.3	[9.1; 19.6]	-14.2	[-15.2; -13.2]	-10.5	[-10.9; -10.0]
**Guinea**	54.1	[49.4; 58.9]	49.6	[44.8; 54.4]	53.2	[48.1; 58.4]	47.0	[42.0; 52.1]	47.7	[41.1; 54.4]	-7.6	[-8.2; -7.0]	-2.8	[-3.0; -2.6]
**Guinea Bissau**	31.8	[27.2; 36.5]	37.3	[32.5; 42.1]	40.6	[35.3; 45.9]	40.5	[34.3; 46.7]	40.0	[31.9; 48.0]	12.1	[11.3; 12.9]	1.3	[0.9; 1.6]
**Mali**	44.2	[40.1; 48.3]	36.9	[33.0; 40.9]	34.3	[30.7; 37.9]	36.8	[33.1; 40.5]	32.4	[29.0; 35.8]	-12.9	[-13.4; -12.5]	-5.1	[-5.3; -5.0]
**Mauritania**	46.0	[41.2; 50.8]	41.5	[37.5; 45.4]	37.1	[32.5; 41.6]	33.3	[28.6; 38.0]	31.1	[26.0; 36.1]	-19.4	[-20.1; -18.7]	-8.4	[-8.7; -8.2]
**Nigeria**	52.1	[49.1; 55.0]	49.3	[46.4; 52.3]	40.7	[36.2; 45.3]	26.4	[23.3; 29.4]	14.1	[12.1; 16.0]	-46.8	[-47.2; -46.3]	-21.1	[-21.2; -20.9]
**S Tome and Principe**	48.5	[40.9; 56.1]	43.3	[34.6; 52.0]	47.0	[37.5; 56.6]	46.0	[35.5; 56.6]	37.4	[26.5; 48.3]	-6.5	[-8.3; -4.7]	-4.4	[-5.0; -3.7]
**Sierra Leone**	60.7	[55.7; 65.6]	56.7	[52.3; 61.2]	53.5	[48.1; 58.9]	52.5	[47.7; 57.4]	40.9	[35.0; 46.8]	-20.2	[-20.8; -19.7]	-6.7	[-6.9; -6.5]
**Togo**	53.4	[47.9; 58.8]	43.6	[37.4; 49.8]	47.9	[42.0; 53.7]	41.4	[35.4; 47.4]	37.9	[31.4; 44.5]	-18.5	[-19.1; -17.8]	-5.2	[-5.5; -4.9]
**Eastern & Southern Africa**														
**Burundi**	64.4	[60.8; 67.9]	63.3	[59.8; 66.8]	59.6	[56.1; 63.2]	59.7	[55.3; 64.1]	46.2	[41.7; 50.7]	-19.9	[-20.6; -19.3]	-6.8	[-7.0; -6.5]
**Eswatini**	37.3	[31.3; 43.4]	36.7	[29.7; 43.6]	36.3	[29.0; 43.6]	31.4	[23.2; 39.5]	22.9	[14.9; 30.8]	-12.6	[-14.8; -10.5]	-7.6	[-8.6; -6.7]
**Malawi**	47.1	[43.6; 50.7]	41.8	[38.5; 45.0]	39.2	[35.8; 42.6]	35.9	[32.8; 38.9]	28.7	[24.8; 32.7]	-20.2	[-20.7; -19.6]	-7.5	[-7.8; -7.3]
**Rwanda**	34.7	[30.2; 39.2]	32.3	[28.1; 36.6]	27.0	[22.8; 31.2]	25.0	[20.7; 29.4]	18.4	[13.6; 23.1]	-20.0	[-20.6; -19.5]	-10.4	[-10.8; -10.0]
**Uganda**	43.4	[39.9; 46.9]	41.2	[37.5; 44.8]	34.7	[31.1; 38.2]	33.1	[29.1; 37.0]	18.3	[15.0; 21.7]	-26.8	[-27.4; -26.3]	-13.1	[-13.3; -12.8]
**Zimbabwe**	37.9	[34.1; 41.7]	38.7	[34.9; 42.5]	35.5	[31.5; 39.4]	37.6	[33.7; 41.5]	33.3	[29.2; 37.5]	-4.8	[-5.6; -4.1]	-1.7	[-2.0; -1.4]
**Middle East & North Africa**														
**Algeria**	28.5	[24.7; 32.2]	31.1	[27.4; 34.8]	29.4	[26.0; 32.9]	29.6	[25.7; 33.5]	27.4	[23.2; 31.6]	-1.5	[-1.9; -1.1]	-1.8	[-2.0; -1.6]
**Iraq**	33.1	[30.7; 35.5]	30.4	[27.5; 33.2]	27.6	[24.7; 30.4]	23.7	[20.7; 26.7]	18.9	[15.4; 22.4]	-15.4	[-15.7; -15.1]	-9.3	[-9.4; -9.1]
**Jordan**	38.0	[32.8; 43.1]	34.1	[28.9; 39.4]	25.5	[20.0; 30.9]	27.4	[21.6; 33.2]	24.3	[16.5; 32.1]	-17.9	[-18.3; -17.5]	-8.5	[-8.7; -8.2]
**State of Palestine**	35.1	[31.5; 38.7]	28.5	[25.1; 31.9]	25.8	[22.2; 29.4]	22.1	[18.8; 25.5]	16.8	[13.6; 20.0]	-22.0	[-23.0; -21.1]	-13.1	[-13.7; -12.5]
**Tunisia**	31.2	[24.6; 37.9]	20.2	[14.6; 25.7]	19.9	[13.0; 26.7]	23.1	[16.3; 29.8]	15.1	[9.0; 21.3]	-16.0	[-17.7; -14.2]	-10.5	[-11.5; -9.4]
**Europe & Central Asia**														
**Belarus**	6.8	[2.4; 11.3]	6.0	[2.2; 9.8]	6.2	[2.4; 10.0]	4.4	[2.1; 6.7]	4.5	[2.2; 6.9]	-3.1	[-3.6; -2.7]	-10.1	[-11.3; -8.8]

**Bosnia and Herzegovina**	4.5	[1.5; 7.4]	1.9	[0.0; 4.0]	3.4	[1.1; 5.7]	1.5	[0.0; 3.0]	5.2	[0.0; 11.1]	1.2	[0.7; 1.6]	-9.2	[-10.2; -8.2]
**Kazakhstan**	11.7	[8.0; 15.4]	15.9	[8.8; 23.0]	15.4	[10.5; 20.2]	11.2	[7.6; 14.7]	10.8	[7.0; 14.6]	-2.8	[-3.6; -2.0]	-5.3	[-6.2; -4.5]
**Kosovo**	16.6	[10.9; 22.3]	22.2	[14.8; 29.6]	21.0	[14.2; 27.7]	9.0	[3.9; 14.1]	10.4	[4.9; 16.0]	-11.7	[-13.3; -10.2]	-8.8	[-10.2; -7.4]
**Kyrgyzstan**	21.0	[15.9; 26.1]	18.3	[13.4; 23.3]	24.5	[19.1; 29.8]	19.1	[13.4; 24.8]	14.1	[9.4; 18.8]	-4.4	[-5.7; -3.1]	-4.9	[-5.9; -4.0]
**Macedonia**	8.5	[1.4; 15.7]	8.4	[3.4; 13.3]	8.9	[3.0; 14.8]	5.2	[1.6; 8.9]	3.3	[0.2; 6.4]	-7.3	[-8.2; -6.5]	-12.6	[-14.5; -10.7]
**Moldova**	24.6	[15.7; 33.5]	10.0	[4.4; 15.6]	15.7	[8.6; 22.9]	13.2	[6.8; 19.6]	12.1	[8.0; 16.3]	-8.4	[-10.2; -6.5]	-13.5	[-14.9; -12.2]
**Montenegro**	4.1	[0.4; 7.7]	6.8	[1.9; 11.6]	1.6	[0.0; 3.9]	7.4	[0.5; 14.2]	2.2	[0.0; 4.6]	-2.0	[-3.1; -0.8]	-3.2	[-6.5; 0.0]
**Turkmenistan**	11.5	[8.1; 14.9]	8.6	[5.0; 12.1]	5.0	[1.7; 8.4]	5.7	[2.6; 8.8]	6.8	[3.6; 9.9]	-6.3	[-7.3; -5.3]	-9.9	[-11.4; -8.5]
**Ukraine**	10.8	[7.3; 14.4]	9.0	[5.1; 13.0]	11.5	[6.9; 16.2]	7.6	[4.5; 10.7]	7.6	[4.0; 11.3]	-4.7	[-5.6; -3.8]	-6.7	[-8.0; -5.4]
**South Asia**														
**Bangladesh**	42.9	[40.4; 45.3]	38.7	[36.1; 41.3]	35.3	[32.2; 38.4]	33.7	[30.5; 36.8]	21.9	[18.6; 25.2]	-21.0	[-21.5; -20.4]	-9.6	[-9.8; -9.3]
**Bhutan**	31.5	[25.5; 37.4]	28.6	[24.2; 33.0]	27.4	[22.5; 32.4]	29.0	[22.4; 35.6]	19.5	[14.6; 24.4]	-11.5	[-12.3; -10.7]	-8.2	[-8.6; -7.7]
**Nepal**	39.8	[35.2; 44.4]	43.5	[37.0; 50.0]	42.0	[33.8; 50.2]	29.8	[23.9; 35.7]	13.1	[7.6; 18.5]	-23.0	[-24.4; -21.6]	-14.2	[-14.6; -13.8]
**East Asia & the Pacific**														
**Cambodia**	32.7	[28.4; 37.0]	27.3	[22.8; 31.8]	25.0	[20.2; 29.8]	27.8	[22.5; 33.2]	18.3	[14.0; 22.7]	-16.6	[-17.1; -16.1]	-12.1	[-12.4; -11.8]
**Lao**	23.1	[19.8; 26.4]	22.0	[19.0; 24.9]	17.7	[14.7; 20.7]	14.1	[11.1; 17.1]	8.8	[6.1; 11.4]	-16.4	[-16.7; -16.1]	-14.9	[-15.1; -14.7]
**Mongolia**	23.3	[20.0; 26.6]	23.9	[19.6; 28.2]	20.5	[16.5; 24.4]	26.2	[21.3; 31.0]	22.1	[17.7; 26.6]	-0.1	[-0.8; 0.6]	0.9	[0.4; 1.3]
**Thailand**	10.0	[6.6; 13.4]	7.1	[4.4; 9.9]	4.9	[2.8; 7.0]	7.3	[4.1; 10.4]	3.0	[1.3; 4.6]	-8.0	[-8.6; -7.4]	-17.3	[-18.3; -16.3]
**Vietnam**	16.9	[11.7; 22.2]	8.4	[4.1; 12.7]	8.1	[4.4; 11.7]	8.7	[5.1; 12.3]	6.9	[2.8; 11.1]	-13.2	[-14.6; -11.7]	-21.6	[-23.2; -19.9]
**Latin America & Caribbean**														
**Argentina**	20.1	[16.1; 24.0]	14.9	[10.4; 19.4]	9.3	[6.2; 12.4]	9.7	[5.3; 14.1]	7.2	[4.1; 10.3]	-17.5	[-18.1; -16.8]	-18.8	[-19.5; -18.2]
**Belize**	22.3	[16.5; 28.1]	17.1	[11.2; 23.0]	18.5	[13.0; 24.0]	7.8	[3.3; 12.2]	7.5	[3.2; 11.8]	-5.9	[-8.9; -2.9]	-8.3	[-12.6; -4.1]
**Costa Rica**	29.6	[19.6; 39.7]	14.2	[7.4; 20.9]	8.8	[4.4; 13.2]	20.4	[8.3; 32.4]	6.5	[0.6; 12.4]	-20.6	[-22.2; -18.9]	-16.3	[-17.9; -14.7]
**Dominican Republic**	21.6	[18.8; 24.3]	13.3	[11.2; 15.3]	12.7	[10.2; 15.2]	11.6	[8.8; 14.5]	11.8	[8.0; 15.6]	-27.9	[-29.6; -26.1]	-20.1	[-21.1; -19.2]
**El Salvador**	20.3	[17.0; 23.7]	21.0	[17.4; 24.5]	19.7	[15.7; 23.7]	14.3	[10.4; 18.2]	13.7	[9.4; 17.9]	-13.8	[-14.3; -13.2]	-13.1	[-13.6; -12.7]
**Guyana**	18.0	[14.2; 21.8]	8.3	[4.2; 12.4]	9.5	[5.3; 13.7]	8.5	[4.0; 13.1]	7.8	[4.0; 11.6]	-9.8	[-10.6; -9.1]	-9.0	[-9.7; -8.4]
**Jamaica**	19.5	[12.4; 26.7]	8.9	[4.2; 13.5]	5.1	[1.1; 9.2]	10.5	[4.1; 16.9]	2.8	[0.0; 6.6]	-17.6	[-19.7; -15.6]	-21.8	[-23.8; -19.7]
**Mexico**	20.1	[16.3; 23.8]	18.0	[14.0; 21.9]	18.7	[11.2; 26.2]	17.1	[12.2; 21.9]	9.6	[4.2; 14.9]	-18.3	[-20.3; -16.4]	-25.9	[-27.8; -24.0]
**Panama**	21.9	[17.7; 26.1]	24.4	[15.5; 33.4]	24.0	[15.9; 32.0]	11.8	[4.0; 19.6]	4.9	[1.0; 8.8]	-8.5	[-9.4; -7.7]	-6.8	[-7.5; -6.1]
**Paraguay**	22.3	[17.7; 26.9]	18.0	[13.0; 22.9]	21.1	[15.1; 27.1]	12.9	[8.9; 16.9]	9.1	[4.9; 13.3]	-12.7	[-13.9; -11.5]	-12.6	[-13.2; -12.0]
**Suriname**	40.2	[35.0; 45.4]	24.5	[17.6; 31.4]	18.3	[11.7; 24.9]	19.5	[11.1; 27.9]	25.1	[16.1; 34.1]	-15.8	[-16.7; -14.8]	-15.6	[-16.4; -14.8]
**Trinidad and Tobago**	7.4	[2.5; 12.2]	8.9	[2.2; 15.6]	5.2	[1.7; 8.7]	3.0	[0.0; 7.0]	4.3	[0.0; 9.1]	-33.7	[-34.8; -32.7]	-17.3	[-17.7; -17.0]
**Uruguay**	20.5	[5.9; 35.2]	8.9	[0.6; 17.1]	4.5	[1.1; 7.9]	17.0	[0.1; 34.0]	3.4	[0.5; 6.4]	-6.3	[-8.2; -4.3]	-5.4	[-8.9; -1.8]

Note: [1] Slope Index of Inequality; [2] Concentration Index

Table S7. Prevalence of suspected developmental delay in each studied country for the physical domain by urban/rural residence, sex of the child and maternal education; countries grouped by world region. Source: MICS and DHS surveys, 2010-2016.

		Area (%)				Sex (%)				Maternal education (%)					
**Country**	**Year**	**Urban**	**95% IC**	**Rural**	**95% IC**	**Female**	**95% IC**	**Male**	**95% IC**	**None**	**95% IC**	**Primary**	**95% IC**	**Secondary +**	**95% IC**
**West & Central Africa**															
**Benin**	2014	4.4	[2.9; 6.0]	5.2	[3.7; 6.7]	4.9	[3.5; 6.3]	4.8	[3.5; 6.2]	5.0	[3.7; 6.2]	5.5	[3.3; 7.6]	3.7	[0.8; 6.6]
**Cambodia**	2014	2.2	[1.3; 3.2]	2.6	[1.5; 3.7]	2.6	[1.6; 3.5]	2.3	[1.3; 3.4]	2.1	[1.1; 3.1]	2.6	[1.4; 3.8]	2.6	[1.4; 3.8]
**CAR**	2010	4.2	[2.7; 5.8]	3.7	[2.8; 4.7]	3.9	[2.7; 5.0]	4.0	[2.7; 5.3]	4.1	[2.9; 5.2]	3.9	[2.8; 5.1]	3.5	[0.8; 6.2]
**Chad**	2014	17.1	[13.8; 20.4]	16.3	[14.4; 18.2]	17.1	[14.8; 19.3]	15.9	[13.5; 18.2]	16.7	[14.9; 18.6]	15.8	[12.7; 19.0]	15.9	[10.9; 20.9]
**Congo Brazzaville**	2014	1.6	[0.9; 2.3]	4.7	[3.5; 6.0]	3.0	[2.0; 3.9]	2.6	[1.8; 3.4]	2.8	[1.3; 4.3]	3.5	[2.2; 4.8]	2.7	[1.7; 3.6]
**Congo DR**	2013	6.6	[4.4; 8.9]	7.2	[5.4; 9.0]	6.5	[4.7; 8.3]	7.6	[5.6; 9.6]	6.2	[3.9; 8.6]	7.4	[5.2; 9.7]	6.9	[4.7; 9.2]
**Côte D'Ivoire**	2016	2.3	[1.2; 3.4]	3.7	[2.5; 4.9]	2.9	[1.8; 4.0]	3.4	[2.1; 4.6]	3.2	[2.1; 4.3]	3.1	[1.3; 4.9]	3.0	[1.2; 4.8]
**Gambia**	2010	1.7	[0.7; 2.6]	2.4	[1.6; 3.2]	2.1	[1.3; 2.8]	2.1	[1.3; 2.9]	2.3	[1.5; 3.0]	1.7	[0.1; 3.2]	1.2	[0.1; 2.3]
**Ghana**	2011	1.9	[0.7; 3.0]	3.1	[2.1; 4.2]	2.3	[1.2; 3.4]	2.9	[1.8; 4.0]	2.9	[1.8; 4.0]	2.2	[0.8; 3.6]	2.5	[1.2; 3.9]
**Guinea**	2016	7.7	[4.8; 10.5]	7.5	[5.8; 9.1]	7.7	[5.9; 9.5]	7.4	[5.7; 9.1]	7.8	[6.2; 9.3]	5.5	[2.6; 8.5]	8.1	[3.9; 12.3]
**Guinea Bissau**	2014	12.0	[7.0; 17.1]	9.5	[7.6; 11.4]	10.9	[7.9; 14.0]	10.0	[7.7; 12.3]	11.0	[8.6; 13.4]	9.4	[6.2; 12.6]	9.9	[5.0; 14.9]
**Mali**	2015	4.4	[3.2; 5.6]	4.6	[3.7; 5.5]	4.8	[3.9; 5.8]	4.4	[3.5; 5.2]	4.6	[3.8; 5.5]	4.2	[2.6; 5.7]	4.7	[2.5; 6.9]
**Mauritania**	2015	6.9	[5.3; 8.5]	7.9	[6.2; 9.6]	7.3	[5.7; 9.0]	7.6	[6.0; 9.1]	7.5	[5.6; 9.4]	7.6	[5.2; 10.0]	8.4	[6.5; 10.4]
**Nigeria**	2016	7.2	[5.9; 8.4]	10.6	[9.5; 11.6]	10.1	[9.0; 11.3]	8.9	[8.0; 9.9]	12.6	[10.8; 14.4]	8.6	[7.1; 10.0]	7.0	[5.9; 8.1]
**S Tome and Principe**	2014	4.7	[2.3; 7.1]	4.8	[2.3; 7.3]	4.4	[2.0; 6.9]	5.0	[2.7; 7.3]	11.6	[0.0; 23.9]	4.4	[2.3; 6.5]	4.3	[1.7; 6.9]
**Sierra Leone**	2010	11.1	[8.6; 13.7]	10.5	[8.7; 12.3]	9.5	[7.9; 11.2]	11.7	[9.7; 13.7]	11.4	[9.7; 13.1]	9.5	[6.1; 12.9]	6.8	[3.9; 9.7]
**Togo**	2013	5.9	[3.5; 8.2]	7.1	[5.1; 9.0]	7.3	[5.0; 9.6]	6.0	[4.3; 7.7]	6.2	[4.2; 8.2]	7.4	[4.9; 9.8]	6.3	[3.6; 8.9]
**Eastern & Southern Africa**															
**Burundi**	2016	3.5	[1.6; 5.4]	7.8	[6.8; 8.8]	6.8	[5.6; 8.0]	8.1	[6.8; 9.4]	8.0	[6.7; 9.3]	7.4	[6.0; 8.8]	4.5	[2.4; 6.7]
**Eswatini**	2014	4.8	[1.3; 8.3]	7.1	[5.1; 9.2]	7.3	[4.9; 9.7]	6.0	[3.7; 8.3]	9.2	[3.1; 15.3]	8.3	[5.2; 11.3]	5.2	[3.1; 7.2]
**Malawi**	2013	7.4	[3.6; 11.2]	9.8	[8.7; 10.9]	9.6	[8.2; 11.0]	9.4	[8.1; 10.8]	11.1	[8.5; 13.7]	10.0	[8.6; 11.4]	5.7	[4.0; 7.4]
**Rwanda**	2014	5.4	[2.8; 8.0]	4.8	[3.7; 5.9]	4.7	[3.4; 6.0]	5.1	[3.8; 6.4]	6.8	[4.0; 9.6]	4.6	[3.5; 5.6]	4.1	[2.0; 6.3]
**Uganda**	2016	7.5	[5.1; 9.8]	9.9	[8.4; 11.4]	10.0	[8.4; 11.7]	8.7	[7.0; 10.4]	9.4	[6.5; 12.3]	10.5	[8.8; 12.2]	6.6	[4.7; 8.6]
**Zimbabwe**	2014	4.3	[3.0; 5.6]	5.7	[4.6; 6.9]	6.1	[4.8; 7.4]	4.6	[3.6; 5.6]	7.0	[3.5; 10.5]	5.9	[4.4; 7.4]	4.9	[3.9; 5.9]
**Middle East & North Africa**															
**Algeria**	2012	3.6	[2.7; 4.5]	4.8	[3.4; 6.2]	3.8	[2.8; 4.7]	4.4	[3.3; 5.5]	4.7	[2.9; 6.5]	5.7	[3.8; 7.6]	3.4	[2.5; 4.2]
**Iraq**	2011	5.3	[4.5; 6.1]	4.7	[3.7; 5.6]	4.9	[4.1; 5.8]	5.2	[4.4; 6.1]	5.5	[4.3; 6.8]	5.2	[4.4; 5.9]	4.7	[3.5; 5.9]
**Jordan**	2012	1.1	[0.5; 1.8]	0.8	[0.2; 1.4]	1.0	[0.3; 1.8]	1.1	[0.3; 1.9]	1.0	[0.0; 2.4]	0.4	[0.0; 0.9]	1.1	[0.5; 1.7]
**State of Palestine**	2014	1.8	[0.8; 2.7]	2.4	[0.9; 4.0]	1.9	[0.9; 2.9]	1.8	[0.9; 2.8]	8.5	[0.0; 22.9]	1.4	[0.6; 2.1]	2.1	[1.1; 3.1]
**Tunisia**	2011	2.8	[1.4; 4.3]	3.5	[1.7; 5.4]	3.1	[1.5; 4.7]	3.1	[1.4; 4.7]	3.9	[1.1; 6.7]	2.6	[0.9; 4.4]	3.2	[1.5; 4.9]
**Europe & Central Asia**															
**Belarus**	2012	0.6	[0.1; 1.1]	0.7	[0.0; 1.9]	0.9	[0.1; 1.6]	0.4	[0.0; 0.9]	NA	-	1.3	[0.0; 3.5]	0.6	[0.1; 1.1]
**Bosnia and Herzegovina**	2011	0.2	[0.0; 0.6]	0.3	[0.0; 0.7]	0.3	[0.0; 0.8]	0.3	[0.0; 0.6]	0.0	-	0.6	[0.0; 1.4]	0.2	[0.0; 0.5]
**Kazakhstan**	2015	0.6	[0.0; 1.3]	0.4	[0.0; 1.0]	0.4	[0.0; 0.8]	0.6	[0.0; 1.4]	0.0	-	25.0	-	0.5	[0.0; 1.0]
**Kosovo**	2013	1.0	[0.0; 2.7]	1.1	[0.0; 2.7]	0.9	[0.0; 2.2]	1.3	[0.0; 3.2]	0.0	-	0.0	-	1.2	[0.0; 2.9]
**Kyrgyzstan**	2014	2.4	[0.8; 4.1]	2.2	[1.1; 3.4]	3.0	[1.4; 4.6]	1.6	[0.7; 2.5]	0.0	-	12.8	[0.0; 36.3]	2.5	[1.3; 3.7]
**Macedonia**	2011	0.0	-	0.0	-	0.0	-	0.0	-	0.0	-	0.0	[0.0; 0.0]	0.0	[0.0; 0.1]
**Moldova**	2012	0.1	[0.0; 0.3]	0.6	[0.0; 1.6]	0.6	[0.0; 1.5]	0.2	[0.0; 0.8]	NA	-	0.0	-	0.4	[0.0; 1.1]

**Montenegro**	2013	0.0	-	0.0	-	0.0	-	0.0	-	0.0	-	0.0	[0.0; 0.0]	0.0	[0.0; 0.0]
**Turkmenistan**	2015	0.3	[0.0; 1.2]	0.2	[0.0; 0.7]	0.3	[0.0; 0.9]	0.2	[0.0; 0.9]	NA	-	0.0	-	0.3	[0.0; 0.9]
**Ukraine**	2012	0.4	[0.0; 0.9]	0.7	[0.0; 1.7]	0.4	[0.0; 1.0]	0.6	[0.0; 1.3]	NA	-	NA	-	0.5	[0.0; 1.1]
**South Asia**															
**Bangladesh**	2012	6.3	[4.4; 8.2]	7.5	[6.5; 8.6]	7.9	[6.6; 9.1]	6.8	[5.7; 7.9]	7.1	[5.6; 8.6]	7.8	[6.2; 9.4]	7.0	[5.7; 8.3]
**Bhutan**	2010	1.2	[0.0; 2.3]	2.1	[1.2; 2.9]	1.9	[0.9; 2.9]	1.7	[0.8; 2.6]	1.4	[0.6; 2.1]	3.5	[0.9; 6.0]	2.3	[0.5; 4.0]
**Nepal**	2014	0.8	[0.0; 1.6]	3.9	[2.7; 5.2]	3.3	[1.5; 5.0]	3.8	[2.5; 5.1]	3.6	[2.3; 4.8]	2.3	[0.7; 3.9]	4.1	[1.7; 6.5]
**East Asia & the Pacific**															
**Cameroon**	2014	3.5	[1.6; 5.4]	3.5	[2.4; 4.5]	3.0	[1.8; 4.2]	3.9	[2.6; 5.2]	3.2	[0.8; 5.6]	3.5	[2.3; 4.7]	3.6	[2.1; 5.2]
**Lao**	2011	0.5	[0.1; 0.9]	2.1	[1.5; 2.7]	1.8	[1.2; 2.4]	1.8	[1.2; 2.5]	2.5	[1.5; 3.5]	1.6	[1.0; 2.1]	1.1	[0.5; 1.8]
**Mongolia**	2013	0.6	[0.0; 1.1]	0.5	[0.0; 1.2]	0.5	[0.0; 1.1]	0.6	[0.0; 1.2]	0.0	-	1.0	[0.0; 2.4]	0.6	[0.0; 1.1]
**Thailand**	2015	1.4	[0.0; 3.2]	1.5	[0.4; 2.7]	1.5	[0.0; 3.2]	1.4	[0.3; 2.5]	0.4	[0.0; 0.9]	0.9	[0.2; 1.6]	0.8	[0.2; 1.3]
**Vietnam**	2013	1.6	[0.4; 2.8]	2.8	[1.2; 4.4]	2.2	[0.9; 3.6]	2.7	[1.0; 4.3]	3.6	[0.0; 8.0]	4.5	[0.9; 8.1]	1.9	[0.9; 2.8]
**Latin America & Caribbean**															
**Argentina**	2011	1.5	[0.8; 2.3]	NA	-	1.4	[0.5; 2.3]	1.7	[0.7; 2.7]	13.4	[0.0; 37.5]	1.2	[0.0; 2.4]	1.5	[0.7; 2.4]
**Belize**	2015	2.0	[0.0; 4.3]	1.7	[0.3; 3.2]	1.6	[0.3; 3.0]	2.1	[0.0; 4.3]	0.0	-	1.8	[0.3; 3.3]	3.2	[0.0; 6.7]
**Costa Rica**	2011	1.5	[0.3; 2.8]	0.5	[0.0; 1.3]	1.2	[0.0; 2.5]	1.0	[0.0; 2.1]	6.1	[0.0; 17.2]	1.2	[0.0; 2.8]	0.9	[0.0; 1.9]
**Dominican Republic**	2014	2.0	[1.3; 2.8]	2.0	[1.1; 2.9]	1.8	[1.0; 2.7]	2.2	[1.4; 3.0]	2.0	[0.3; 3.7]	1.7	[0.9; 2.5]	2.2	[1.4; 3.0]
**El Salvador**	2014	1.8	[1.1; 2.5]	2.4	[1.3; 3.4]	2.4	[1.5; 3.4]	1.7	[1.0; 2.5]	3.2	[1.1; 5.3]	2.5	[1.4; 3.7]	1.6	[1.0; 2.3]
**Guyana**	2014	1.6	[0.2; 2.9]	1.7	[0.6; 2.7]	1.5	[0.5; 2.5]	1.8	[0.6; 3.0]	3.1	[0.0; 7.7]	3.1	[0.5; 5.8]	1.3	[0.5; 2.2]
**Jamaica**	2011	1.0	[0.2; 1.8]	1.7	[0.0; 3.9]	0.7	[0.0; 1.4]	1.8	[0.0; 3.7]	NA	-	0.0	-	1.4	[0.2; 2.6]
**Mexico**	2015	0.6	[0.0; 1.2]	0.8	[0.0; 1.7]	0.5	[0.0; 1.2]	0.7	[0.0; 1.5]	0.2	[0.0; 0.7]	0.6	[0.0; 1.2]	0.6	[0.0; 1.4]
**Panama**	2013	0.4	[0.0; 1.1]	1.1	[0.5; 1.6]	1.1	[0.1; 2.1]	0.3	[0.0; 0.6]	0.4	[0.0; 1.1]	1.3	[0.4; 2.1]	0.5	[0.0; 1.1]
**Paraguay**	2016	1.4	[0.3; 2.4]	0.9	[0.0; 1.9]	1.3	[0.1; 2.4]	1.1	[0.2; 2.1]	2.4	[0.0; 7.9]	1.2	[0.0; 2.4]	1.6	[0.1; 3.1]
**Suriname**	2010	1.3	[0.2; 2.4]	3.4	[2.1; 4.7]	2.0	[0.9; 3.2]	2.2	[1.0; 3.4]	4.7	[1.9; 7.6]	1.8	[0.8; 2.8]	1.6	[0.3; 2.8]
**Trinidad and Tobago**	2011	2.4	[0.4; 4.3]	0.6	[0.0; 1.8]	2.2	[0.2; 4.2]	0.9	[0.0; 2.2]	0.0	-	0.0	-	1.9	[0.4; 3.4]
**Uruguay**	2012	2.2	[0.0; 4.5]	NA	-	3.3	[0.0; 8.1]	1.1	[0.0; 2.6]	NA	-	2.2	[0.0; 4.4]	2.1	[0.0; 4.9]

Note: Data not available marked as NA

Table S8. Prevalence of suspected developmental delay in each studied country for the social-emotional domain by urban/rural residence, sex of the child and maternal education; countries grouped by world region. Source: MICS and DHS surveys, 2010-2016.

		**Area (%)**				**Sex (%)**				**Maternal education (%)**					
**Country**	**Year**	**Urban**	**95% IC**	**Rural**	**95% IC**	**Female**	**95% IC**	**Male**	**95% IC**	**None**	**95% IC**	**Primary**	**95% IC**	**Secondary +**	**95% IC**
**West & Central Africa**															
**Benin**	2014	28.6	[25.8; 31.5]	28.3	[25.6; 31.1]	24.3	[21.8; 26.8]	32.8	[30.1; 35.5]	28.1	[25.8; 30.4]	30.0	[26.1; 33.8]	27.5	[22.3; 32.7]
**Cameroon**	2014	30.6	[27.3; 33.8]	32.4	[29.5; 35.3]	29.2	[26.3; 32.0]	34.1	[31.0; 37.1]	27.2	[23.4; 30.9]	34.4	[30.9; 37.8]	33.4	[29.7; 37.0]
**CAR**	2010	45.6	[40.2; 51.1]	40.5	[37.3; 43.8]	39.7	[36.5; 42.8]	45.2	[41.6; 48.8]	39.3	[35.5; 43.2]	42.3	[38.8; 45.8]	50.3	[43.3; 57.4]
**Chad**	2014	39.0	[34.3; 43.8]	40.7	[37.8; 43.7]	38.4	[35.4; 41.5]	42.4	[39.3; 45.5]	39.9	[37.1; 42.7]	42.3	[37.0; 47.6]	39.9	[33.2; 46.6]
**Congo Brazzaville**	2014	30.8	[26.9; 34.7]	45.2	[42.6; 47.8]	31.5	[28.0; 34.9]	40.4	[36.7; 44.1]	38.1	[31.7; 44.5]	37.9	[33.6; 42.3]	36.1	[32.1; 40.1]
**Congo DR**	2013	20.0	[15.9; 24.1]	21.8	[17.6; 26.0]	18.3	[15.0; 21.7]	24.3	[20.3; 28.3]	17.9	[13.5; 22.3]	24.6	[19.8; 29.4]	18.8	[15.2; 22.4]
**Côte D'Ivoire**	2016	34.9	[29.7; 40.0]	28.7	[26.3; 31.0]	28.2	[24.9; 31.6]	33.7	[30.6; 36.7]	30.0	[27.3; 32.7]	31.4	[26.3; 36.4]	35.7	[28.9; 42.5]
**Gambia**	2010	32.6	[28.6; 36.6]	30.3	[26.9; 33.6]	30.2	[27.1; 33.4]	32.3	[29.1; 35.4]	30.8	[27.7; 33.8]	36.4	[30.3; 42.5]	30.8	[25.8; 35.7]
**Ghana**	2011	28.1	[23.7; 32.4]	25.4	[22.4; 28.4]	22.3	[18.8; 25.7]	30.8	[27.2; 34.3]	25.0	[21.5; 28.5]	29.4	[23.8; 34.9]	26.6	[22.4; 30.7]
**Guinea**	2016	38.2	[34.3; 42.1]	38.6	[35.6; 41.6]	35.5	[32.5; 38.6]	41.3	[38.2; 44.3]	38.4	[35.8; 41.1]	39.0	[33.3; 44.7]	38.4	[32.2; 44.6]
**Guinea Bissau**	2014	30.5	[25.4; 35.5]	24.2	[21.5; 27.0]	22.7	[19.9; 25.5]	30.5	[26.7; 34.2]	25.4	[22.5; 28.3]	29.4	[24.9; 34.0]	26.6	[19.9; 33.2]
**Mali**	2015	30.3	[26.6; 34.1]	26.3	[24.4; 28.3]	24.3	[22.2; 26.5]	29.7	[27.6; 31.8]	26.4	[24.6; 28.2]	29.3	[25.3; 33.2]	31.9	[25.5; 38.3]
**Mauritania**	2015	31.5	[27.9; 35.1]	35.7	[32.3; 39.1]	32.9	[30.0; 35.9]	34.9	[31.7; 38.0]	30.6	[27.2; 33.9]	38.7	[34.4; 43.1]	34.2	[30.6; 37.8]
**Nigeria**	2016	26.8	[24.2; 29.5]	29.4	[27.8; 31.0]	27.4	[25.7; 29.1]	29.8	[28.1; 31.5]	29.0	[26.6; 31.4]	28.1	[25.0; 31.2]	27.1	[24.8; 29.4]
**S Tome and Principe**	2014	40.3	[35.3; 45.2]	32.0	[26.3; 37.7]	38.4	[34.1; 42.7]	36.6	[31.1; 42.0]	46.4	[30.0; 62.9]	40.4	[35.2; 45.5]	29.9	[23.6; 36.2]
**Sierra Leone**	2010	39.7	[35.6; 43.9]	41.1	[37.5; 44.8]	39.3	[35.8; 42.8]	42.2	[38.6; 45.7]	40.9	[37.8; 43.9]	41.4	[35.2; 47.7]	39.4	[33.2; 45.5]
**Togo**	2013	24.0	[20.3; 27.7]	24.5	[21.6; 27.4]	23.6	[20.6; 26.5]	25.1	[22.1; 28.2]	25.8	[22.5; 29.0]	23.2	[19.6; 26.8]	23.4	[19.3; 27.5]
**Eastern & Southern Africa**															
**Burundi**	2016	45.2	[39.2; 51.2]	40.2	[37.8; 42.6]	33.8	[31.0; 36.6]	47.4	[44.5; 50.2]	39.4	[36.5; 42.3]	41.8	[38.9; 44.8]	42.1	[36.1; 48.2]
**Eswatini**	2014	31.8	[23.7; 39.9]	35.5	[31.4; 39.6]	34.7	[30.0; 39.5]	34.8	[29.9; 39.6]	38.4	[28.8; 47.9]	35.3	[29.8; 40.9]	33.7	[29.1; 38.4]
**Malawi**	2013	30.8	[26.1; 35.5]	27.9	[26.2; 29.5]	23.9	[21.9; 25.9]	32.5	[30.2; 34.7]	28.1	[24.3; 31.9]	28.0	[26.3; 29.7]	29.1	[24.8; 33.4]
**Rwanda**	2014	17.0	[9.8; 24.2]	18.1	[15.6; 20.5]	16.1	[13.5; 18.8]	19.6	[16.8; 22.4]	20.4	[15.9; 24.9]	18.4	[15.8; 21.0]	10.3	[6.6; 14.0]
**Uganda**	2016	29.1	[25.2; 33.1]	33.2	[31.0; 35.3]	30.6	[28.3; 33.0]	34.1	[31.7; 36.5]	32.8	[28.4; 37.2]	32.8	[30.5; 35.0]	31.0	[27.8; 34.2]
**Zimbabwe**	2014	35.8	[32.5; 39.2]	31.3	[29.1; 33.4]	29.5	[27.1; 31.8]	35.4	[33.0; 37.9]	22.5	[16.5; 28.4]	30.3	[27.5; 33.1]	34.7	[32.3; 37.0]
**Middle East & North Africa**															
**Algeria**	2012	30.7	[28.4; 33.1]	27.4	[24.3; 30.5]	25.9	[23.4; 28.3]	32.9	[30.4; 35.4]	28.3	[24.9; 31.8]	30.0	[26.2; 33.9]	29.7	[27.4; 32.0]
**Iraq**	2011	22.3	[20.6; 23.9]	22.3	[20.3; 24.2]	17.4	[15.9; 19.0]	26.9	[25.1; 28.6]	23.9	[21.5; 26.3]	22.3	[20.6; 23.9]	21.2	[19.0; 23.4]
**Jordan**	2012	29.2	[25.3; 33.2]	27.7	[24.1; 31.3]	26.2	[22.6; 29.9]	31.7	[27.3; 36.0]	20.6	[10.0; 31.2]	37.6	[27.4; 47.8]	28.6	[25.2; 31.9]
**State of Palestine**	2014	28.8	[26.8; 30.7]	25.0	[21.3; 28.8]	22.9	[20.7; 25.1]	33.1	[30.8; 35.5]	11.7	[0.0; 25.6]	31.1	[28.1; 34.2]	26.8	[24.7; 28.9]
**Tunisia**	2011	23.2	[19.3; 27.0]	26.4	[21.8; 31.1]	21.7	[17.8; 25.6]	26.8	[22.6; 31.0]	28.5	[21.6; 35.3]	27.2	[22.0; 32.3]	21.0	[16.7; 25.2]
**Europe & Central Asia**															
**Belarus**	2012	9.7	[7.4; 12.0]	11.6	[7.1; 16.1]	8.6	[6.0; 11.1]	11.9	[8.7; 15.2]	NA	-	15.7	[0.0; 31.7]	10.0	[8.0; 12.1]
**Bosnia and Herzegovina**	2011	3.2	[1.3; 5.1]	5.3	[2.2; 8.3]	2.1	[1.0; 3.3]	7.1	[3.0; 11.2]	0.0	-	2.8	[0.9; 4.7]	5.2	[2.5; 7.9]
**Kazakhstan**	2015	18.3	[15.4; 21.1]	15.7	[11.7; 19.7]	15.5	[11.5; 19.4]	18.4	[15.1; 21.7]	0.0	-	2500.0	-	17.0	[14.5; 19.6]
**Kosovo**	2013	14.7	[9.9; 19.5]	16.0	[12.2; 19.7]	11.4	[7.7; 15.0]	19.2	[14.6; 23.9]	4.7	[0.0; 14.1]	30.3	[16.0; 44.5]	14.7	[11.7; 17.7]
**Kyrgyzstan**	2014	18.9	[14.5; 23.3]	15.0	[12.7; 17.3]	15.5	[12.8; 18.2]	16.6	[13.8; 19.4]	0.0	-	19.6	[0.0; 49.5]	15.8	[13.5; 18.1]
**Macedonia**	2011	10.9	[6.8; 14.9]	6.6	[3.0; 10.2]	7.9	[4.5; 11.3]	9.5	[5.4; 13.6]	0.0	-	10.2	[5.1; 15.2]	8.2	[4.8; 11.5]
**Moldova**	2012	19.7	[15.4; 24.1]	20.8	[15.9; 25.6]	18.1	[13.6; 22.6]	22.4	[17.5; 27.3]	NA	-	0.0	-	20.0	[16.4; 23.5]

**Montenegro**	2013	5.4	[2.2; 8.5]	4.5	[1.7; 7.3]	3.4	[1.2; 5.6]	6.3	[2.6; 10.1]	0.0	-	7.4	[0.8; 14.0]	4.7	[2.3; 7.1]
**Turkmenistan**	2015	6.0	[3.6; 8.3]	5.1	[3.4; 6.8]	5.2	[3.3; 7.1]	5.6	[3.7; 7.4]	NA	-	0.0	-	5.4	[3.9; 6.8]
**Ukraine**	2012	16.2	[12.9; 19.4]	15.7	[11.2; 20.1]	15.6	[12.3; 19.0]	16.4	[12.9; 19.8]	NA	-	NA	-	16.0	[13.3; 18.6]
**South Asia**															
**Bangladesh**	2012	26.5	[23.1; 29.9]	32.6	[31.1; 34.0]	28.3	[26.5; 30.1]	34.2	[32.4; 36.0]	34.8	[32.5; 37.2]	31.9	[29.7; 34.0]	28.9	[27.0; 30.7]
**Bhutan**	2010	28.2	[23.4; 32.9]	30.2	[27.2; 33.2]	26.8	[23.5; 30.1]	32.3	[28.9; 35.6]	28.6	[25.8; 31.4]	33.6	[27.1; 40.2]	30.8	[25.3; 36.3]
**Nepal**	2014	20.9	[15.8; 26.0]	32.9	[30.1; 35.8]	28.5	[24.9; 32.1]	34.1	[30.6; 37.7]	32.5	[29.0; 36.1]	30.7	[24.8; 36.7]	30.0	[25.6; 34.4]
**East Asia & the Pacific**															
**Cambodia**	2014	24.9	[20.9; 28.9]	25.7	[23.2; 28.2]	25.4	[22.5; 28.3]	25.8	[22.7; 28.8]	23.5	[18.6; 28.3]	25.4	[22.5; 28.2]	27.1	[22.9; 31.3]
**Lao**	2011	12.9	[10.0; 15.7]	15.2	[13.6; 16.8]	13.3	[11.6; 15.1]	16.1	[14.2; 17.9]	17.3	[14.9; 19.8]	13.9	[12.0; 15.7]	12.3	[10.0; 14.7]
**Mongolia**	2013	24.3	[21.6; 26.9]	23.1	[20.3; 25.9]	20.6	[18.2; 23.0]	27.0	[24.0; 29.9]	14.8	[9.2; 20.4]	21.2	[14.9; 27.4]	24.7	[22.5; 26.8]
**Thailand**	2015	18.9	[14.5; 23.3]	20.8	[17.7; 23.8]	16.9	[13.9; 19.8]	23.0	[19.2; 26.8]	10.8	[5.2; 16.3]	22.9	[18.7; 27.1]	21.5	[17.4; 25.7]
**Vietnam**	2013	7.5	[4.8; 10.2]	7.7	[5.1; 10.3]	6.7	[4.3; 9.1]	8.6	[5.5; 11.7]	7.0	[1.5; 12.4]	12.1	[6.9; 17.4]	6.6	[4.3; 8.9]
**Latin America & Caribbean**															
**Argentina**	2011	18.2	[16.2; 20.3]	NA	-	16.5	[13.5; 19.5]	19.8	[16.7; 22.9]	32.2	[7.3; 57.1]	26.5	[20.9; 32.1]	15.2	[13.1; 17.3]
**Belize**	2015	17.1	[13.0; 21.1]	25.9	[21.4; 30.3]	20.4	[16.3; 24.5]	24.6	[20.1; 29.1]	19.3	[5.6; 33.1]	24.0	[19.5; 28.5]	23.2	[17.8; 28.6]
**Costa Rica**	2011	18.7	[12.2; 25.3]	22.6	[17.0; 28.1]	18.6	[12.3; 25.0]	22.2	[15.5; 28.9]	40.5	[11.3; 69.7]	27.8	[20.9; 34.7]	15.6	[10.3; 20.9]
**Dominican Republic**	2014	14.7	[13.1; 16.3]	15.5	[13.4; 17.7]	13.6	[11.8; 15.5]	16.2	[14.3; 18.1]	16.7	[11.0; 22.5]	17.6	[15.2; 20.0]	13.5	[11.9; 15.1]
**El Salvador**	2014	20.4	[17.9; 23.0]	17.8	[15.3; 20.2]	16.9	[14.4; 19.4]	21.7	[19.0; 24.3]	17.8	[11.7; 24.0]	22.0	[18.8; 25.1]	17.8	[15.5; 20.1]
**Guyana**	2014	22.0	[16.1; 27.9]	26.0	[22.6; 29.3]	22.6	[18.9; 26.3]	27.0	[22.4; 31.6]	14.4	[4.9; 24.0]	27.0	[20.9; 33.1]	24.7	[21.4; 28.1]
**Jamaica**	2011	21.5	[16.5; 26.4]	21.0	[13.7; 28.3]	17.3	[12.2; 22.5]	24.6	[18.9; 30.3]			16.5	[2.8; 30.1]	21.5	[17.3; 25.7]
**Mexico**	2015	21.0	[17.4; 24.6]	21.4	[18.1; 24.6]	18.0	[13.8; 22.2]	24.7	[21.8; 27.6]	19.6	[8.0; 31.2]	22.0	[17.8; 26.2]	20.9	[17.5; 24.2]
**Panama**	2013	18.5	[13.2; 23.7]	18.7	[15.0; 22.4]	17.0	[13.2; 20.9]	19.8	[15.0; 24.6]	21.2	[15.4; 26.9]	21.2	[16.0; 26.5]	17.4	[13.1; 21.6]
**Paraguay**	2016	19.2	[15.9; 22.6]	16.0	[13.0; 18.9]	16.4	[13.3; 19.5]	19.4	[16.0; 22.8]	9.7	[0.0; 20.3]	21.9	[18.0; 25.9]	21.4	[15.6; 27.3]
**Suriname**	2010	28.2	[23.2; 33.2]	39.1	[35.3; 42.9]	28.3	[24.3; 32.3]	37.5	[32.6; 42.3]	40.5	[33.8; 47.2]	35.3	[30.6; 40.1]	28.3	[23.5; 33.1]
**Trinidad and Tobago**	2011	22.9	[16.5; 29.3]	16.6	[11.8; 21.4]	19.5	[14.0; 25.0]	20.8	[14.9; 26.6]	62.9	[12.8; 100.0]	28.0	[15.5; 40.5]	18.2	[13.6; 22.8]
**Uruguay**	2012	20.2	[12.2; 28.2]	4.1	[0.0; 9.7]	22.6	[10.3; 34.8]	17.5	[10.0; 25.0]	NA	-	23.2	[5.0; 41.5]	18.4	[10.6; 26.3]

Note: Data not available marked as NA

Table S9. Prevalence of suspected developmental delay in each studied country for the learning domain by urban/rural residence, sex of the child and maternal education; countries grouped by world region. Source: MICS and DHS surveys, 2010-2016.

		**Area (%)**				**Sex (%)**				**Maternal education (%)**					
**Country**	**Year**	**Urban**	**95% IC**	**Rural**	**95% IC**	**Female**	**95% IC**	**Male**	**95% IC**	**None**	**95% IC**	**Primary**	**95% IC**	**Secondary +**	**95% IC**
**West & Central Africa**															
**Benin**	2014	15.6	[13.4; 17.8]	18.6	[16.3; 20.9]	16.2	[14.2; 18.2]	18.4	[16.4; 20.4]	18.2	[16.2; 20.2]	16.3	[13.5; 19.1]	15.2	[11.0; 19.5]
**Cameroon**	2014	11.5	[9.2; 13.9]	15.1	[12.7; 17.5]	13.1	[10.7; 15.5]	14.1	[11.7; 16.6]	14.9	[11.8; 17.9]	14.6	[12.0; 17.2]	10.9	[8.1; 13.7]
**CAR**	2010	19.6	[16.1; 23.0]	24.5	[21.9; 27.1]	20.6	[18.2; 23.0]	25.0	[22.1; 27.8]	26.0	[22.8; 29.1]	22.4	[19.5; 25.3]	14.9	[10.7; 19.1]
**Chad**	2014	38.0	[33.0; 43.1]	47.0	[43.7; 50.4]	43.8	[40.6; 47.0]	47.0	[42.9; 51.1]	46.9	[43.9; 50.0]	44.6	[39.0; 50.2]	34.1	[27.6; 40.6]
**Congo Brazzaville**	2014	12.0	[9.5; 14.4]	19.8	[16.7; 23.0]	14.5	[12.0; 17.0]	15.2	[12.8; 17.6]	22.3	[15.9; 28.8]	17.5	[14.0; 21.0]	14.1	[11.6; 16.7]
**Congo DR**	2013	13.8	[10.9; 16.7]	21.5	[17.7; 25.4]	18.4	[14.9; 21.8]	20.0	[16.6; 23.4]	24.8	[19.2; 30.4]	20.2	[16.1; 24.3]	14.6	[11.4; 17.8]
**Côte D'Ivoire**	2016	7.8	[5.7; 10.0]	12.9	[9.9; 15.9]	10.7	[8.4; 12.9]	11.2	[8.6; 13.9]	12.4	[9.9; 14.9]	9.7	[6.6; 12.8]	6.2	[3.2; 9.3]
**Gambia**	2010	3.6	[2.2; 5.0]	5.5	[3.9; 7.1]	3.8	[2.6; 4.9]	5.6	[4.3; 7.0]	5.1	[3.9; 6.2]	5.1	[2.2; 7.9]	2.7	[1.2; 4.3]
**Ghana**	2011	8.9	[5.9; 11.9]	11.8	[9.3; 14.3]	11.4	[8.4; 14.3]	9.7	[7.6; 11.9]	12.6	[9.9; 15.4]	8.4	[5.2; 11.7]	9.8	[6.5; 13.0]
**Guinea**	2016	14.6	[11.0; 18.2]	21.2	[18.3; 24.1]	19.5	[16.8; 22.3]	18.6	[15.7; 21.4]	20.7	[18.1; 23.2]	14.9	[10.1; 19.6]	13.1	[9.1; 17.0]
**Guinea Bissau**	2014	12.5	[9.6; 15.4]	10.8	[8.9; 12.8]	9.3	[7.6; 10.9]	13.7	[11.3; 16.0]	11.1	[9.1; 13.1]	11.0	[8.1; 13.8]	13.7	[9.4; 17.9]
**Mali**	2015	9.9	[7.5; 12.3]	13.9	[12.2; 15.5]	12.7	[10.9; 14.4]	13.4	[11.8; 15.1]	14.3	[12.6; 15.9]	8.4	[6.1; 10.8]	9.9	[6.4; 13.4]
**Mauritania**	2015	16.8	[13.9; 19.8]	19.9	[16.8; 23.0]	17.5	[15.0; 20.1]	19.7	[17.0; 22.4]	22.2	[18.2; 26.3]	20.2	[16.6; 23.8]	17.1	[14.2; 20.1]
**Nigeria**	2016	14.6	[12.2; 17.0]	24.1	[22.3; 25.9]	20.7	[18.8; 22.5]	21.6	[19.8; 23.4]	28.3	[25.4; 31.1]	21.6	[19.0; 24.1]	13.8	[12.1; 15.6]
**S Tome and Principe**	2014	19.9	[15.6; 24.2]	19.3	[14.7; 24.0]	16.9	[12.6; 21.2]	22.5	[17.9; 27.0]	16.3	[5.6; 27.1]	20.4	[16.5; 24.4]	18.7	[12.1; 25.3]
**Sierra Leone**	2010	21.1	[17.0; 25.2]	22.7	[20.3; 25.0]	23.7	[21.0; 26.4]	20.8	[18.4; 23.2]	23.2	[21.0; 25.5]	19.5	[14.7; 24.3]	18.5	[13.8; 23.3]
**Togo**	2013	26.7	[22.8; 30.6]	29.6	[25.8; 33.4]	25.5	[22.3; 28.8]	31.7	[28.0; 35.4]	30.6	[26.5; 34.8]	26.9	[22.9; 30.9]	27.2	[22.1; 32.2]
**Eastern & Southern Africa**															
**Burundi**	2016	21.0	[14.9; 27.0]	37.3	[35.0; 39.5]	34.0	[31.4; 36.5]	37.8	[35.0; 40.5]	39.2	[36.4; 42.0]	35.4	[32.4; 38.3]	19.5	[15.4; 23.7]
**Eswatini**	2014	1.4	[-0.2; 2.9]	5.9	[3.6; 8.1]	3.3	[1.7; 5.0]	6.5	[3.9; 9.1]	6.7	[2.2; 11.1]	5.2	[2.4; 8.0]	4.4	[2.5; 6.4]
**Malawi**	2013	12.2	[8.6; 15.8]	19.6	[18.0; 21.3]	18.4	[16.5; 20.4]	19.1	[17.2; 21.0]	25.1	[21.4; 28.7]	18.8	[17.1; 20.5]	12.2	[9.4; 14.9]
**Rwanda**	2014	8.8	[5.5; 12.2]	14.0	[12.2; 15.8]	12.8	[10.6; 15.0]	13.6	[11.5; 15.6]	17.7	[13.8; 21.6]	13.2	[11.3; 15.1]	5.6	[2.9; 8.4]
**Uganda**	2016	9.6	[7.1; 12.0]	14.2	[12.6; 15.9]	13.7	[11.9; 15.4]	12.9	[11.0; 14.8]	14.0	[11.0; 16.9]	15.0	[13.0; 16.9]	8.8	[6.7; 10.9]
**Zimbabwe**	2014	6.4	[4.9; 8.0]	11.4	[9.8; 13.0]	10.0	[8.5; 11.6]	10.3	[8.5; 12.0]	15.6	[9.1; 22.1]	10.9	[9.0; 12.8]	9.2	[7.8; 10.6]
**Middle East & North Africa**															
**Algeria**	2012	9.6	[8.2; 11.0]	10.1	[8.0; 12.1]	9.4	[8.0; 10.8]	10.1	[8.7; 11.6]	12.4	[10.2; 14.7]	8.1	[6.1; 10.0]	9.5	[8.0; 10.9]
**Iraq**	2011	9.5	[8.3; 10.6]	12.1	[10.7; 13.5]	9.4	[8.3; 10.4]	11.3	[10.1; 12.5]	13.2	[11.3; 15.1]	10.9	[9.8; 12.1]	7.8	[6.5; 9.1]
**Jordan**	2012	9.2	[7.3; 11.1]	9.4	[7.2; 11.7]	7.1	[5.3; 8.8]	11.3	[8.4; 14.2]	12.7	[5.8; 19.6]	10.7	[6.0; 15.3]	9.0	[7.2; 10.8]
**State of Palestine**	2014	7.1	[5.9; 8.4]	7.4	[4.8; 10.0]	6.5	[5.3; 7.8]	7.7	[6.2; 9.2]	11.3	[0.0; 25.9]	8.7	[6.7; 10.7]	6.3	[5.2; 7.5]
**Tunisia**	2011	6.4	[4.1; 8.7]	6.2	[3.6; 8.8]	5.6	[3.1; 8.1]	7.1	[4.8; 9.3]	8.7	[3.9; 13.6]	6.2	[3.6; 8.8]	5.8	[3.3; 8.3]
**Europe & Central Asia**															
**Belarus**	2012	0.0	-	0.0	-	0.0	-	0.0	-	NA	-	0.0	-	0.0	[0.0; 0.1]
**Bosnia and Herzegovina**	2011	1.0	[0.0; 2.0]	0.8	[0.1; 1.6]	0.5	[0.0; 1.1]	1.2	[0.2; 2.3]	0.0	-	1.0	[0.0; 2.2]	0.8	[0.1; 1.5]
**Kazakhstan**	2015	2.1	[0.9; 3.2]	1.6	[0.6; 2.5]	1.7	[0.9; 2.6]	1.9	[0.7; 3.1]	0.0	-	25.0	-	1.8	[0.9; 2.8]
**Kosovo**	2013	3.5	[0.6; 6.5]	2.7	[0.9; 4.5]	2.2	[0.6; 3.7]	3.8	[1.0; 6.5]	0.0	-	4.6	[0.0; 11.1]	3.0	[1.1; 4.9]
**Kyrgyzstan**	2014	5.5	[3.3; 7.7]	6.4	[4.0; 8.8]	5.9	[3.5; 8.4]	6.4	[4.0; 8.8]	0.0	-	14.6	[0.0; 40.2]	5.8	[3.8; 7.9]
**Macedonia**	2011	1.1	[0.0; 2.4]	1.7	[0.1; 3.4]	1.8	[0.0; 3.6]	1.0	[0.0; 2.2]	21.2	[0.0; 49.8]	2.2	[0.3; 4.1]	0.3	[0.0; 0.9]

**Moldova**	2012	0.1	[0.0; 0.6]	0.5	[0.0; 1.3]	0.5	[0.0; 1.0]	0.2	[0.0; 1.0]	NA	-	0.0	-	0.1	[0.0; 0.6]
**Montenegro**	2013	1.0	[0.0; 2.4]	1.1	[0.0; 2.9]	0.8	[0.0; 2.4]	1.2	[0.0; 2.5]	0.0	-	3.0	[0.0; 9.0]	0.6	[0.0; 1.4]
**Turkmenistan**	2015	1.7	[0.4; 3.1]	3.1	[1.1; 5.1]	3.1	[1.0; 5.1]	2.2	[0.7; 3.7]	NA	-	0.0	-	2.6	[1.0; 4.3]
**Ukraine**	2012	1.1	[0.5; 1.8]	1.8	[0.5; 3.0]	1.0	[0.3; 1.7]	1.6	[0.7; 2.6]	NA	-	NA	-	1.3	[0.6; 2.0]
**South Asia**															
**Bangladesh**	2012	11.4	[8.7; 14.1]	12.2	[11.1; 13.3]	12.3	[10.8; 13.8]	11.8	[10.4; 13.2]	14.8	[12.8; 16.8]	12.8	[11.0; 14.7]	9.8	[8.4; 11.2]
**Bhutan**	2010	5.7	[3.6; 7.8]	6.2	[4.6; 7.8]	5.2	[3.4; 7.1]	6.8	[5.2; 8.4]	6.4	[4.9; 7.9]	8.4	[3.2; 13.5]	3.1	[1.5; 4.8]
**Nepal**	2014	9.7	[5.9; 13.6]	19.1	[15.5; 22.7]	17.1	[13.0; 21.2]	18.5	[15.1; 22.0]	22.0	[17.1; 26.9]	17.7	[12.8; 22.5]	12.1	[8.9; 15.2]
**East Asia & the Pacific**															
**Cambodia**	2014	4.6	[2.8; 6.5]	11.1	[9.3; 12.8]	10.2	[8.0; 12.3]	10.2	[8.3; 12.2]	11.6	[7.3; 15.9]	11.1	[9.1; 13.0]	7.9	[5.8; 10.0]
**Lao**	2011	3.2	[1.1; 5.3]	6.8	[5.4; 8.2]	6.1	[4.7; 7.4]	6.1	[4.6; 7.5]	10.3	[7.7; 12.9]	4.2	[3.1; 5.3]	2.9	[1.6; 4.2]
**Mongolia**	2013	1.5	[0.7; 2.2]	2.1	[1.1; 3.1]	1.3	[0.6; 2.0]	2.1	[1.2; 3.1]	3.1	[0.2; 6.1]	4.9	[2.1; 7.7]	1.3	[0.7; 2.0]
**Thailand**	2015	0.4	[0.0; 0.9]	0.4	[0.0; 0.9]	0.4	[0.0; 0.9]	0.4	[0.0; 0.9]	3.3	[0.0; 8.5]	0.3	[0.0; 0.8]	0.4	[0.0; 0.9]
**Vietnam**	2013	3.6	[1.9; 5.4]	5.2	[3.3; 7.2]	4.8	[2.8; 6.7]	4.7	[2.7; 6.8]	18.6	[9.7; 27.4]	4.6	[2.1; 7.1]	3.6	[2.1; 5.0]
**Latin America & Caribbean**															
**Argentina**	2011	2.1	[1.2; 3.0]	NA	-	2.2	[1.0; 3.3]	2.0	[0.8; 3.2]	13.3	[0.0; 37.4]	3.1	[0.8; 5.5]	1.6	[0.8; 2.3]
**Belize**	2015	4.2	[1.8; 6.6]	5.1	[2.2; 8.1]	4.1	[1.9; 6.3]	5.5	[2.3; 8.6]	7.9	[0.0; 17.0]	5.5	[1.8; 9.3]	4.0	[1.7; 6.3]
**Costa Rica**	2011	0.0	-	0.9	[0.0; 1.8]	0.6	[0.0; 1.4]	0.2	[0.0; 0.4]	4.5	[0.0; 10.7]	0.8	[0.0; 1.9]	0.0	[0.0; 0.1]
**Dominican Republic**	2014	1.9	[1.3; 2.5]	2.4	[1.3; 3.5]	2.0	[1.2; 2.9]	2.1	[1.4; 2.7]	5.6	[1.7; 9.5]	2.1	[1.2; 3.1]	1.7	[1.2; 2.3]
**El Salvador**	2014	1.7	[1.0; 2.5]	3.2	[1.6; 4.8]	2.5	[1.2; 3.8]	2.3	[1.3; 3.2]	5.3	[1.7; 8.9]	3.0	[1.6; 4.4]	1.6	[0.8; 2.3]
**Guyana**	2014	2.7	[1.0; 4.4]	3.7	[2.0; 5.5]	2.6	[1.1; 4.0]	4.3	[2.1; 6.5]	4.9	[0.0; 11.1]	6.6	[2.6; 10.7]	2.9	[1.6; 4.1]
**Jamaica**	2011	2.3	[0.7; 4.0]	3.2	[0.8; 5.6]	2.4	[0.7; 4.1]	3.0	[0.8; 5.2]	NA	-	4.8	[0.0; 14.3]	2.6	[1.2; 4.1]
**Mexico**	2015	1.1	[0.5; 1.8]	1.7	[0.8; 2.5]	0.8	[0.4; 1.2]	1.8	[0.8; 2.9]	3.1	[0.4; 5.8]	1.7	[0.6; 2.9]	1.1	[0.6; 1.7]
**Panama**	2013	2.7	[1.0; 4.3]	5.3	[2.9; 7.7]	4.7	[2.4; 7.1]	2.8	[1.1; 4.5]	10.6	[5.8; 15.5]	3.6	[2.0; 5.3]	3.2	[1.3; 5.1]
**Paraguay**	2016	3.0	[1.5; 4.4]	3.2	[1.0; 5.4]	3.4	[1.4; 5.4]	2.7	[1.2; 4.2]	11.7	[0.0; 25.6]	5.1	[2.4; 7.7]	4.5	[1.3; 7.7]
**Suriname**	2010	2.5	[0.9; 4.1]	3.2	[2.1; 4.3]	2.8	[1.2; 4.4]	2.8	[1.4; 4.1]	5.6	[3.0; 8.2]	2.4	[0.8; 3.9]	2.3	[0.7; 3.9]
**Trinidad and Tobago**	2011	3.1	[0.3; 6.0]	2.7	[0.5; 4.9]	1.8	[0.0; 3.8]	4.2	[1.5; 7.0]	0.0	-	3.3	[0.0; 7.3]	2.9	[1.0; 4.9]
**Uruguay**	2012	1.8	[0.0; 3.8]	1.6	[0.0; 4.8]	2.9	[0.0; 7.3]	0.9	[0.0; 1.8]	NA	-	1.7	[0.0; 4.0]	1.8	[0.0; 4.4]

Note: Data not available marked as NA

Table S10. Prevalence of suspected developmental delay in each studied country for the literacy-numeracy domain by urban/rural residence, sex of the child and maternal education; countries grouped by world region. Source: MICS and DHS surveys, 2010-2016.

		Area (%)				Sex (%)				Maternal education (%)					
**Country**	**Year**	**Urban**	**95% IC**	**Rural**	**95% IC**	**Female**	**95% IC**	**Male**	**95% IC**	**None**	**95% IC**	**Primary**	**95% IC**	**Secondary +**	**95% IC**
							**West & Central Africa**								
**Benin**	2014	86.4	[84.1; 88.8]	97.0	[96.1; 97.9]	92.6	[90.9; 94.3]	92.3	[90.7; 93.8]	96.7	[95.9; 97.5]	87.7	[84.5; 90.9]	78.7	[72.9; 84.4]
**Cameroon**	2014	73.3	[69.2; 77.3]	91.2	[89.1; 93.4]	82.1	[79.1; 85.1]	85.6	[82.9; 88.3]	97.7	[96.5; 98.9]	83.7	[80.6; 86.7]	67.3	[63.5; 71.0]
**CAR**	2010	86.8	[83.5; 90.2]	96.0	[94.8; 97.1]	92.6	[90.7; 94.4]	92.8	[90.8; 94.9]	96.5	[95.0; 97.9]	93.2	[91.4; 95.0]	81.4	[76.6; 86.1]
**Chad**	2014	88.2	[85.2; 91.3]	96.0	[95.0; 97.0]	94.4	[92.9; 95.8]	94.8	[93.6; 96.0]	95.6	[94.5; 96.7]	94.3	[92.3; 96.2]	86.8	[82.3; 91.3]
**Congo Brazzaville**	2014	82.0	[79.3; 84.8]	93.6	[92.2; 95.1]	84.7	[81.9; 87.5]	87.8	[85.1; 90.6]	96.4	[94.3; 98.6]	94.1	[91.7; 96.4]	85.9	[83.3; 88.4]
**Congo DR**	2013	84.2	[80.5; 87.9]	91.4	[89.0; 93.8]	89.3	[86.6; 91.9]	89.2	[86.8; 91.5]	93.8	[91.0; 96.6]	91.7	[89.3; 94.0]	83.4	[79.7; 87.2]
**Côte D'Ivoire**	2016	83.9	[79.8; 88.0]	97.7	[96.8; 98.6]	91.5	[88.8; 94.2]	93.4	[91.5; 95.3]	97.2	[96.1; 98.3]	90.2	[86.6; 93.8]	72.3	[64.3; 80.3]
**Gambia**	2010	83.4	[79.2; 87.5]	90.9	[88.6; 93.2]	87.1	[84.0; 90.2]	88.2	[85.7; 90.7]	91.8	[89.9; 93.7]	84.7	[78.9; 90.6]	69.2	[62.0; 76.4]
**Ghana**	2011	57.5	[52.2; 62.7]	82.1	[79.2; 85.0]	68.7	[64.7; 72.7]	74.4	[70.5; 78.3]	88.3	[85.8; 90.8]	75.3	[69.4; 81.1]	55.0	[49.8; 60.2]
**Guinea**	2016	85.6	[82.1; 89.0]	98.2	[97.4; 99.0]	94.7	[93.0; 96.3]	93.7	[92.1; 95.3]	96.8	[95.8; 97.7]	89.6	[85.6; 93.6]	82.6	[77.6; 87.5]
**Guinea Bissau**	2014	81.8	[77.7; 86.0]	98.5	[98.0; 99.0]	91.2	[88.9; 93.5]	93.7	[91.6; 95.8]	98.0	[97.2; 98.8]	91.5	[88.8; 94.2]	70.3	[63.5; 77.0]
**Mali**	2015	84.2	[81.1; 87.3]	93.2	[91.9; 94.5]	91.8	[90.4; 93.1]	91.2	[89.6; 92.7]	93.6	[92.3; 94.9]	89.4	[86.2; 92.5]	84.4	[80.1; 88.6]
**Mauritania**	2015	67.4	[63.8; 71.0]	76.4	[73.5; 79.3]	72.0	[69.0; 75.0]	73.1	[70.2; 76.0]	78.1	[74.6; 81.7]	73.5	[69.5; 77.4]	72.4	[69.1; 75.7]
**Nigeria**	2016	48.6	[44.2; 53.0]	79.9	[78.1; 81.6]	69.1	[66.8; 71.4]	71.5	[69.4; 73.5]	90.7	[89.3; 92.1]	68.3	[65.4; 71.1]	42.2	[39.3; 45.1]
**S Tome and Principe**	2014	81.8	[77.3; 86.3]	89.3	[85.2; 93.4]	83.7	[79.5; 87.9]	85.0	[80.6; 89.3]	80.6	[64.7; 96.6]	86.9	[83.1; 90.7]	79.4	[72.0; 86.8]
**Sierra Leone**	2010	83.8	[80.0; 87.5]	93.0	[91.6; 94.4]	90.4	[88.6; 92.2]	90.7	[88.9; 92.5]	93.4	[92.2; 94.6]	88.9	[83.9; 93.8]	72.9	[66.7; 79.1]
**Togo**	2013	84.1	[80.6; 87.6]	96.6	[95.3; 97.8]	92.4	[90.3; 94.5]	92.2	[90.1; 94.3]	97.1	[96.0; 98.2]	91.6	[88.9; 94.3]	83.1	[78.7; 87.6]
							**Eastern & Southern Africa**								
**Burundi**	2016	70.6	[65.8; 75.4]	93.2	[92.2; 94.2]	90.8	[89.3; 92.3]	91.8	[90.5; 93.2]	95.4	[94.4; 96.4]	90.6	[89.0; 92.3]	71.0	[66.1; 75.9]
**Eswatini**	2014	68.4	[60.7; 76.1]	86.3	[83.5; 89.1]	78.2	[73.9; 82.5]	87.1	[83.9; 90.3]	91.2	[85.6; 96.7]	91.4	[88.4; 94.5]	75.4	[71.2; 79.7]
**Malawi**	2013	62.9	[58.2; 67.5]	85.3	[83.9; 86.7]	79.4	[77.4; 81.5]	86.0	[84.3; 87.7]	91.1	[88.9; 93.3]	84.4	[82.8; 85.9]	65.9	[61.7; 70.1]
**Rwanda**	2014	82.8	[78.5; 87.1]	94.7	[93.5; 95.9]	92.0	[90.1; 94.0]	93.6	[92.3; 95.0]	97.5	[96.0; 99.1]	93.5	[92.1; 94.9]	80.4	[75.0; 85.8]
**Uganda**	2016	53.1	[47.9; 58.4]	75.6	[73.6; 77.6]	69.7	[67.1; 72.4]	72.4	[69.8; 74.9]	85.9	[82.4; 89.3]	76.6	[74.6; 78.7]	49.3	[45.4; 53.2]
**Zimbabwe**	2014	85.7	[83.3; 88.1]	93.3	[92.2; 94.3]	90.9	[89.5; 92.3]	91.9	[90.6; 93.2]	97.1	[94.7; 99.5]	94.7	[93.4; 95.9]	88.7	[87.1; 90.2]
							**Middle East & North Africa**								
**Algeria**	2012	65.6	[62.8; 68.4]	80.6	[77.6; 83.6]	69.6	[66.6; 72.5]	72.7	[70.2; 75.2]	86.2	[83.6; 88.8]	78.0	[74.5; 81.5]	64.3	[61.5; 67.1]
**Iraq**	2011	78.1	[75.9; 80.2]	89.5	[88.1; 90.9]	81.2	[79.3; 83.0]	82.8	[81.0; 84.7]	89.8	[88.2; 91.5]	84.7	[82.7; 86.6]	73.2	[70.3; 76.0]
**Jordan**	2012	82.4	[79.2; 85.5]	89.2	[86.9; 91.5]	82.5	[78.6; 86.3]	84.6	[81.6; 87.7]	95.9	[91.5; 100.0]	85.5	[78.2; 92.9]	83.0	[80.2; 85.9]
**State of Palestine**	2014	81.0	[79.2; 82.9]	81.9	[77.7; 86.0]	79.6	[77.3; 81.9]	82.6	[80.5; 84.7]	97.0	[90.6; 100.0]	86.8	[84.5; 89.0]	78.0	[75.9; 80.2]
**Tunisia**	2011	61.2	[56.1; 66.2]	78.7	[73.0; 84.5]	64.7	[59.3; 70.1]	70.2	[65.2; 75.2]	83.2	[74.8; 91.6]	70.9	[65.4; 76.3]	60.4	[55.0; 65.7]
							**Europe & Central Asia**								
**Belarus**	2012	47.5	[43.0; 51.9]	64.2	[57.1; 71.4]	45.2	[39.5; 50.8]	59.2	[54.0; 64.4]	NA	-	87.8	[79.1; 96.5]	50.8	[46.9; 54.7]
**Bosnia and Herzegovina**	2011	64.8	[56.4; 73.2]	79.7	[75.4; 84.1]	70.1	[64.0; 76.2]	79.1	[74.0; 84.1]	79.9	[56.3; 103.5]	81.8	[75.3; 88.3]	72.0	[67.2; 76.9]
**Kazakhstan**	2015	67.0	[63.2; 70.8]	78.3	[74.8; 81.8]	71.0	[67.0; 75.1]	74.6	[70.8; 78.4]	100.0	-	75.0	-	72.8	[69.9; 75.8]
**Kosovo**	2013	78.1	[70.4; 85.8]	85.2	[81.4; 88.9]	81.5	[76.2; 86.8]	83.5	[79.1; 88.0]	93.9	[81.7; 100.0]	93.5	[86.4; 100.0]	81.5	[77.3; 85.8]
**Kyrgyzstan**	2014	77.3	[72.5; 82.0]	89.4	[86.8; 91.9]	83.9	[80.6; 87.3]	88.1	[85.3; 91.0]	100.0	-	69.6	[7.1; 100.0]	85.2	[82.7; 87.8]
**Macedonia**	2011	53.6	[47.0; 60.2]	60.8	[52.8; 68.8]	57.6	[50.2; 65.0]	56.8	[49.2; 64.3]	74.1	[47.5; 100.0]	62.4	[53.7; 71.2]	53.3	[47.2; 59.3]
**Moldova**	2012	62.2	[56.5; 68.0]	75.8	[71.1; 80.6]	69.7	[63.8; 75.6]	71.7	[66.5; 76.9]	NA	-	94.0	[81.4; 100.0]	70.0	[66.1; 73.9]

**Montenegro**	2013	74.3	[68.1; 80.6]	86.7	[80.7; 92.7]	80.1	[74.0; 86.1]	77.8	[71.4; 84.2]	100.0	-	91.4	[85.1; 97.7]	76.3	[70.8; 81.8]
**Turkmenistan**	2015	81.9	[77.7; 86.2]	83.1	[78.9; 87.3]	83.2	[79.2; 87.3]	82.2	[78.7; 85.7]	NA	-	100.0	-	82.7	[79.4; 85.9]
**Ukraine**	2012	49.8	[43.9; 55.7]	64.6	[58.5; 70.7]	53.0	[46.7; 59.2]	55.7	[50.4; 60.9]	NA	-	NA	-	54.3	[49.7; 58.9]
**South Asia**															
**Bangladesh**	2012	66.6	[62.5; 70.8]	82.8	[81.6; 83.9]	79.1	[77.2; 81.0]	80.0	[78.3; 81.7]	91.0	[89.5; 92.6]	86.7	[85.0; 88.5]	67.2	[64.9; 69.5]
**Bhutan**	2010	61.9	[55.0; 68.9]	80.2	[77.6; 82.8]	73.8	[69.8; 77.7]	76.2	[72.8; 79.5]	82.6	[79.9; 85.3]	72.9	[65.8; 80.0]	46.7	[40.5; 52.9]
**Nepal**	2014	41.0	[34.3; 47.7]	76.4	[72.5; 80.3]	70.0	[65.1; 74.9]	73.3	[69.1; 77.5]	89.0	[86.3; 91.8]	74.8	[69.3; 80.3]	45.1	[39.9; 50.3]
**East Asia & the Pacific**															
**Cambodia**	2014	57.8	[52.6; 63.1]	75.2	[72.1; 78.2]	72.3	[68.5; 76.1]	73.5	[70.1; 76.8]	87.4	[83.4; 91.5]	76.2	[72.8; 79.5]	59.1	[54.6; 63.6]
**Lao**	2011	56.3	[50.6; 62.0]	86.0	[84.3; 87.8]	78.3	[75.7; 80.8]	81.9	[79.7; 84.2]	93.1	[91.4; 94.8]	83.0	[80.8; 85.2]	55.3	[50.9; 59.6]
**Mongolia**	2013	90.0	[88.1; 91.9]	93.6	[91.9; 95.3]	89.9	[88.0; 91.8]	93.0	[91.4; 94.7]	93.4	[88.8; 98.1]	94.6	[91.1; 98.0]	91.1	[89.6; 92.5]
**Thailand**	2015	26.5	[21.8; 31.3]	29.1	[25.5; 32.7]	26.6	[22.8; 30.3]	29.5	[25.5; 33.4]	38.9	[24.9; 53.0]	31.5	[26.5; 36.6]	28.3	[24.0; 32.5]
**Vietnam**	2013	68.0	[62.5; 73.4]	73.6	[69.4; 77.7]	72.5	[68.1; 77.0]	71.3	[66.7; 76.0]	94.8	[89.5; 100.0]	81.5	[75.8; 87.2]	67.4	[63.5; 71.4]
**Latin America & Caribbean**															
**Argentina**	2011	58.4	[55.5; 61.3]	NA	-	54.0	[50.2; 57.9]	62.5	[58.5; 66.4]	79.9	[60.0; 99.7]	60.6	[54.8; 66.5]	57.5	[54.3; 60.7]
**Belize**	2015	35.9	[29.9; 41.9]	52.3	[46.0; 58.7]	39.9	[34.6; 45.2]	52.1	[45.7; 58.6]	65.3	[48.2; 82.3]	54.5	[47.6; 61.4]	36.6	[29.5; 43.8]
**Costa Rica**	2011	66.5	[57.2; 75.7]	84.2	[78.9; 89.6]	75.9	[66.6; 85.2]	72.7	[64.4; 81.1]	96.0	[88.8; 100.0]	86.0	[80.7; 91.3]	66.9	[57.9; 75.8]
**Dominican Republic**	2014	76.8	[74.8; 78.8]	87.3	[85.4; 89.2]	78.6	[76.5; 80.8]	80.1	[78.0; 82.2]	95.0	[91.3; 98.7]	86.3	[83.5; 89.2]	75.0	[73.1; 76.9]
**El Salvador**	2014	75.9	[72.5; 79.3]	89.0	[86.8; 91.1]	81.6	[78.5; 84.7]	81.6	[79.0; 84.2]	94.3	[91.5; 97.1]	85.8	[83.0; 88.7]	77.2	[74.0; 80.3]
**Guyana**	2014	27.7	[21.4; 34.0]	35.4	[31.6; 39.2]	29.4	[25.3; 33.4]	37.0	[32.1; 42.0]	68.8	[53.6; 84.0]	48.2	[40.4; 56.0]	29.6	[26.0; 33.2]
**Jamaica**	2011	26.7	[18.9; 34.5]	42.4	[35.4; 49.4]	29.5	[22.6; 36.4]	37.0	[29.4; 44.6]			59.0	[37.8; 80.1]	32.4	[26.6; 38.2]
**Mexico**	2015	77.1	[72.5; 81.7]	81.3	[76.8; 85.7]	78.6	[72.9; 84.3]	77.7	[73.9; 81.4]	90.3	[84.1; 96.5]	82.7	[78.0; 87.5]	76.6	[72.2; 81.1]
**Panama**	2013	76.9	[71.9; 81.9]	87.9	[83.8; 91.9]	84.4	[80.2; 88.6]	78.5	[73.9; 83.2]	96.4	[93.7; 99.0]	89.4	[85.8; 92.9]	76.9	[72.2; 81.6]
**Paraguay**	2016	70.7	[66.6; 74.8]	86.7	[82.9; 90.6]	76.3	[72.1; 80.6]	78.1	[74.1; 82.0]	95.8	[90.5; 100.0]	87.1	[83.7; 90.5]	83.6	[77.5; 89.7]
**Suriname**	2010	72.8	[67.6; 78.0]	88.6	[85.9; 91.3]	73.7	[68.9; 78.6]	85.4	[81.5; 89.3]	94.1	[90.3; 97.8]	84.5	[80.0; 89.0]	71.3	[66.4; 76.1]
**Trinidad and Tobago**	2011	18.4	[12.4; 24.5]	15.8	[11.1; 20.4]	15.9	[10.7; 21.0]	18.8	[13.1; 24.4]	11.8	[0.0; 39.7]	17.1	[9.3; 24.9]	17.2	[12.4; 21.9]
**Uruguay**	2012	49.9	[40.4; 59.5]	55.6	[37.8; 73.4]	56.7	[45.3; 68.0]	44.9	[32.4; 57.3]	NA	-	66.2	[46.3; 86.1]	44.2	[35.9; 52.5]

Note: Data not available marked as NA

Table S11. Prevalence of suspected developmental delay in each studied country for the combined indicator (ECDI) domain by urban/rural residence, sex of the child and maternal education; countries grouped by world region. Source: MICS and DHS surveys, 2010-2016.

Country	Year	Area (%)				Sex (%)				Maternal education (%)					
		Urban	95% IC	Rural	95% IC	Female	95% IC	Male	95% IC	None	95% IC	Primary	95% IC	Secondary +	95% IC
**West & Central Africa**															
**Benin**	2014	34.4	[31.7; 37.1]	40.3	[37.5; 43.2]	34.0	[31.4; 36.6]	41.7	[39.1; 44.3]	40.2	[37.8; 42.5]	34.3	[30.5; 38.1]	32.7	[27.2; 38.3]
**Cameroon**	2014	30.0	[26.9; 33.0]	41.4	[38.4; 44.5]	34.3	[30.9; 37.6]	39.0	[36.0; 42.0]	39.0	[35.2; 42.8]	39.9	[36.4; 43.4]	29.9	[26.0; 33.8]
**CAR**	2010	50.6	[45.9; 55.2]	52.3	[49.0; 55.7]	48.6	[45.3; 51.8]	55.0	[51.7; 58.3]	53.2	[49.3; 57.1]	51.4	[47.7; 55.1]	48.4	[42.3; 54.5]
**Chad**	2014	58.0	[53.4; 62.5]	69.4	[66.5; 72.4]	65.3	[62.0; 68.7]	69.3	[66.1; 72.5]	68.8	[66.0; 71.5]	67.0	[61.6; 72.3]	55.8	[49.0; 62.7]
**Congo Brazzaville**	2014	31.4	[27.3; 35.5]	52.3	[49.4; 55.2]	34.7	[31.0; 38.4]	43.1	[39.3; 47.0]	46.2	[38.5; 53.9]	43.6	[38.8; 48.4]	38.6	[34.6; 42.6]
**Congo DR**	2013	28.2	[23.7; 32.8]	36.5	[31.5; 41.5]	31.2	[26.7; 35.6]	37.0	[32.7; 41.2]	36.1	[29.9; 42.3]	38.5	[33.5; 43.6]	26.9	[22.6; 31.2]
**Côte D'Ivoire**	2016	33.4	[28.3; 38.6]	37.9	[34.7; 41.2]	34.3	[30.8; 37.8]	38.1	[34.7; 41.4]	38.3	[35.2; 41.4]	34.9	[29.5; 40.3]	28.2	[21.7; 34.7]
**Gambia**	2010	29.8	[25.9; 33.7]	30.8	[27.5; 34.1]	27.8	[24.7; 30.8]	32.9	[29.7; 36.1]	31.5	[28.5; 34.5]	33.1	[27.4; 38.7]	23.5	[17.9; 29.0]
**Ghana**	2011	20.2	[16.3; 24.1]	28.4	[24.9; 32.0]	21.3	[17.7; 24.9]	28.4	[24.9; 31.8]	30.1	[26.1; 34.0]	24.6	[19.3; 29.8]	20.6	[16.6; 24.5]
**Guinea**	2016	45.9	[41.0; 50.8]	52.8	[49.5; 56.0]	49.2	[45.8; 52.6]	51.8	[48.6; 55.1]	51.9	[49.0; 54.9]	46.6	[40.4; 52.8]	46.1	[39.9; 52.3]
**Guinea Bissau**	2014	39.9	[33.7; 46.2]	36.4	[33.0; 39.9]	32.6	[28.7; 36.5]	42.9	[39.0; 46.8]	38.8	[35.3; 42.4]	38.8	[33.6; 44.0]	31.7	[24.7; 38.6]
**Mali**	2015	33.3	[29.1; 37.4]	38.0	[35.7; 40.4]	35.1	[32.6; 37.5]	39.0	[36.6; 41.4]	38.4	[36.1; 40.6]	34.1	[30.0; 38.1]	35.9	[29.4; 42.4]
**Mauritania**	2015	34.6	[31.1; 38.1]	41.3	[38.3; 44.3]	36.6	[33.8; 39.5]	40.3	[37.1; 43.5]	41.1	[37.1; 45.2]	41.9	[38.3; 45.6]	37.8	[34.2; 41.5]
**Nigeria**	2016	24.9	[21.5; 28.3]	43.6	[41.6; 45.6]	36.7	[34.6; 38.9]	39.0	[36.9; 41.0]	50.3	[47.2; 53.3]	35.0	[31.8; 38.3]	22.9	[20.8; 25.0]
**S Tome and Principe**	2014	46.1	[40.2; 52.0]	42.7	[36.8; 48.6]	44.1	[38.7; 49.5]	45.8	[39.8; 51.8]	50.3	[33.7; 66.9]	47.1	[42.3; 51.9]	39.6	[31.7; 47.5]
**Sierra Leone**	2010	50.3	[44.8; 55.7]	55.4	[52.2; 58.6]	53.8	[50.4; 57.3]	54.2	[50.8; 57.5]	56.0	[53.1; 59.0]	52.2	[46.2; 58.1]	42.5	[36.0; 49.1]
**Togo**	2013	40.8	[36.3; 45.3]	47.5	[43.6; 51.5]	43.0	[39.4; 46.7]	47.4	[43.4; 51.3]	48.5	[44.1; 52.8]	44.1	[39.8; 48.5]	40.1	[34.7; 45.4]
**Eastern & Southern Africa**															
**Burundi**	2016	44.7	[39.3; 50.0]	60.6	[58.4; 62.8]	53.3	[50.5; 56.1]	65.1	[62.7; 67.5]	63.1	[60.5; 65.7]	58.3	[55.5; 61.1]	41.9	[36.5; 47.3]
**Eswatini**	2014	25.5	[18.5; 32.5]	35.7	[31.8; 39.6]	31.9	[27.3; 36.6]	35.3	[30.6; 40.0]	39.7	[30.6; 48.8]	36.2	[30.7; 41.7]	31.0	[26.6; 35.5]
**Malawi**	2013	28.9	[24.2; 33.5]	40.4	[38.6; 42.2]	35.0	[32.6; 37.4]	43.2	[41.0; 45.4]	47.4	[43.2; 51.6]	39.4	[37.5; 41.3]	28.9	[25.1; 32.6]
**Rwanda**	2014	22.4	[15.6; 29.3]	29.5	[26.9; 32.0]	26.1	[23.1; 29.2]	30.5	[27.5; 33.6]	36.3	[31.1; 41.5]	28.3	[25.6; 31.0]	15.8	[11.4; 20.2]
**Uganda**	2016	22.5	[18.5; 26.6]	37.9	[35.7; 40.1]	33.7	[31.2; 36.2]	35.9	[33.3; 38.4]	40.1	[35.3; 44.9]	38.6	[36.2; 41.0]	22.7	[19.5; 25.8]
**Zimbabwe**	2014	34.4	[31.3; 37.6]	37.6	[35.4; 39.8]	34.4	[32.0; 36.8]	39.4	[36.9; 41.9]	34.7	[26.9; 42.4]	37.5	[34.6; 40.3]	36.6	[34.3; 38.9]
**Middle East & North Africa**															
**Algeria**	2012	28.7	[26.5; 31.0]	30.1	[27.1; 33.0]	25.8	[23.5; 28.2]	32.4	[30.2; 34.7]	33.7	[30.3; 37.2]	29.7	[26.1; 33.4]	27.7	[25.4; 29.9]
**Iraq**	2011	26.6	[24.7; 28.4]	29.8	[27.7; 31.9]	23.7	[22.0; 25.5]	31.5	[29.6; 33.4]	31.7	[29.0; 34.4]	28.7	[26.8; 30.6]	23.7	[21.4; 26.0]
**Jordan**	2012	30.3	[26.5; 34.2]	30.8	[27.4; 34.2]	26.8	[23.0; 30.5]	33.9	[29.6; 38.2]	30.0	[18.8; 41.2]	37.0	[27.1; 46.8]	29.9	[26.5; 33.3]
**State of Palestine**	2014	27.0	[25.2; 28.8]	23.6	[19.6; 27.5]	21.8	[19.7; 24.0]	30.8	[28.6; 33.1]	16.7	[0.7; 32.7]	31.0	[27.8; 34.1]	24.2	[22.4; 26.1]
**Tunisia**	2011	19.6	[16.0; 23.2]	26.4	[21.3; 31.5]	17.8	[14.2; 21.4]	26.1	[21.8; 30.4]	30.1	[22.4; 37.8]	23.1	[18.2; 28.1]	18.9	[15.1; 22.8]
**Europe & Central Asia**															
**Belarus**	2012	4.4	[3.1; 5.7]	8.1	[4.2; 12.1]	3.7	[2.3; 5.1]	7.2	[4.5; 9.9]	NA	-	14.7	[0.0; 29.9]	5.1	[3.6; 6.6]
**Bosnia and Herzegovina**	2011	2.1	[0.6; 3.6]	3.9	[1.4; 6.5]	2.3	[1.1; 3.5]	4.4	[1.1; 7.6]	0.0	-	3.4	[1.3; 5.5]	3.3	[1.1; 5.5]
**Kazakhstan**	2015	12.6	[10.2; 15.0]	13.5	[9.5; 17.6]	12.5	[8.6; 16.5]	13.6	[11.2; 16.1]	0.0	-	25.0	-	13.3	[10.6; 15.9]
**Kosovo**	2013	16.7	[11.8; 21.7]	15.3	[11.9; 18.7]	11.7	[8.1; 15.3]	19.6	[15.1; 24.1]	4.8	[0.0; 14.6]	31.5	[17.1; 45.9]	15.0	[12.1; 17.9]
**Kyrgyzstan**	2014	20.0	[15.4; 24.6]	19.6	[16.6; 22.6]	17.6	[14.3; 20.9]	21.7	[18.4; 25.0]	100.0	-	18.8	[0.0; 48.2]	19.3	[16.6; 21.9]
**Macedonia**	2011	7.6	[4.4; 10.7]	6.3	[2.6; 10.0]	6.8	[3.7; 9.9]	7.1	[3.2; 10.9]	8.6	[0.0; 24.0]	8.3	[3.4; 13.3]	6.0	[3.4; 8.6]
**Moldova**	2012	12.3	[8.8; 15.7]	16.7	[12.3; 21.2]	14.6	[10.3; 18.8]	15.4	[11.0; 19.8]	NA	-	0.0	-	14.4	[11.2; 17.5]

**Montenegro**	2013	4.5	[1.8; 7.1]	4.3	[1.6; 7.1]	3.6	[1.1; 6.2]	5.0	[2.0; 8.1]	0.0	-	7.8	[1.1; 14.4]	3.9	[1.9; 5.9]
**Turkmenistan**	2015	7.1	[4.7; 9.5]	8.0	[5.9; 10.0]	7.6	[5.3; 9.8]	7.8	[5.7; 9.9]	NA	-	0.0	-	7.7	[6.0; 9.4]
**Ukraine**	2012	9.0	[6.6; 11.4]	10.1	[7.0; 13.2]	8.9	[6.4; 11.5]	9.7	[7.1; 12.3]	NA	-	NA	-	9.3	[7.3; 11.2]
**South Asia**															
**Bangladesh**	2012	28.3	[24.7; 32.0]	37.1	[35.6; 38.6]	33.2	[31.3; 35.2]	37.4	[35.5; 39.2]	42.8	[40.2; 45.4]	39.8	[37.4; 42.3]	27.7	[25.8; 29.6]
**Bhutan**	2010	22.1	[17.8; 26.5]	29.5	[26.3; 32.7]	24.0	[20.9; 27.1]	30.5	[27.1; 33.9]	28.9	[25.8; 32.0]	31.5	[25.0; 37.9]	18.5	[14.2; 22.8]
**Nepal**	2014	15.9	[11.5; 20.3]	38.0	[34.1; 42.0]	32.8	[27.9; 37.8]	37.3	[33.2; 41.4]	43.9	[38.9; 48.9]	34.5	[28.3; 40.8]	23.0	[18.6; 27.4]
**East Asia & the Pacific**															
**Cambodia**	2014	18.0	[14.1; 22.0]	28.0	[25.4; 30.5]	27.0	[24.0; 30.0]	26.3	[23.3; 29.3]	32.0	[26.6; 37.5]	27.4	[24.4; 30.4]	22.3	[18.6; 26.0]
**Lao**	2011	11.9	[9.0; 14.7]	20.1	[18.1; 22.0]	16.9	[15.0; 18.9]	19.9	[17.8; 22.0]	25.7	[22.7; 28.7]	16.4	[14.5; 18.4]	11.0	[8.7; 13.2]
**Mongolia**	2013	23.0	[20.4; 25.5]	23.4	[20.7; 26.2]	20.1	[17.7; 22.5]	26.2	[23.3; 29.1]	18.0	[12.3; 23.7]	23.3	[17.1; 29.6]	23.5	[21.4; 25.6]
**Thailand**	2015	6.3	[4.1; 8.6]	7.0	[5.2; 8.7]	5.9	[4.2; 7.7]	7.5	[5.5; 9.4]	15.5	[5.4; 25.6]	7.5	[5.0; 10.1]	7.0	[4.8; 9.1]
**Vietnam**	2013	8.0	[5.4; 10.6]	10.8	[8.0; 13.6]	9.1	[6.5; 11.7]	10.9	[7.8; 13.9]	23.0	[13.0; 33.1]	15.6	[10.0; 21.2]	7.5	[5.4; 9.5]
**Latin America & Caribbean**															
**Argentina**	2011	13.2	[11.4; 15.0]	NA	-	11.3	[8.9; 13.7]	14.9	[12.2; 17.6]	31.6	[5.6; 57.7]	18.0	[13.1; 22.9]	11.4	[9.5; 13.2]
**Belize**	2015	12.1	[8.4; 15.7]	17.7	[13.9; 21.6]	13.0	[9.9; 16.1]	18.1	[13.9; 22.4]	23.2	[8.9; 37.5]	17.2	[12.9; 21.6]	14.6	[10.0; 19.2]
**Costa Rica**	2011	16.8	[10.3; 23.3]	19.9	[14.1; 25.7]	16.8	[10.7; 23.0]	19.5	[13.0; 26.0]	42.7	[11.8; 73.6]	24.8	[17.7; 31.9]	13.7	[8.8; 18.5]
**Dominican Republic**	2014	13.7	[12.2; 15.2]	17.0	[14.8; 19.3]	13.1	[11.4; 14.9]	15.9	[14.0; 17.7]	25.3	[18.8; 31.9]	16.8	[14.6; 19.0]	12.7	[11.2; 14.3]
**El Salvador**	2014	17.2	[14.9; 19.6]	19.4	[16.9; 21.8]	16.3	[14.0; 18.5]	20.1	[17.5; 22.6]	24.1	[17.0; 31.2]	21.7	[18.7; 24.6]	15.2	[13.1; 17.3]
**Guyana**	2014	10.2	[6.2; 14.1]	11.8	[9.1; 14.5]	10.5	[8.1; 12.8]	12.2	[8.7; 15.7]	11.5	[3.9; 19.0]	20.3	[14.1; 26.5]	9.7	[7.4; 11.9]
**Jamaica**	2011	8.9	[5.9; 12.0]	11.4	[6.7; 16.0]	6.1	[3.2; 8.9]	13.4	[9.3; 17.5]	NA	-	7.1	[0.0; 17.1]	10.1	[7.4; 12.9]
**Mexico**	2015	17.1	[14.0; 20.3]	17.7	[14.7; 20.8]	14.2	[10.5; 17.8]	21.1	[18.3; 23.8]	21.7	[10.1; 33.3]	18.5	[14.7; 22.3]	16.8	[13.8; 19.9]
**Panama**	2013	17.9	[12.8; 22.9]	20.6	[16.4; 24.8]	18.3	[14.4; 22.2]	19.4	[14.6; 24.3]	29.3	[21.6; 37.0]	21.9	[17.0; 26.7]	17.0	[12.6; 21.4]
**Paraguay**	2016	17.7	[14.3; 21.0]	16.6	[13.3; 19.8]	15.8	[12.5; 19.0]	18.6	[15.1; 22.1]	23.0	[10.1; 35.9]	22.8	[18.4; 27.3]	21.8	[15.2; 28.3]
**Suriname**	2010	22.1	[17.6; 26.6]	38.6	[34.9; 42.4]	24.0	[20.1; 27.9]	34.2	[29.6; 38.8]	43.1	[36.3; 49.8]	31.9	[27.1; 36.7]	22.4	[18.0; 26.7]
**Trinidad and Tobago**	2011	6.9	[3.1; 10.7]	5.0	[2.2; 7.8]	4.9	[1.7; 8.0]	7.3	[3.4; 11.2]	11.2	[0.0; 38.6]	8.7	[2.9; 14.6]	5.5	[2.5; 8.4]
**Uruguay**	2012	13.0	[6.1; 19.9]	4.0	[0.0; 9.4]	14.6	[5.3; 23.9]	11.2	[4.9; 17.5]	NA	-	21.2	[2.1; 40.3]	9.5	[4.8; 14.2]

Note: Data not available marked as NA
